# Tiny wasps, huge diversity – A review of German Pteromalidae with new generic and species records (Hymenoptera: Chalcidoidea)

**DOI:** 10.3897/BDJ.9.e77092

**Published:** 2021-12-07

**Authors:** Michael Haas, Hannes Baur, Tanja Schweizer, Juan Carlos Monje, Marina Moser, Sonia Bigalk, Lars Krogmann

**Affiliations:** 1 Entomology, State Museum of Natural History, Stuttgart, Germany Entomology, State Museum of Natural History Stuttgart Germany; 2 Systematic Entomology (190n), University of Hohenheim, Stuttgart, Germany Systematic Entomology (190n), University of Hohenheim Stuttgart Germany; 3 Department of Invertebrates, Natural History Museum Bern, Bern, Switzerland Department of Invertebrates, Natural History Museum Bern Bern Switzerland; 4 Institute of Ecology and Evolution, University of Bern, Bern, Switzerland Institute of Ecology and Evolution, University of Bern Bern Switzerland

**Keywords:** parasitoid wasps, biodiversity, distribution, DNA barcoding, German Barcode of Life, dark taxa

## Abstract

**Background:**

Despite their ecological and economic importance, hymenopteran parasitoids are severely understudied. Even in countries with a long taxonomic history such as Germany, dating back to the 18th century and including prolific figures like Christian Gottfired Nees von Esenbeck and Otto Schmiedeknecht, those species-rich groups are seldom the subject of comprehensive research efforts, leaving their true diversity unknown. This is often due to their small size of a few millimetres on average, leading to difficulties in their identification and examination. The chalcidoid family Pteromalidae is no exception to this neglect. So far, 735 species have been reported from Germany. Estimating the diversity of this group is not possible, but it has to be assumed that many more species are still to be discovered in Germany.

**New information:**

With this study, we improve the knowledge on pteromalid diversity and present new records of 17 genera and 41 species, previously unknown to occur in Germany. We also match and describe previously unknown sexes of two species, based on DNA barcode data. The results of this study were generated as part of the German Barcode of Life Project. The newly-recorded species are illustrated and notes on the biology and distribution are given. The ecological significance of Pteromalidae and potential value as indicators for nature conservation efforts are briefly discussed.

## Introduction

Insects are key organisms in our natural world, fulfilling not only important ecosystem functions, but also providing beneficial ecosystem services for mankind (Weisser and Siemann 2008, Noriega et al. 2018). Though this is largely known, insects are often highly understudied with some taxa potentially being vastly more species-rich than expected, especially in Hymenoptera and Diptera (Hebert et al. 2016, Morinière et al. 2019). Within Hymenoptera, the parasitoid taxa are a particularly prominent example, with a large number of species still to be discovered and their biology unknown (Forbes et al. 2018). They are vital for the world's ecosystems in terms of population regulation of their hosts and many of their better known representatives are used as biological control agents in agriculture (Quicke 1997). In nature conservation efforts, they are not taken into account, lacking representation on Red Lists and gaining hardly any recognition as potential environmental indicators (Shaw and Hochberg 2001, Anderson et al. 2011). Chalcidoidea are one of the most species-rich groups within parasitoid Hymenoptera, with about 22,500 described species worldwide and estimates ranging up to 500,000 species in total (Heraty and Gates 2003, Noyes 2021). This mismatch between described and estimated diversity further highlights the lack of scientific research dedicated to those groups. In Germany, close to 2,000 chalcidoid species have been recorded (Noyes 2021, Zoologische Staatssammlung München 2021). Pteromalidae is the most numerous chalcidoid family with 735 known species (Doczkal 2017, Noyes 2021).

**Figure 1.**
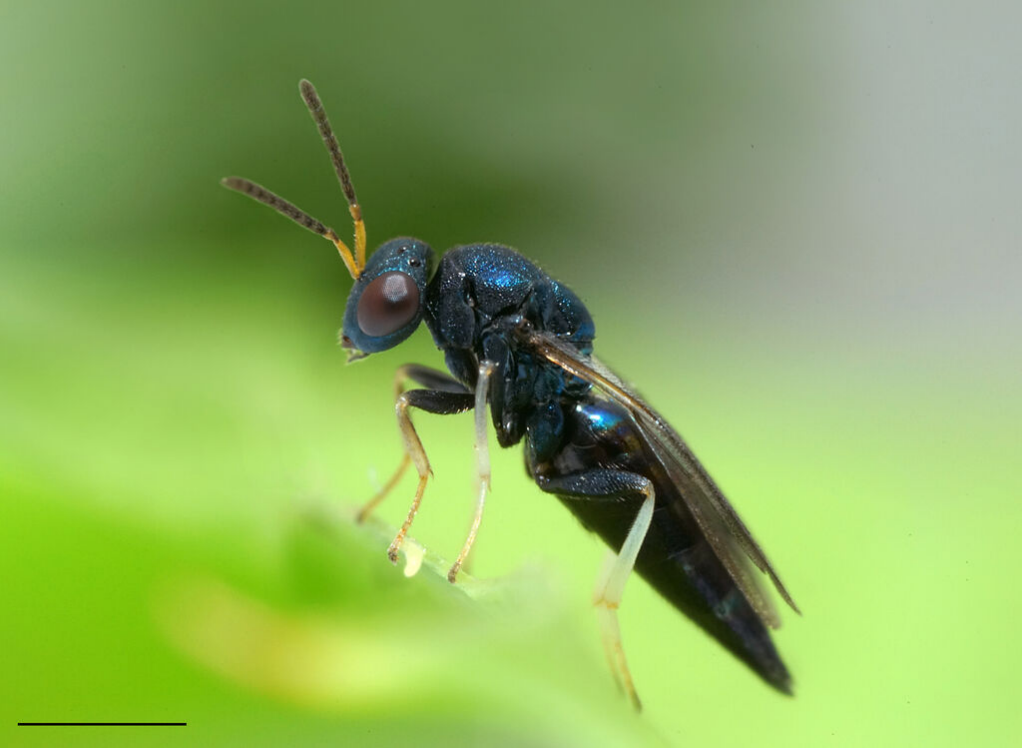
*Cecidostibadocimus* (Walker, 1839), a representative of the family Pteromalidae. Scale bar: 1 mm. Image credit: Aron Bellersheim.

The family Pteromalidae (representative shown in Fig. 1) is polyphyletic with over thirty subfamilies scattered all throughout the chalcidoid phylogenetic tree (Munro et al. 2011, Heraty et al. 2013, Peters et al. 2018, Zhang et al. 2020). Subfamilies within Pteromalidae are mostly well-diagnosed morphologically and monophyletic, but the family, as a whole, lacks synapomorphic characters (Graham 1969, Bouček 1988, Bouček and Rasplus 1991, Grissell and Schauff 1997). Noyes (2021) lists characters like the five segmented tarsi, a generally metallic body, 8-13 segmented antennae with up to three annelli, developed wing venation with, in most cases, an elongate marginal, postmarginal and stigmal vein, a mostly well-developed speculum and a body size of 1-48 mm. Those features are often widely shared amongst other chalcidoid families, therefore lacking diagnostic value. Pteromalidae will have to be systematically revised in the future to resolve its polyphyletic nature. In addition to that, pteromalid species are often described from a single sex, predominantly females, because they usually exhibit a higher morphological distinction. Males are rather morphologically uniform and the existence of sexual dimorphism often prohibits matching sexes, unless more information about their biology is known and larger series of rearings of their hosts are conducted (Gibson and Reigada 2009). With molecular data, however, even extreme cases of dimorphism can be resolved, like in the pteromalid subfamily Sycoryctinae, where males and females show drastically different morphology (Zhou et al. 2012).

### Review of sampling methods for Pteromalidae

Pteromalidae are known from all regions in Germany and collecting these tiny wasps with an average (body) size of roughly 2-3 mm can be done in many ways. There is already some helpful literature about collecting chalcidoid wasps, including, for example, Noyes (1982), Bouček (1988), Grissell and Schauff (1997), Noyes (2021), which also reference other resources for further reading. Here, only a short synopsis of sampling and preparation methods will be given. Due to their size, Pteromalidae can hardly be observed and sampled on sight; therefore, the most effective methods of sampling include active methods like sweep netting, as well as trapping and rearing. Sweep netting is an easy, widely applied method and if done mindfully, it can give some additional information on plants and habitats, from where specimens have been sampled for biological implications. It is generally advised to use a sturdy net, with strong fabric to withstand beating through vegetation like meadows and shrubs. Noyes (1982) designed a very suitable net for this purpose, being subtriangular in form and heavily reinforced. Its form allows it to be swept just above the ground, catching species, largely dwelling close to the ground. It is also fitted with a screen to avoid large amounts of vegetation and debris getting inside the net, making it easier to recover caught specimens. Collecting specimens out of the net sack is best done by using an aspirator with a long tube to precisely pick out individuals. Keeping the net opening away from the sun and upright forces specimens to walk to the top of the net, allowing for easier aspiration. As a euthanising agent, pure and highly concentrated (99.9%) ethanol is advised, as it preserves the specimens and guarantees DNA stability for subsequent molecular analysis. Other effective, but rather rare, active sampling methods for chalcidoids include suction samplers, vegetation beating, light tower collecting and advanced chemical techniques, like canopy fogging.

In contrast to active sampling methods, trapping techniques allow for a neutral assessment of biodiversity, free of collector bias, for example, for monitoring purposes, but with the caveat of catching non-target organisms, requiring additional sorting time to separate the groups of interest. The most widely used method of trapping flying insects is the malaise trap (Townes 1972). Correctly set up, this flight interception trap will sample autonomously, requiring little maintenance, but only regular changing of the collecting jar. Intervals of changing the jar depend on the scientific question, but it is advised to not let too much time pass, because evaporation, influence of the sun and temperature might damage collected material when left in the collecting liquid for too long. Again, pure and highly concentrated ethanol is advised as the euthanising agent, but it might be diluted to about 80% for the purpose of biomass assessment (Ssymank et al. 2018). Other flight interception traps, like window traps are also reliable methods to catch microhymenoptera. Colour pans are a more common way of collecting parasitoid wasps as well, although they need more attention and care when being deployed in the field and are more selective due to their attracting properties (Buffington et al. 2021). Light trapping is a rather unexplored method, but might also be useful to assess pteromalid diversity. Pitfall traps are rarely used, but can yield rarely collected, ground dwelling species. When trying to assess the complete diversity of parasitoid wasps, it would be recommended to use several sampling techniques.

In order to gain specific biological information about a species, rearing is an ideal method; however, it is fairly time consuming. Everything potentially harbouring parasitoids can be collected, such as plant galls, dead wood, insect pupae, egg masses, parts of plants, potential hosts and many more. Collected material should be kept in containers that allow for enough airflow to prevent mould, but preventing the emerging wasps from escaping. Tightly sealable plastic boxes fitted with fine mesh have proven to be an easy and cheap way to store material. In some cases, it can be useful to keep the material in the container slightly moist, to keep the insects inside from drying out and dying. Material should ideally be stored in a natural climate in order to allow parasitoids to develop normally. Containers need to be checked regularly to catch emerging specimens alive, in order to preserve them and avoid collapsing body parts when drying in air. If possible, it is advised to separate hosts to gain more biological information, because often several potential host taxa might be present.

Storing samples can easily be done by leaving them in pure and highly concentrated ethanol. If molecular analyses are to be performed, freezing the samples at -20°C is advised, although this might affect morphological studies negatively (Marquina et al. 2021).

Before drying and mounting, specimens often have to be presorted. This can be especially tedious with trapping samples containing many non-target individuals. Automatic sieving (Buffington and Gates 2008) has proven to be extremely useful and effective, presorting specimens by size. Pteromalidae will mainly be found in the smaller fraction and can be sorted out more easily, without the risk of damage while sieving, due to their strong sclerotisation.

It is uncommon to slide-mount Pteromalidae; instead, the most widely used method of mounting is card mounting. For this method, it is necessary to dry specimens gently before mounting, either by using critical point drying (Gordh and Hall 1978) or chemical drying (Heraty and Hawks 1998), preventing collapsing bodies and deformed extremities. There are several techniques for card mounting, depending on preference (Noyes 2021). Gluing specimens on the point of triangular cards or on top of rectangular cards appears to be best practice as it allows us to view the specimen from all sides. This is especially true for point mounting, although the specimens are more in danger of being damaged when handled without care. Deans (2018) provides an in-depth review of potential glues for entomotaxy; however, with no clear recommendation. Choice is therefore dependent on the needs of the user. Although not recommended by Deans (2018), shellac is used by the authors to great effect, as it has the ideal properties and mounting results are excellent. Mounted specimens should be stored in air-tight insect drawers under dark and climate stable conditions in order to preserve colours and avoid damage.

### Review of taxonomic research on Pteromalidae in Germany

Species now part of Pteromalidae have already been described by Carl von Linné as early as 1758 in his “Systema naturae”, with many scientists following in the centuries after. Dalman, a Swedish naturalist, first coined the taxonomic term “Pteromalini” in 1820 as a family, including many taxa today being part of other chalcidoid families or other superfamilies (Dalman 1820). In comparison to other parasitoid hymenopteran families, Pteromalidae are rather well recorded from Germany, but actual species numbers can only be estimated. A review on the historic research of parasitoid wasps in Germany is given by Vidal (2005), including the pre-Linean era. Here, a brief history of work on Pteromalidae in Germany after Linné will be given in the following, although it has to be noted that larger taxonomic and systematic works on Pteromalidae were mostly conducted outside of Germany (Walker 1839a, Walker 1839b, Thomson 1876, Thomson 1878, Ashmead 1904, Graham 1969, Bouček 1988).

Johan Christian Fabricius, a Danish zoologist, mostly focused on studying arthropods and was probably the first to conduct work on Chalcidoidea and Pteromalidae in Germany. Like his mentor Carl von Linné, Fabricius dedicated much of his work to describing species in several large monographs (e.g. Fabricius 1775, Fabricius 1787, Fabricius 1793). This also included several species of Chalcidoidea, part of the German fauna, for example, the morphologically remarkable *Leucospisdorsigera* Fabricius, 1775, the striking pteromalid *Cratomusmegacephalus* (Fabricius, 1793) or *Cheiropachusquadrum* (Fabricius, 1787), which was described from material collected in Halle, Germany. Fabricius curated several collections; therefore, specimens he described and worked with are dispersed between different institutions, with the largest parts housed in the Zoological Museum in Copenhagen, the Zoological Museum at Kiel University and the Natural History Museum in London (Tuxen 1967). Although his descriptions were not comprehensive and hardly informative by today’s standards, it can only be imagined how difficult it must have been to study organisms of few millimetres at that time. This achievement is also highlighted by contemporary artwork, as is shown in Panzers "Fauna insectorum Germaniae initia", a book series of German insects, published between 1796 and 1813.

One of the first to publish a comprehensive list of German parasitoid wasps, included in a record of insects in general, was Nikolaus Joseph Brahm (1790). Only 20 species of *Ichneumon* are listed in his work, a genus which, at the time, included many parasitoid wasp taxa, which belong to several different superfamilies today. Therein included is also *Ichneumonpuparum* Linnaeus, 1758, known today as *Pteromaluspuparum* (Linnaeus, 1758), one of the oldest formally described Pteromalidae by Linné. Brahm did not describe any species anew, but provided a rudimentary key and scarce notes on the biology of the *Ichneumon* wasps. In 1802, Franz von Paula Schrank published a regional list of Bavarian insects, also encompassing species of the Genus *Ichneumon* (Schrank 1802). Schrank also listed biological information for each species, where possible, but did not describe new species of Pteromalidae within his work.

In 1834, the remarkable German naturalist Christian Gottfried Daniel Nees von Esenbeck published his opus, the two volumes of “Hymenopterorum Ichneumonibus affinium monographiae”. In those monographs, Nees von Esenbeck provided descriptions and keys to a multitude of species, of which many, although synonymised over the years, are still part of the German fauna. Nees von Esenbeck’s important collection of Hymenoptera was reportedly severely damaged, but its remains are now part of the collection of the University Museum in Oxford, UK (Graham 1988). Seven years after Esenbecks publication in 1841, Arnold Foerster published the “Beiträge zur Monographie der Pteromalinen Nees.” adding to the work of Nees von Esenbeck, whom he praises"… as one of the greatest naturalists of our time…" (Foerster 1841). Within this, he gives a short historic overview of pteromalid research, lists known biological observations of different species and provides a key and descriptions to new species, regarded at that time to be part of Pteromalidae. Foerster followed this work up with several publications on the taxonomy and systematics of Chalcidoidea and Pteromalidae (Foerster 1856a, Foerster 1856b, Foerster 1868, Foerster 1878), describing many new species from Germany. His collection of parasitoid wasps is now part of the collection of the Natural History Museum in Vienna (Hörn 1928). Although the taxonomy and systematics of Pteromalidae and Chalcidoidea would change drastically since then, it is apparent that profound examinations were conducted even at that time, to shed light on those tiny wasps that "… allow us to take a deep look into the proceedings and liveliness of nature." (freely translated from Foerster 1841). Foerster’s work was later partly updated by the Austrian naturalist Karl Wilhelm von Dalla Torre, renaming some of his described species due to homonymy (Dalla Torre 1898).

At around the same time of Foerster’s workings, Julius Theodor Christian Ratzeburg also published on Chalcidoidea. Ratzeburg was a German naturalist mainly interested in forestry, but some of his work focused heavily on parasitoid wasps, especially with regard to forestry pests. Over the years, he published several volumes which included biological observations and species descriptions of chalcidoid wasps amongst other taxa (Ratzeburg 1844a, Ratzeburg 1844b, Ratzeburg 1848). Unfortunately, his collection, including type material, appears to have largely been destroyed during the Second World War, with only few remnants remaining in the Deutsches Entomologisches Institut (Königsmann 1964, Vidal 2005), now situated in Müncheberg.

Another noteworthy German hymenopterist partly working on Pteromalidae and Chalcidoidea at the beginning of the 20^th^ century was Otto Schmiedeknecht (e.g. Schmiedeknecht 1907, Schmiedeknecht 1909). His book chapter on the family Chalcididae in 1909 (Schmiedeknecht 1909) was critically received. It was viewed to be a faulty copy of Ashmeads’ comprehensive publication in 1904 on the superfamily of Chalcidoidea (Ashmead 1904) and was even being described as "… a tragedy and a comedy of errors" (Girault 1910). Nonetheless, Schmiedeknecht continued his work and, in 1930, provided an update of his extensive catalogue on the Hymenoptera of northern and central Europe, the first version of which he had published 16 years prior. Therein, he listed all occurring genera of Pteromalidae and provided comprehensive keys for their identification (Schmiedeknecht 1914, Schmiedeknecht 1930). His collection, although largely focused on Ichneumonidae and Anthophila, is severely torn apart, but an exhaustive account is given by Oehlke (1968) of its whereabouts.

In the middle of the 20^th^ century, the Italian field entomologist Vittorio Delucchi described many pteromalid species and genera from Western Europe including Germany. An encompassing account of his described species including literature is given by Baur (2001).Numerous of those species were described from holotypes collected in Germany, either by Delucchi himself or from historic collections, for example, Foerster or Schmiedeknecht. The whereabouts of Delucchis’ collection are also detailed in Baur (2001).

Marcus William Robert de Vere Graham should be named here as an entomologist who made the vastness of European Pteromalidae more widely accessible through his work. Especially noteworthy here is his monograph from 1969 on the Pteromalidae of north-western Europe (Graham 1969). Therein, he provides keys to subfamilies, genera and even species, with detailed information on type material, distributions and where available, biological information. His work is, to this date, mostly the first to consult when identifying Pteromalidae from Germany and Europe. Zdeněk Bouček was a contemporary of Graham, dedicating his research to the superfamily Chalcidoidea, with Pteromalidae being one of the main focuses of his work (Noyes 2005). A full list of his publications can be found in Noyes (2005), which, in great parts, is also relevant when studying the German pteromalid fauna. His and Jean-Yves Rasplus “Illustrated key to West-Palearctic Genera of Pteromalidae” has to be mentioned here specifically, as it is a beginner-friendly introduction to pteromalid identification, aided by line drawings and SEM images (Bouček and Rasplus 1991).

After the middle of the 20^th^ century, research focusing on and including Pteromalidae in Germany diversified, with some examples of different researchers and working groups listed in the fields of ecology (Abraham 1970, Kruess and Tscharntke 1994, Kruess 1996, Tscharntke et al. 2001), taxonomy (Werner and Peters 2018), morphology (Krogmann and Abraham 2003, Krogmann 2005, Vilhelmsen et al. 2010), phylogenetic analysis (Heraty et al. 2013, Peters et al. 2018), molecular studies (König et al. 2019) and autecology (Abraham and König 1977, Steidle and Schöller 1997, Ruther and Steidle 2000, Niehuis et al. 2013, König et al. 2015, Niedermayer et al. 2016, Tappert et al. 2017).

In 2001, the most recent species list of German Hymenoptera was published, listing 663 species of Pteromalidae (Vidal 2001). Since then, the number of species has been tracked by web-based databases, for example, the Chalcidoidea database (Noyes 2021) and the thereon-based Checklist of the German Chalcidoidea (Zoologische Staatssammlung München 2021), listing 734 pteromalid species or the German Barcode of Life taxon lists (German Barcode of Life Consortium et al. 2011), based on Fauna Europea (de Jong et al. 2014), listing 695 recorded species. The reasons for the discrepancy in species numbers are unknown, but it has to be speculated that the Chalcidoidea database was curated regularly until March 2019; therefore, it is potentially the best source for the most reliable species number. Only one species (Doczkal 2017) was not yet added, resulting in a total of 735 pteromalid species recorded from Germany to date.

### Pteromalidae in the German Barcode of Life project

In more recent history, the German Barcode of Life project (German Barcode of Life Consortium et al. 2011) was initiated, aiming to inventory the German fauna and flora in the form of a barcode database, also including Pteromalidae. With the advent of metabarcoding methods of insect material (e.g. Morinière et al. 2016, Toju and Baba 2018, Piper et al. 2019, Liu et al. 2020), barcoding databases will be heavily relied upon in the future, especially for the identification of morphologically diverse and difficult groups. This results in the necessity to fill the databases with correct and comprehensive data. Geiger et al. (2016) demonstrated that, in Germany, metabarcoding of malaise trap samples yielded an especially low identification success of about 40% for genus and species level in the two most abundant orders of insects, the Diptera and Hymenoptera. In Hymenoptera, especially, many of the alleged parasitoid barcodes could not be assigned to a genus or species identification. This, in combination with their ecological importance and potential use as indicator groups (Shaw and Hochberg 2001, Anderson et al. 2011), highlights the urgency to further advance the molecular databases and continue working on those elusive groups.

In a first step, we here present 41 newly-recorded species for the German fauna, collected and processed within the GBOL project. DNA barcodes are supplied to most of the presented species. In addition, more general information on the family Pteromalidae will be given to exemplify the importance to advance research efforts, further shedding light on this important taxon and parasitoid wasps in general.

## Materials and methods

### Origin and handling of specimens

**Figure 2.**
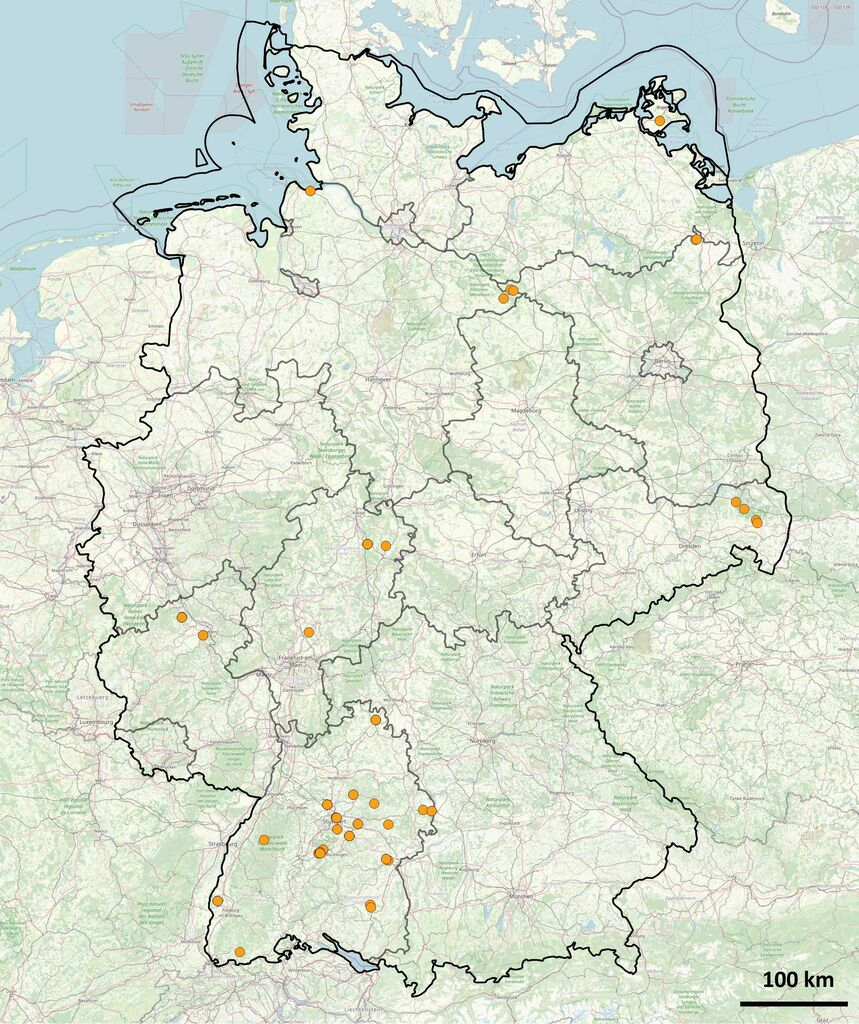
Origin of material studied from Germany. © Bundesamt für Kartographie und Geodäsie, Frankfurt am Main, 2011; OpenStreetMap -contributors.

The pteromalid specimens, used in this study, were collected in different localities throughout Germany (Fig. 2) between the years 2011 to 2021. The specimens treated here (n=122) were collected through various methods, mainly by malaise trapping (n=73), sweep netting (n=39) and others (n=10). Before laboratory procedures were conducted, all specimens were kept in 99.6% pure ethanol at -20°C.

### Barcoding

All molecular work was conducted in the State Museum of Natural History Stuttgart (SMNS), except for sequencing, which was done at Eurofins Genomics, formerly GATC Biotech AG (Germany, Ebersberg).

The DNA extraction protocol, including buffer recipes, was based on the protocol of Ivanova et al. (2006), but modified to suit the small size of the specimens and working with a Xiril Neon 100 pipetting robot. Differences to the original protocol of Ivanova et al. (2006) will be specified in the following. Due to the small size of the specimens, the whole body had to be used for DNA extraction. A semi-destructive approach was chosen to yield enough DNA for analysis, allowing for a morphological specimen voucher. By removing one of the hind legs, an opening was created for the lysis buffer to penetrate the heavily sclerotised body, otherwise hardly permeable for the buffer and the released DNA. Every specimen was submerged in 100 µl ProteinaseK and Insect Lysis Buffer (ILB) mixture within a 1.5 ml tube and incubated at 56°C for about 30 hours in a Thermoshaker, agitating the lysis mix at 200 rpm. Afterwards, the lysate was separated from the specimen and transferred to a 96 format deep well plate for DNA isolation. The isolation was performed by the pipetting robot, utilising a silica-based isolation system. Due to the initially larger amount of lysis buffer, 200 µl of binding mix was used to fix the DNA to the filter. Centrifugation steps from Ivanova et al. (2006) are not realisable in the robot and were substituted via a vacuum pump, removing the residue washing buffers from the membrane-bound DNA. The purified DNA was transferred to an elution plate, which was stored for a short time at -20°C until after the PCR had been conducted. Afterwards, long-term storage at -80°C is provided.

The PCR was conducted using different iterations of the FastGene® Optima Taq from Nippon Genetics. In total 25 µl were used for PCR reaction, including 4 µl eluate DNA and other components mixed to the manufacturers’ recommendation. Primers LCO1490 and HCO2198 (Folmer et al. 1994) were used with the PCR-protocol in the reaction, following the cycler protocol in Table 1. Purification and sequencing of the resulting PCR products was conducted by GATC Biotech AG, now Eurofins Genomics. Final sequences are deposited in the Barcode of Life Data (BOLD) Systems (Ratnasingham and Hebert 2007, Ratnasingham and Hebert 2013), as well as Genbank (Clark et al. 2015) and can be accessed via their respective IDs given in the material section of each specimen.

To match undescribed males to described females, the mean in between group p-distance was calculated with MEGA-X (Kumar et al. 2018). Standard errors were assessed by bootstrap (1000 replicates) and the rate variation amongst sites was modelled with a gamma distribution.

### Mounting of specimens

After lysis, specimens were washed in a soapy water solution to rid them of buffer residue. The solution was discarded and specimens were transferred to a 70% MEK-ethanol solution, which was substituted in steps by higher concentrations (90%, 95% and 99.6%) of MEK-ethanol over the course of several days. For drying and subsequent mounting, specimens were transferred to Hexamethyldisilazan (Heraty and Hawks 1998) and kept in open Eppendorf tubes until they were completely dry. The specimens were mounted on card points using shellac glue, labelled and stored in the entomological collection of the State Museum of Natural History Stuttgart (SMNS) for later identification. Each specimen was given a unique accession number of the format “SMNS_Hym_Pte_XXXXXX'' under which all collected information is stored in the SMNS collection Database “Diversity Workbench” (Triebel et al. 1999). Accession numbers are abbreviated in the text in the format “Pte_XXXXXX”

### Identification

Morphological identification was conducted by multiple taxonomists, predominantly using a Leica M205 C stereomicroscope, with a measuring eyepiece. The main literature used for identification included Bouček and Rasplus (1991) for genus level identification and Graham (1969) for genus and species level identification. If available, more recent literature was used to identify specimens to species level. Information on the distribution outside of Germany and the biology of species was supplemented through consultation of the following references: Graham (1969), Bouček and Rasplus (1991), Noyes (2021). A list with all newly-recorded species is given in Table 2.

### Taxon treatments and checklist of new records

Our results were split into two sections within the manuscript. Firstly, within the taxon treatment section, we want to focus on the description of two previously-undescribed males of the species *Rhicnocoeliaimpar* (Walker, 1836) and *Rohatinainermis* Bouček, 1954. Both species are new records for Germany and are also treated in the faunistic part of our results. Our faunistic data are presented in the checklist section of the manuscript, where detailed information is given on the newly-recorded species and genera of Germany. The checklist is sorted by subfamily and Table 2 can serve as an overview.

## Data resources

DNA barcoding data including the trace files and resulting barcodes are deposited in a publicly-accessible dataset (DS-PTEGBOLR) on the Barcode of Life Data (BOLD) systems platform (Ratnasingham and Hebert 2007), available through the DOI: dx.doi.org/10.5883/DS-PTEGBOLR. Additionally to BOLD Systems, the generated sequences are also deposited in GenBank (Clark et al. 2015) and are available through the GenBank IDs OL538053 - OL538158. Information for single specimens can be retrieved by using the BOLD Sample ID or GenBank ID, respectively, as specified in each specimens material section.

## Taxon treatments

### Rhicnocoelia impar

(Walker, 1836)

F18FA27B-3024-53E1-A7DB-F146B702ADAD

#### Materials

- **Type status:** Other material. **Occurrence:** catalogNumber: BOLD Sample ID: SMNS_40918; recordedBy: I. Wendt; sex: male; lifeStage: adult; preparations: dry mounted; associatedSequences: GenBank: OL538084; **Taxon:** scientificName: *Rhicnocoeliaimpar* (Walker, 1836); **Location:** continent: Europe; country: Germany; countryCode: DE; stateProvince: Hessen; locality: Wetteraukreis, Dorn-Assenheim, Wiese; decimalLatitude: 50.3468; decimalLongitude: 8.8492; **Identification:** identifiedBy: M. Haas; dateIdentified: 2021; **Event:** samplingProtocol: Sweep net; eventDate: 28/9/2014; **Record Level:** datasetID: SMNS_Hym_Pte_003853; institutionCode: SMNS

#### Description

##### Images

Fig. 3

##### Description of male

Colour: Head and mesosoma: mostly green bronze to green, with metallic lustre; setae on head and mesosoma: generally whitish, inconspicuous, setae on top of head rather fuscous to brown; tegula: yellow to testaceous; setae on callus of propodeum: whitish. Scape: green bronze with metallic lustre; pedicel: green bronze with metallic lustre, slightly darker than scape; flagellum: dark brown to black. Fore wing: hyaline; fore wing venation: lightly testaceous; setae on fore wing: fuscous; hind wing: hyaline. Pro-, meso- and metacoxa: green bronze with metallic lustre; trochanters: dark yellow to testaceous; femora: dark yellow to testaceous, dorsomedially with brown to greenish-bronze colouration with metallic lustre; tibiae: yellow to testaceous; protarsae: fuscous, except anterior first tarsal segment being testaceous; meso- and metatarsae: gradually darkening over segments from light yellow to fuscous. Petiole: dark brown, with green bronze metallic tinge; gaster: uniformly dark brown; gastral terga: with green bronze metallic tinge.

Sculpture: Head in frontal view: reticulate with moderately high septa; clypeus: reticulate; area between clypeus and malar sulcus: reticulation uniform with single areoles elongated radiating from clypeus. Mesoscutum: finely reticulate, meshes moderately low, areoles slightly enlarged medially; mesoscutellum: finely reticulate, mesh equivalent in size to mesoscutum; frenum: engraved reticulation, mesh equivalent in size to mesoscutellum; axilla: finely raised reticulate, as strongly as, but mesh size smaller than in mesoscutellum; prepectus upper triangular area: uniformly reticulate; upper mesepimeron: smooth; upper mesepisternum: reticulate, about as strongly as on mesoscutum; metapleuron: finely reticulate, as strong as mesepisternum. Procoxa: engraved reticulate; mesocoxa: engraved reticulate; metacoxa: raised reticulate dorsally to engraved reticulate ventrally. Median area of propodeum: strongly, irregularly reticulate, about as strong as on mesoscutum, with few higher irregular ridges; inner corner of anterior plica: without depression; nucha: not enlarged, presented as a fine rather smooth strip; callus of propodeum: irregularly reticulate, but less strong than median area of propodeum, even partly smooth; paraspiracular sulcus: coarse rugae, continuing from median propodeal area, reticulation hardly present. Petiole in dorsal view: smooth; gastral terga: smooth and shining, from second tergum alutaceous.

Shape and structure: Head in frontal view: round; gena in frontal view: slightly curving; temple in dorsal view: obtuse; occipital carina: absent; torulus position with respect to lower ocular line: distinctly above; lower face in lateral view: slightly rounded, with slight bulge on lower face below toruli; receding with respect to upper face: weakly; scrobe: narrow and shallow; malar sulcus: developed, moderately deep; clypeus anterior margin: three asymmetric teeth, with single tooth on the right, inner left tooth larger than other two; tentorial pit: indistinct; mouth extension: moderate; mandibular formula: not assessable. Antenna: Antennal formula: 11263; scape reaching: hardly median ocellus; flagellum: filiform; first anellus: strongly transverse; second anellus: strongly transverse; first funicular segment: conical, elongate; following funicular segements: conical, elongate; setae on flagellum: fairly thickly clothed with forward pointed erect setae, length of setae hardly as long as breadth of flagellar segments; number of rows of longitudinal sensilla on first funicular segment: 3 - 4 irregular; on sixth: 2 - 3 irregular. Mesosoma in lateral view: moderately bent; propodeum in lateral view slightly angled to mesoscutellum; pronotum breadth with respect to mesoscutum breadth: distinctly narrower; pronotum collar: directly sloping from mesoscutum; its length with respect to mesoscutum length: indiscernible; its anterior margin: not carinate; pronotum posterior margin: forming smooth, shiny strip; notauli: largely superficial, but traceable to hind-margin of mesoscutum; reaching: five sixth along mesoscutum; mesoscutellum in lateral view: slightly arched, especially in posterior third; mesoscutellum in posterior view: rounded; mesoscutellum posterior margin projection: level of anterior margin of metascutellum; mesoscutellum posterior margin in posterior view: appearing ecarinate; frenal line: indicated through row of deeper areoles; prepectus upper triangular area: uniform, but with transverse carina at height of procoxal articulation; upper mesepimeron: almost parallel sides, only tapering at base of mesopleuron; propodeum anterior plica: absent; posterior plica: absent; median carina of propodeum: absent; nucha: reduced to small strip; spiracle: oval, almost round, almost touching anterior margin of propodeum; size: moderate; callus pilosity: thinly pilose; paraspiracular sulcus: steep, angled against median area of propodeum. Forewing: Forewing apex with respect to apex of gaster when folded back: exceeding close to 1/6th its length; basal cell number of setae: few up to 6 distally; basal setal line: incomplete with 3-6 setae; cubital setal line: absent, maximum 3 setae distally; costal cell pilosity on dorsal side: about 6 setae distally; costal setal line: incomplete; speculum on dorsal side: bare, extending close to 1/3rd the length of the marginal vein, widely open below; wing disc: moderately pilose; marginal setae: present, from as long as the length of setae on wing disc on anterior wing margin to about double the length on posterior margin; stigma: oval, small size; uncus: straight, as long as stigma broad. Femora: moderately slender; metatibia: slender, gradually widening towards apex; metacoxa pilosity dorsal: bare. Petiole in dorsal view: subconical; in ventral view: open; gaster in dorsal view: rather club-like, not particularly accuminate; gastral terga: convex, weakly sunken anteriorly; posterior margin of first gastral tergum: emarginate; first gastral tergum reaching: 1/3rd of gaster.

Measurements (n=1): length and body ratios: Body length: 3 mm; mesoscutum breadth: 676 μm. Head breadth to head height: 1.45; head breadth to length: 1.91; head breadth to mesoscutum breadth: 1.66; upper face height to head height: 0.63; POL to OOL: 1.19; eye height to breadth: 1.31; eye distance to height: 1.45; temple length to eye height: 0.30; malar space to eye height: 0.44. Antenna length, including scape to head breadth: 2.48; scape length to eye height: 0.67; pedicel length to breadth: 1.11. Mesosoma length to mesoscutum breadth: 1.89; mesoscutum breadth to length:1.52; mesoscutum length to mesoscutellum length: 1.17; propodeum length to mesoscutellum length: 0.69; plica distance to propodeum length: 1.13. Forewing length to breadth: 2.48; marginal vein to stigmal vein length: 1.98. Metafemur length to breadth: 4.61.

#### Molecular similarity to female in COI

p-distance to female: 0.0110, S.E.: 0.0040

### Rohatina inermis

Bouček, 1954

E6D2CE46-37F7-5A89-B251-96E408C8A93B

#### Materials

- **Type status:** Other material. **Occurrence:** catalogNumber: BOLD Sample ID: SMNS_37345; recordedBy: L. Krogmann, T. Kothe; sex: male; lifeStage: adult; preparations: dry mounted; associatedSequences: GenBank: OL538068; **Taxon:** scientificName: *Rohatinainermis* Bouček, 1954; **Location:** continent: Europe; country: Germany; countryCode: DE; stateProvince: Baden-Württemberg; locality: Strohgäu, Markgröningen, EVS-Grundstück im Leudelsbachtal; verbatimElevation: 270 m; decimalLatitude: 48.9147; decimalLongitude: 9.0864; **Identification:** identifiedBy: L. Krogmann; dateIdentified: 2014; **Event:** samplingProtocol: Malaise trap; eventDate: 5/6 - 19/6/2011; **Record Level:** datasetID: SMNS_Hym_Pte_000282; institutionCode: SMNS
- **Type status:** Other material. **Occurrence:** catalogNumber: BOLD Sample ID: SMNS_37853; recordedBy: L. Krogmann, T. Kothe; sex: male; lifeStage: adult; preparations: dry mounted; associatedSequences: GenBank: OL538090; **Taxon:** scientificName: *Rohatinainermis* Bouček, 1954; **Location:** continent: Europe; country: Germany; countryCode: DE; stateProvince: Baden-Württemberg; locality: Strohgäu, Markgröningen, EVS-Grundstück im Leudelsbachtal; verbatimElevation: 270 m; decimalLatitude: 48.9147; decimalLongitude: 9.0864; **Identification:** identifiedBy: H. Baur; dateIdentified: 2014; **Event:** samplingProtocol: Malaise trap; eventDate: 1/8 - 15/8/2011; **Record Level:** datasetID: SMNS_Hym_Pte_000790; institutionCode: SMNS

#### Description

##### Images

Fig. 4

##### Description of males

Colour: Head and mesosoma: mostly green, partly green-bluish to green bronze, with metallic lustre; setae on head and mesosoma: generally whitish, inconspicuous, some setae on top of head and lateral sides of anterior mesosoma fuscous to brown; tegula: yellow to testaceous; setae on callus of propodeum: whitish. Scape: uniformly yellow to slightly testaceous; pedicel: yellow to slightly testaceous; flagellum: fuscous. Forewing: hyaline; forewing venation: lightly testaceous; setae on forewing: fuscous; hind wing: hyaline. Procoxa and mesocoxa: infuscate, lighter on distal tip; metacoxa: brown with metallic tinge; trochanters: yellow; femora: uniformly yellow; tibiae: uniformly yellow; tarsae: uniformly yellow. Petiole: dark brown; gaster: gradient from brown to dark brown from anterior to posterior; gastral terga: without metallic tinge.

Sculpture: Head in frontal view: reticulate with moderately high septa; clypeus: striate; area between clypeus and malar sulcus: clypeal striation extending half distance, other half finely reticulate. Mesoscutum: finely reticulate, meshes moderately low, areoles slightly enlarged medially; mesoscutellum: finely engraved reticulation, finer mesh than on mesoscutum; frenum: finely engraved reticulation, mesh markedly larger than on mesoscutellum; axilla: finely engraved reticulation, about as strongly as on central part of mesoscutellum; prepectus upper triangular area: smooth; upper mesepimeron: smooth with hints of engraved reticulation; upper mesepisternum: reticulate, about as strongly as on mesoscutum; metapleuron: finely reticulate, less strongly than on mesepisternum. Procoxa: alutaceous; mesocoxa: alutaceous; metacoxa: alutaceous finely reticulate. Median area of propodeum: strongly, slightly irregularly reticulate, stronger than on mesoscutum, with few coarse longitudinal ridges; inner corner of anterior plica: with a large depression, flat surface smooth; nucha: strongly reticulate, equally in form and strength to median area of propodeum; callus of propodeum: coarsely irregularly reticulate; paraspiracular sulcus: fairly smooth directly around spiracle, posteriorly with irregular rugae. Petiole in dorsal view: smooth; gastral terga: smooth and shining, from second tergum posteriorly alutaceous.

Shape and structure: Head in frontal view: subtrapezoid with rounded vertex; gena in frontal view: rather straight; temple in dorsal view: obtuse; occipital carina: absent; torulus position with respect to lower ocular line: distinctly above; lower face in lateral view: rather rounded, with slight bulge on lower face below toruli; receding with respect to upper face: weakly; scrobe: narrow, moderately deep; malar sulcus: superficial, traceable only through change in sculpture; clypeus anterior margin: produced with straight anterior margin, slightly embedded in comparison to frontal pane of head, without a depression above emarginate edge; tentorial pit: indistinct; mouth extension: enlarged; mandibular formula: 3-not assessable. Antenna: Antennal formula: 11263; scape reaching: close to vertex; flagellum: filiform; first anellus: strongly transverse; second anellus: strongly transverse; first funicular segment: conical, quadrate, slightly tapering proximally; following funicular segements: conical, quadrate to slightly elongate, tapering less proximally than first segment;setae on flagellum: loosely clothed with forward-pointed erect setae, length of setae as long as breadth of flagellar segments; number of rows of longitudinal sensilla on first funicular segment: 1; on sixth: 1. Mesosoma in lateral view: moderately bent; propodeum in lateral view in same plane as mesoscutellum; pronotum breadth with respect to mesoscutum breadth: distinctly narrower; pronotum collar: slightly sloping upwards, well defined; its length with respect to mesoscutum length: slightly more than one sixth; its anterior margin: sharp carina throughout; pronotum posterior margin: shiny, finely reticulate towards lateral edges; notauli: superficial; reaching: two-thirds along mesoscutum; mesoscutellum in lateral view: almost flat, only slightly arched; mesoscutellum in posterior view: flat; mesoscutellum posterior margin projection: level of anterior margin of metascutellum; mesoscutellum posterior margin in posterior view: appearing ecarinate; frenal line: indicated through row of deeper areoles; prepectus upper triangular area: uniform, without separating carina; upper mesepimeron: almost parallel sides, only tapering at base of mesopleuron; propodeum anterior plica: strong, running along median area of propodeum; posterior plica: present, joining anterior plica; orientation of posterior plica: strongly converging in front of nucha, continuing almost parallel along its side; median carina of propodeum: present, but sometimes incomplete, may bifurcate from base of propodeum; nucha: large and convex, delimited from median area by shallow wide furrow; spiracle: oval, not nearly touching anterior margin of propodeum; size: moderate to small; callus pilosity: densely pilose; paraspiracular sulcus: hardly developed, indicated through smooth surface. Forewing: Forewing apex with respect to apex of gaster when folded back: exceeding close to 1/5th its length; basal cell number of setae: few up to 4 distally; basal setal line: patchy with 3-6 setae; cubital setal line: incomplete; with 5-7 setae; costal cell pilosity on dorsal side: bare; costal setal line: somewhat complete; speculum on dorsal side: bare, extending close to 1/3rd the length of the marginal vein, widely open below; wing disc: moderately pilose; marginal setae: present, from as long as the length of setae on wing disc on anterior wing margin to about double the length on posterior margin; stigma: oval to almost round, moderate size; uncus: short. Femora: moderately slender; metatibia: gradually widening towards apex; metacoxa pilosity dorsal: bare. Petiole in dorsal view: subconical, widening towards base of gaster; in ventral view: open; gaster in dorsal view: ovate, slightly acuminate; gastral terga: convex, sometimes weakly sunken; posterior margin of first gastral tergum: entire; first gastral tergum reaching: 1/3rd of gaster.

Measurements (n=2): Length and body ratios: Body length: 1.38—1.55 mm; mesoscutum breadth: 399—465 µm. Head breadth to head height: 1.40—1.41; head breadth to length: 2.07—2.19; head breadth to mesoscutum breadth: 1.21—1.29; upper face height to head height: 0.59; POL to OOL: 1.67—1.70; eye height to breadth: 1.37—1.39; eye distance to height: 1.30—1.36; temple length to eye height: 0.12—0.18; malar space to eye height: 0.32—0.37. Antenna length, including scape to head breadth: 1.43—1.48; scape length to eye height: 0.81—0.85; pedicel length to breadth: 1.68—1.85. Mesosoma length to mesoscutum breadth: 1.34—1.58; mesoscutum breadth to length: 1.85—2.03; mesoscutum length to mesoscutellum length: 1.02—1.03; propodeum length to mesoscutellum length: 0.74—0.90; plica distance to propodeum length: 1.21—1.45. Forewing length to breadth: 2.34—2.36; marginal vein to stigmal vein length: 2.01—2.05. Metafemur length to breadth: 4.73—4.75.

#### Molecular similarity to female in COI

mean p-distance to females: 0.00384, S.E.: 0.00232

## Checklists

### Ceinae Bouček, 1961

#### Spalangiopelta dudichi

Erdös, 1955

B1E06305-7F6A-5B19-87AA-339DDAD092A6

##### Materials

- **Type status:** Other material. **Occurrence:** recordedBy: T. Kothe, M. Engelhardt, C. König; sex: female; lifeStage: adult; preparations: dry mounted; **Taxon:** scientificName: *Spalangiopeltadudichi* Erdös, 1955; **Location:** continent: Europe; country: Germany; countryCode: DE; stateProvince: Baden-Württemberg; locality: Lkr. Tübingen, Steinenberg, Flurstücknummer 2906, MF 2; verbatimElevation: 471 m; decimalLatitude: 48.5306; decimalLongitude: 9.0312; **Identification:** identifiedBy: M. Moser; dateIdentified: 2021; **Event:** samplingProtocol: Malaise trap; eventDate: 4/7 - 17/7/2014; **Record Level:** datasetID: SMNS_Hym_Pte_010581; institutionCode: SMNS

##### Ecological interactions

###### Parasite of

The host of the species is unknown, but members of the genus are reported to be parasitoids of Diptera (Drosophilidae) associated with Brassicaceae (*Cakilemaritima* Scop.) in leaf litter habitats.

##### Distribution

Northern, eastern and southern Europe; Germany: Baden-Württemberg

##### Notes

Newly-recorded species in Germany. No Barcode available. Images: Fig. 5.

### Cleonyminae Walker, 1837

#### Cleonymus brevis

Bouček, 1972

3E8D8B1B-2CBC-5FC5-9B9D-BE61814CBA85

##### Materials

- **Type status:** Other material. **Occurrence:** recordedBy: L. Krogmann, T. Kothe; sex: female; lifeStage: adult; preparations: dry mounted; **Taxon:** scientificName: *Cleonymusbrevis* Bouček, 1972; **Location:** continent: Europe; country: Germany; countryCode: DE; stateProvince: Baden-Württemberg; locality: Strohgäu, Markgröningen, EVS-Grundstück im Leudelsbachtal; verbatimElevation: 270 m; decimalLatitude: 48.9147; decimalLongitude: 9.0864; **Identification:** identifiedBy: L. Krogmann; **Event:** samplingProtocol: Malaise trap; eventDate: 5/6 - 19/6/2011; **Record Level:** datasetID: SMNS_Hym_Pte_000026; institutionCode: SMNS
- **Type status:** Other material. **Occurrence:** recordedBy: L. Krogmann, T. Kothe; sex: female; lifeStage: adult; preparations: dry mounted; **Taxon:** scientificName: *Cleonymusbrevis* Bouček, 1972; **Location:** continent: Europe; country: Germany; countryCode: DE; stateProvince: Baden-Württemberg; locality: Strohgäu, Markgröningen, EVS-Grundstück im Leudelsbachtal; verbatimElevation: 270 m; decimalLatitude: 48.9147; decimalLongitude: 9.0864; **Identification:** identifiedBy: L. Krogmann; **Event:** samplingProtocol: Malaise trap; eventDate: 5/6 - 19/6/2011; **Record Level:** datasetID: SMNS_Hym_Pte_000128; institutionCode: SMNS
- **Type status:** Other material. **Occurrence:** recordedBy: L. Krogmann, T. Kothe; sex: female; lifeStage: adult; preparations: dry mounted; **Taxon:** scientificName: *Cleonymusbrevis* Bouček, 1972; **Location:** continent: Europe; country: Germany; countryCode: DE; stateProvince: Baden-Württemberg; locality: Strohgäu, Markgröningen, EVS-Grundstück im Leudelsbachtal; verbatimElevation: 270 m; decimalLatitude: 48.9147; decimalLongitude: 9.0864; **Identification:** identifiedBy: L. Krogmann; **Event:** samplingProtocol: Malaise trap; eventDate: 19/7 - 1/8/2011; **Record Level:** datasetID: SMNS_Hym_Pte_000645; institutionCode: SMNS
- **Type status:** Other material. **Occurrence:** recordedBy: T. Kothe, G. Schweizer; sex: female; lifeStage: adult; preparations: dry mounted; **Taxon:** scientificName: *Cleonymusbrevis* Bouček, 1972; **Location:** continent: Europe; country: Germany; countryCode: DE; stateProvince: Baden-Württemberg; locality: Lkr. Esslingen, Oberboihingen; verbatimElevation: 307 m; decimalLatitude: 48.6506; decimalLongitude: 9.3689; **Identification:** identifiedBy: L. Krogmann; **Event:** samplingProtocol: Malaise trap; eventDate: 12/8 - 26/8/2012; **Record Level:** datasetID: SMNS_Hym_Pte_001022; institutionCode: SMNS
- **Type status:** Other material. **Occurrence:** recordedBy: T. Kothe, G. Schweizer; sex: female; lifeStage: adult; preparations: dry mounted; **Taxon:** scientificName: *Cleonymusbrevis* Bouček, 1972; **Location:** continent: Europe; country: Germany; countryCode: DE; stateProvince: Baden-Württemberg; locality: Lkr. Esslingen, Oberboihingen; verbatimElevation: 307 m; decimalLatitude: 48.6506; decimalLongitude: 9.3689; **Identification:** identifiedBy: L. Krogmann; **Event:** samplingProtocol: Malaise trap; eventDate: 16/7 - 12/8/2012; **Record Level:** datasetID: SMNS_Hym_Pte_001541; institutionCode: SMNS
- **Type status:** Other material. **Occurrence:** recordedBy: Entomologischer Verein Krefeld 1905; sex: female; lifeStage: adult; preparations: dry mounted; **Taxon:** scientificName: *Cleonymusbrevis* Bouček, 1972; **Location:** continent: Europe; country: Germany; countryCode: DE; stateProvince: Baden-Württemberg; locality: Lkr. Zollernalb, Bisingen, NSG 4.228 Zollerhalde, Flst.-Nr. 2455/0; verbatimElevation: 589 m; decimalLatitude: 48.3218; decimalLongitude: 8.9552; **Identification:** identifiedBy: M. Haas; dateIdentified: 2021; **Event:** samplingProtocol: malaise trap; eventDate: 3/8 - 17/8/2021; **Record Level:** datasetID: SMNS_Hym_Pte_011035; institutionCode: SMNS

##### Ecological interactions

###### Parasite of

The species was reported as a parasitoid of wood-boring Coleoptera (Scolytidae).

##### Distribution

Europe except north; Germany: Baden Württemberg

##### Notes

Newly-recorded species in Germany. No Barcode available. Images: Fig. 6.

#### Cleonymus obscurus

Walker, 1837

0CB88B79-C0F1-5065-957B-FB4BFDD91076

##### Materials

- **Type status:** Other material. **Occurrence:** catalogNumber: BOLD Sample ID: SMNS_40088; recordedBy: B. Rulik; sex: male; lifeStage: adult; preparations: dry mounted; associatedSequences: GenBank: OL538152; **Taxon:** scientificName: *Cleonymusobscurus* Walker, 1837; **Location:** continent: Europe; country: Germany; countryCode: DE; stateProvince: Rheinland-Pfalz; locality: Kreis Ahrweiler, Niederzissen Bausenberg, Trockenrasen, MF5, ZFMK-MAL-0000719; verbatimElevation: 321 m; decimalLatitude: 50.4684; decimalLongitude: 7.2220; **Identification:** identifiedBy: M. Haas; dateIdentified: 2019; **Event:** samplingProtocol: Malaise trap; eventDate: 23/8 - 6/9/2012; **Record Level:** datasetID: SMNS_Hym_Pte_003023; institutionCode: SMNS
- **Type status:** Other material. **Occurrence:** catalogNumber: BOLD Sample ID: SMNS_40102; recordedBy: B. Rulik; sex: male; lifeStage: adult; preparations: dry mounted; associatedSequences: GenBank: OL538133; **Taxon:** scientificName: *Cleonymusobscurus* Walker, 1837; **Location:** continent: Europe; country: Germany; countryCode: DE; stateProvince: Rheinland-Pfalz; locality: Kreis Ahrweiler, Niederzissen, Bausenberg, Trockenrasen, MF5, ZFMK-MAL-0000567; verbatimElevation: 321 m; decimalLatitude: 50.4684; decimalLongitude: 7.2220; **Identification:** identifiedBy: M. Haas; dateIdentified: 2021; **Event:** samplingProtocol: Malaise trap; eventDate: 9/8 - 23/8/2012; **Record Level:** datasetID: SMNS_Hym_Pte_003037; institutionCode: SMNS
- **Type status:** Other material. **Occurrence:** catalogNumber: BOLD Sample ID: SMNS_40117; recordedBy: B. Rulik; sex: female; lifeStage: adult; preparations: dry mounted; associatedSequences: GenBank: OL538131; **Taxon:** scientificName: *Cleonymusobscurus* Walker, 1837; **Location:** continent: Europe; country: Germany; countryCode: DE; stateProvince: Rheinland-Pfalz; locality: Kreis Ahrweiler, Bausenberg, Eichen-Buchen-Mischwald, MF6, Niederzissen, ZFMK-MAL-0000091; verbatimElevation: 343 m; decimalLatitude: 50.4708; decimalLongitude: 7.2234; **Identification:** identifiedBy: M. Haas; dateIdentified: 2021; **Event:** samplingProtocol: Malaise trap; eventDate: 23/8 - 6/9/2012; **Record Level:** datasetID: SMNS_Hym_Pte_003052; institutionCode: SMNS
- **Type status:** Other material. **Occurrence:** catalogNumber: BOLD Sample ID: SMNS_40157; recordedBy: H.-J. Flügel; sex: female; lifeStage: adult; preparations: dry mounted; associatedSequences: GenBank: OL538073; **Taxon:** scientificName: *Cleonymusobscurus* Walker, 1837; **Location:** continent: Europe; country: Germany; countryCode: DE; stateProvince: Hessen; locality: Lkr. Schwalm-Eder-Kreis, Neumorschen, Halberg, 5283g; verbatimElevation: 196 m; decimalLatitude: 51.0628; decimalLongitude: 9.6022; **Identification:** identifiedBy: M. Haas; dateIdentified: 2019; **Event:** samplingProtocol: Malaise trap; eventDate: 1/7 - 19/7/2013; **Record Level:** datasetID: SMNS_Hym_Pte_003092; institutionCode: SMNS

##### Ecological interactions

###### Parasite of

The species was reported as a parasitoid of wood-boring Coleoptera (Scolytidae).

##### Distribution

Europe incl. United Kingdom; Germany: Hessen, Rheinland-Pfalz

##### Notes

Newly-recorded species in Germany. Tentative synonym to *Cleonymuslaticornis* Walker, 1837 (Bouček 1972), but female and male fit the characters described in Graham (1969). Images: Fig. 7.

### Miscogastrinae Walker, 1833

#### Halticoptera longipetiolus

Hedqvist, 1975

9947A7A2-5F31-501D-9C93-0F2348C0C683

##### Materials

- **Type status:** Other material. **Occurrence:** catalogNumber: BOLD Sample ID: SMNS_38302; recordedBy: D. Doczkal; sex: male; lifeStage: adult; preparations: dry mounted; associatedSequences: GenBank: OL538149; **Taxon:** scientificName: *Halticopteralongipetiolus* Hedqvist, 1975; **Location:** continent: Europe; country: Germany; countryCode: DE; stateProvince: Bayern; locality: Obergrashof 1, ss.ogh1.08; **Identification:** identifiedBy: H. Baur; dateIdentified: 2016; **Event:** samplingProtocol: Malaise trap; eventDate: 1/8/2011; **Record Level:** datasetID: SMNS_Hym_Pte_001239; institutionCode: SMNS
- **Type status:** Other material. **Occurrence:** catalogNumber: BOLD Sample ID: SMNS_38924; recordedBy: L. Krogmann, T. Kothe; sex: male; lifeStage: adult; preparations: dry mounted; associatedSequences: GenBank: OL538075; **Taxon:** scientificName: *Halticopteralongipetiolus* Hedqvist, 1975; **Location:** continent: Europe; country: Germany; countryCode: DE; stateProvince: Sachsen; locality: Lkr. Bautzen, Driewitz, (1); verbatimElevation: 146 m; decimalLatitude: 51.3456; decimalLongitude: 14.4315; **Identification:** identifiedBy: H. Baur; dateIdentified: 2016; **Event:** samplingProtocol: Sweep net; eventDate: 30/7/2013; **Record Level:** datasetID: SMNS_Hym_Pte_001861; institutionCode: SMNS

##### Ecological interactions

###### Parasite of

The species was reported as a parasitoid of leaf-mining Diptera (Agromyzidae) associated with Caryophylaceae.

##### Distribution

Sweden and Turkey; Germany: Bayern, Sachsen

##### Notes

Newly-recorded species in Germany. Images: Fig. 8.

#### Ksenoplata quadrata

Bouček, 1965

1BEAECEC-7BF2-5216-B012-15130B8FB77B

##### Materials

- **Type status:** Other material. **Occurrence:** catalogNumber: BOLD Sample ID: SMNS_38480; recordedBy: L. Krogmann, T. Kothe; sex: female; lifeStage: adult; preparations: dry mounted; associatedSequences: GenBank: OL538130; **Taxon:** scientificName: *Ksenoplataquadrata* Bouček, 1965; **Location:** continent: Europe; country: Germany; countryCode: DE; stateProvince: Sachsen; locality: Lkr. Bautzen, Baruth, Schafberg, Basaltwerk; verbatimElevation: 174 m; decimalLatitude: 51.2330; decimalLongitude: 14.6011; **Identification:** identifiedBy: M. Haas; dateIdentified: 2021; **Event:** samplingProtocol: Sweep net; eventDate: 31/7/2013; **Record Level:** datasetID: SMNS_Hym_Pte_001417; institutionCode: SMNS
- **Type status:** Other material. **Occurrence:** catalogNumber: BOLD Sample ID: SMNS_40455; recordedBy: I. Wendt; sex: female; lifeStage: adult; preparations: dry mounted; associatedSequences: GenBank: OL538087; **Taxon:** scientificName: *Ksenoplataquadrata* Bouček, 1965; **Location:** continent: Europe; country: Germany; countryCode: DE; stateProvince: Brandenburg; locality: Lkr. Uckermark, Lübbenow, Rasenrand; decimalLatitude: 53.4509; decimalLongitude: 13.8073; **Identification:** identifiedBy: M. Haas; dateIdentified: 2019; **Event:** samplingProtocol: Sweep net; eventDate: 19/6/2014; **Record Level:** datasetID: SMNS_Hym_Pte_003390; institutionCode: SMNS

##### Ecological interactions

###### Parasite of

The species was reported as a parasitoid of Coleoptera (Bruchidae: *Bruchidius* sp.), associated with Fabaceae (*Medicagopolymorpha* L., Syn.: *Medicagolappacea* Desr.).

##### Distribution

Mediterranean and eastern Europe; Germany: Brandenburg, Sachsen

##### Notes

Newly-recorded genus and species in Germany. Images: Fig. 9.

#### Rhicnocoelia impar

(Walker, 1836)

4E763882-B748-5985-81D2-C8D02D4B9B0E

##### Materials

- **Type status:** Other material. **Occurrence:** catalogNumber: BOLD Sample ID: SMNS_38987; recordedBy: T. Kothe; sex: female; lifeStage: adult; preparations: dry mounted; associatedSequences: GenBank: OL538055; **Taxon:** scientificName: *Rhicnocoeliaimpar* (Walker, 1836); **Location:** continent: Europe; country: Germany; countryCode: DE; stateProvince: Baden-Württemberg; locality: Lkr. Waldshut, Herrischried, Kreuzfeld; verbatimElevation: 998 m; decimalLatitude: 47.6572; decimalLongitude: 7.9662; **Identification:** identifiedBy: M. Haas; dateIdentified: 2021; **Event:** samplingProtocol: Sweep net; eventDate: 4/9/2013; **Record Level:** datasetID: SMNS_Hym_Pte_001924; institutionCode: SMNS
- **Type status:** Other material. **Occurrence:** catalogNumber: BOLD Sample ID: SMNS_40918; recordedBy: I. Wendt; sex: male; lifeStage: adult; preparations: dry mounted; associatedSequences: GenBank: OL538084; **Taxon:** scientificName: *Rhicnocoeliaimpar* (Walker, 1836); **Location:** continent: Europe; country: Germany; countryCode: DE; stateProvince: Hessen; locality: Wetteraukreis, Dorn-Assenheim, Wiese; decimalLatitude: 50.3468; decimalLongitude: 8.8492; **Identification:** identifiedBy: M. Haas; dateIdentified: 2021; **Event:** samplingProtocol: Sweep net; eventDate: 28/9/2014; **Record Level:** datasetID: SMNS_Hym_Pte_003853; institutionCode: SMNS

##### Ecological interactions

###### Parasite of

The host of the species is unknown, but probably a member of Diptera, associated with several plants: Asteraceae (*Onopordumnervosum* Boiss.), Euphorbiaceae (*Euphorbiaserrata* L.), Poaceae (*Triticum* sp.), Tamaricaceae (*Tamarixcanariensis* Willd.).

##### Distribution

Mediterranean, Europe incl. United Kingdom and Ireland, Canary Islands; Germany: Baden-Württemberg, Hessen

##### Notes

**Figure 3.**
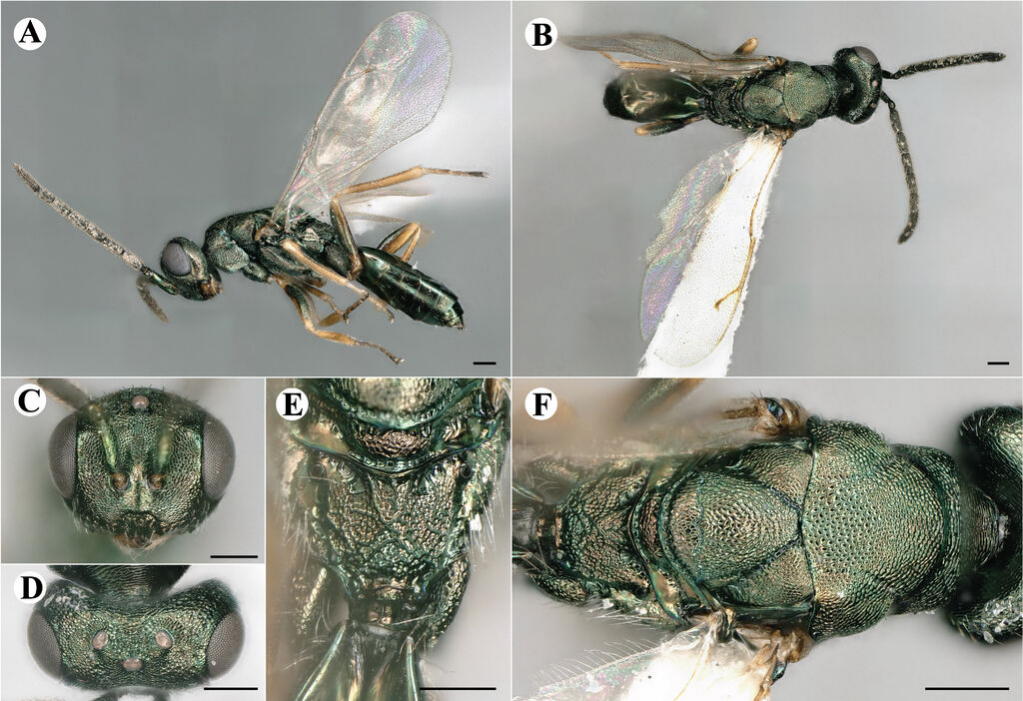
Digital microscopic images of the male *Rhicnocoeliaimpar* (Walker, 1836) (Pte_003853). **A** Habitus lateral **B** Habitus dorsal **C** Head frontal **D** Head dorsal **E** Metanotum and propodeum dorsal **F** Mesosoma dorsal. Scale bars: **A - B** 200 µm.

**Figure 5.**
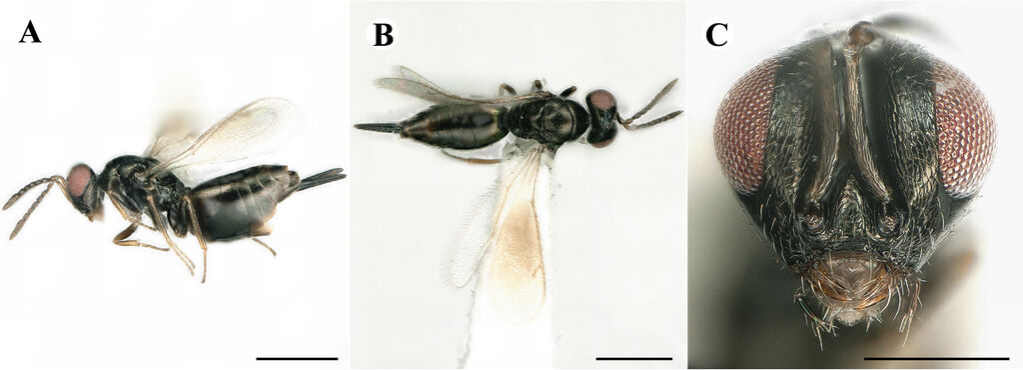
Digital microscopic images of *Spalangiopeltadudichi* Erdös, 1955 (♀, Pte_010581). **A** Habitus lateral **B** Habitus dorsal **C** Head frontal. Scale bars: **A, B** 500 µm; **C** 200 µm.

**Figure 6.**
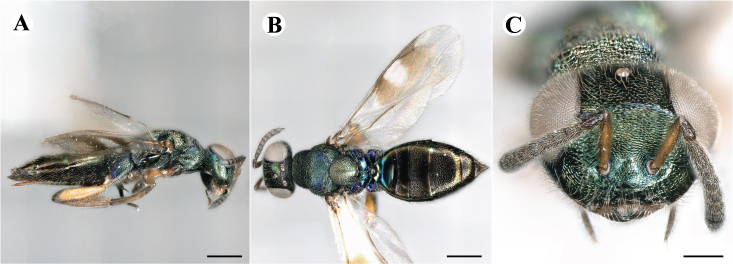
Digital microscopic images of *Cleonymusbrevis* Bouček, 1972 (♀, Pte_000645). **A** Habitus lateral **B** Habitus dorsal **C** Head frontal. Scale bars: **A, B** 500 µm; **C** 200 µm.

**Figure 7.**
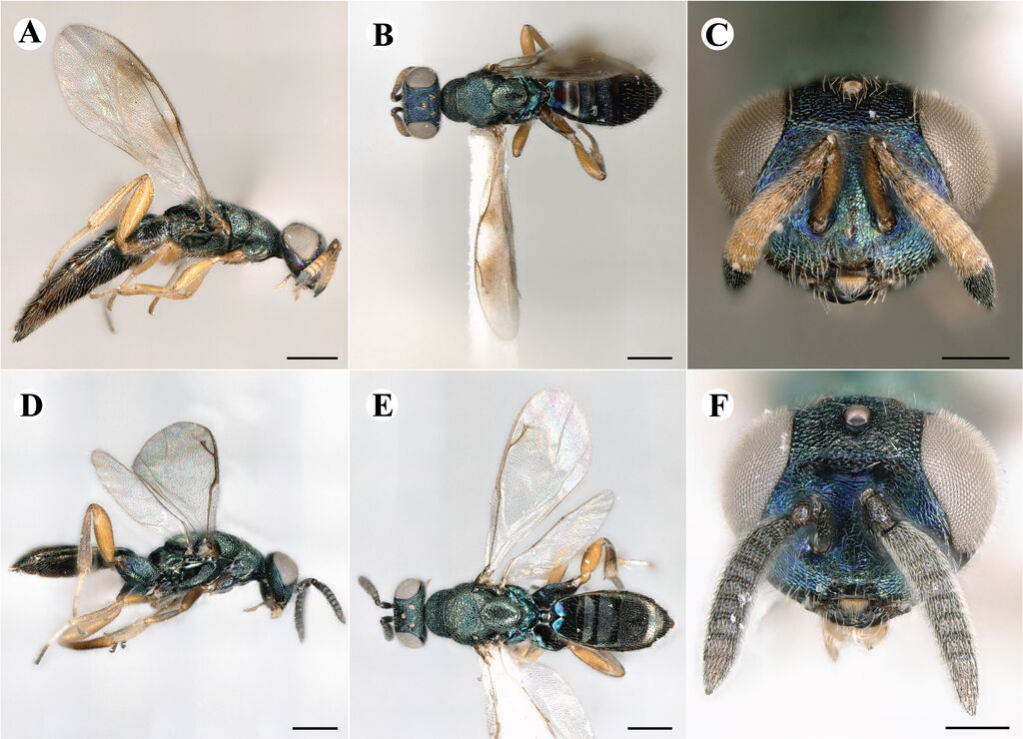
Digital microscopic images of *Cleonymusobscurus* Walker, 1837 (♀, Pte_003092: **A - C**; ♂, Pte_003023: **D - F**). **A, D** Habitus lateral **B, E** Habitus dorsal **C, F** Head frontal. Scale bars: **A, B, D, E** 500 µm; **C, F** 200 µm.

**Figure 8.**
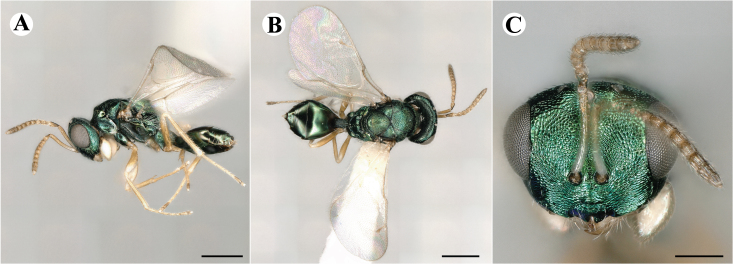
Digital microscopic images of *Halticopteralongipetiolus* Hedqvist, 1975 (♂, Pte_001239). **A** Habitus lateral **B** Habitus dorsal **C** Head frontal. Scale bars: **A, B** 500 µm; **C** 200 µm.

**Figure 9.**
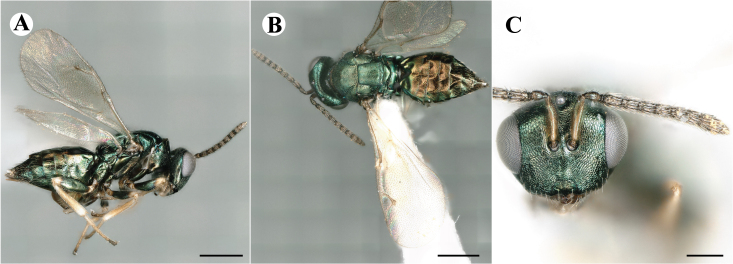
Digital microscopic images of *Ksenoplataquadrata* Bouček, 1965 (♀, Pte_001417). **A** Habitus lateral **B** Habitus dorsal **C** Head frontal. Scale bars: **A, B** 500 µm; **C** 200 µm.

**Figure 10.**
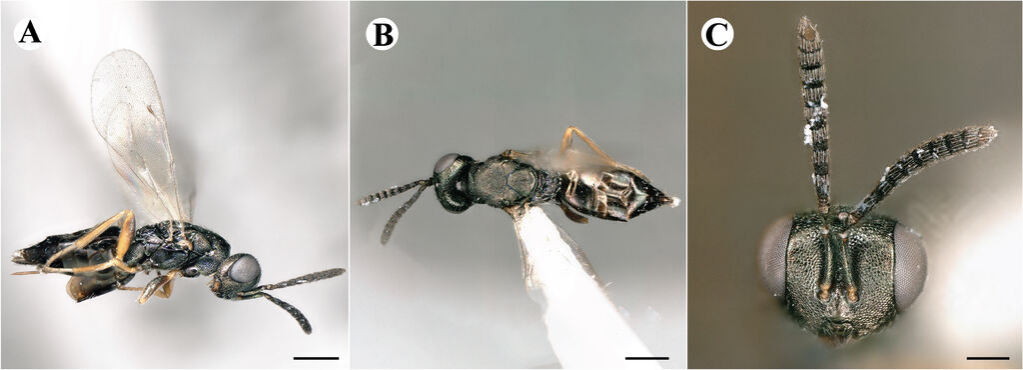
Digital microscopic images of *Rhicnocoeliaimpar* (Walker, 1836) (♀, Pte_001924). **A** Habitus lateral **B** Habitus dorsal **C** Head frontal. Scale bars: **A, B** 500 µm; **C** 200 µm.

Newly-recorded genus and species in Germany. According to Graham (1969), the identification of the two species *R.impar* and *R.constans* is quite difficult. *Rhicnocoeliaconstans* (Walker, 1836) has already been recorded from Germany. Graham’s characters for the wing venation do not work perfectly to distinguish our collected specimens, but it is clear that both species are morphologically different with regards to the metasomal size and overall colouration. Specimens of both species are also distinguishable, based on DNA barcoding, in our comparisons. A key to males is not available, but male specimens in our sampling could be unequivocally matched to their female counterpart by molecular evidence. Images: Figs 3, 10.

#### Tricyclomischus celticus

Graham, 1956

C7542DF1-6583-519A-ADF9-F3C96BD1B87A

##### Materials

- **Type status:** Other material. **Occurrence:** catalogNumber: BOLD Sample ID: SMNS_38109; recordedBy: L. Krogmann; sex: female; lifeStage: adult; preparations: dry mounted; associatedSequences: GenBank: OL538077; **Taxon:** scientificName: *Tricyclomischuscelticus* Graham, 1956; **Location:** continent: Europe; country: Germany; countryCode: DE; stateProvince: Baden-Württemberg; locality: Stuttgart, Universität Hohenheim; verbatimElevation: 350 m; decimalLatitude: 48.7072; decimalLongitude: 9.2147; **Identification:** identifiedBy: H. Baur; dateIdentified: 2014; **Event:** samplingProtocol: Sweep net, Malaise trap, yellow pan trap; eventDate: 15/6/2013; **Record Level:** datasetID: SMNS_Hym_Pte_001046; institutionCode: SMNS
- **Type status:** Other material. **Occurrence:** catalogNumber: BOLD Sample ID: SMNS_39101; recordedBy: H.-J. Flügel; sex: female; lifeStage: adult; preparations: dry mounted; associatedSequences: GenBank: OL538053; **Taxon:** scientificName: *Tricyclomischuscelticus* Graham, 1956; **Location:** continent: Europe; country: Germany; countryCode: DE; stateProvince: Hessen; locality: Lkr. Hersfeld-Rotenburg, Rockensüß, Doline, 5202b; verbatimElevation: 313 m; decimalLatitude: 51.0489; decimalLongitude: 9.8372; **Identification:** identifiedBy: M. Haas; dateIdentified: 2021; **Event:** samplingProtocol: Malaise trap; eventDate: 15/8 - 16/9/2013; **Record Level:** datasetID: SMNS_Hym_Pte_002038; institutionCode: SMNS
- **Type status:** Other material. **Occurrence:** catalogNumber: BOLD Sample ID: SMNS_50209; recordedBy: L. Krogmann, J. Holstein, T. Kothe; sex: female; lifeStage: adult; preparations: dry mounted; associatedSequences: GenBank: OL538082; **Taxon:** scientificName: *Tricyclomischuscelticus* Graham, 1956; **Location:** continent: Europe; country: Germany; countryCode: DE; stateProvince: Baden-Württemberg; locality: Lkr. Rems-Murr-Kreis, Aspach bei Backnang; verbatimElevation: 368.5 m; decimalLatitude: 48.9990; decimalLongitude: 9.4197; **Identification:** identifiedBy: M. Haas; dateIdentified: 2021; **Event:** samplingProtocol: Malaise trap; eventDate: 15/7 - 8/8/2013; **Record Level:** datasetID: SMNS_Hym_Pte_006660; institutionCode: SMNS

##### Ecological interactions

###### Parasite of

The host of the species is unknown, but is associated with Fabaceae (*Laburnumanagyroides* Medik.).

##### Distribution

Scattered eastern and northern Europe incl. United Kingdom; Germany: Baden-Württemberg, Hessen

##### Notes

**Figure 11.**
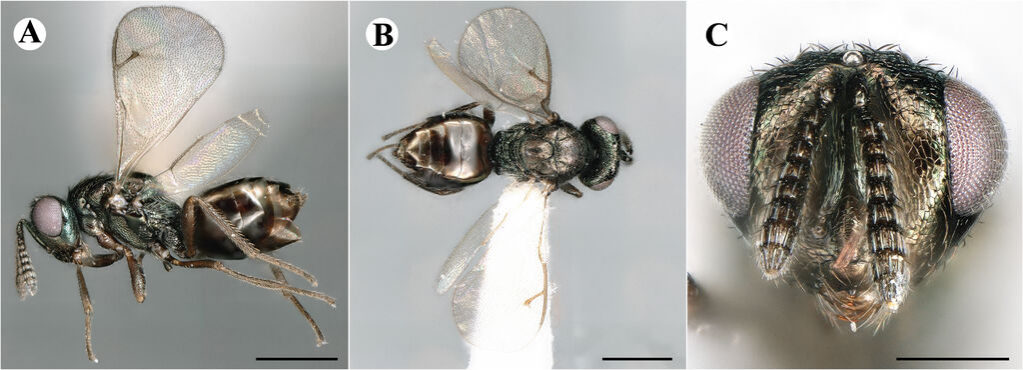
Digital microscopic images of *Tricyclomischuscelticus* Graham, 1956 (♀, Pte_001046). **A** Habitus lateral **B** Habitus dorsal **C** Head frontal. Scale bars: **A, B** 500 µm; **C** 200 µm.

Newly-recorded genus and species in Germany. Images: Fig. 11.

### Ormocerinae Walker, 1833

#### Systasis annulipes

(Walker, 1834)

9BE61C2A-ED83-5249-84C7-93DABBF4A10C

##### Materials

- **Type status:** Other material. **Occurrence:** catalogNumber: BOLD Sample ID: SMNS_40589; recordedBy: I. Wendt; sex: female; lifeStage: adult; preparations: dry mounted; associatedSequences: GenBank: OL538092; **Taxon:** scientificName: *Systasisannulipes* (Walker, 1834); **Location:** continent: Europe; country: Germany; countryCode: DE; stateProvince: Brandenburg; locality: Lkr. Uckermark, Lübbenow, Wegrand an Weizenfeld u. kleinem Wald; decimalLatitude: 53.4553; decimalLongitude: 13.8232; **Identification:** identifiedBy: M. Haas; dateIdentified: 2019; **Event:** samplingProtocol: Sweep net; eventDate: 20/6/2014; **Record Level:** datasetID: SMNS_Hym_Pte_003524; institutionCode: SMNS

##### Ecological interactions

###### Parasite of

The species was reported as a parasitoid of gall-inducing Hymenoptera Cynipidae (*Panteliellafedtschenkoi* (Rübsaamen, 1896)) associated with Lamiaceae (*Phlomoidestuberosa* Moench, Syn.: *Phlomistuberosa* L.).

##### Distribution

Northern and eastern Europe incl. United Kingdom; Germany: Brandenburg

##### Notes

**Figure 12.**
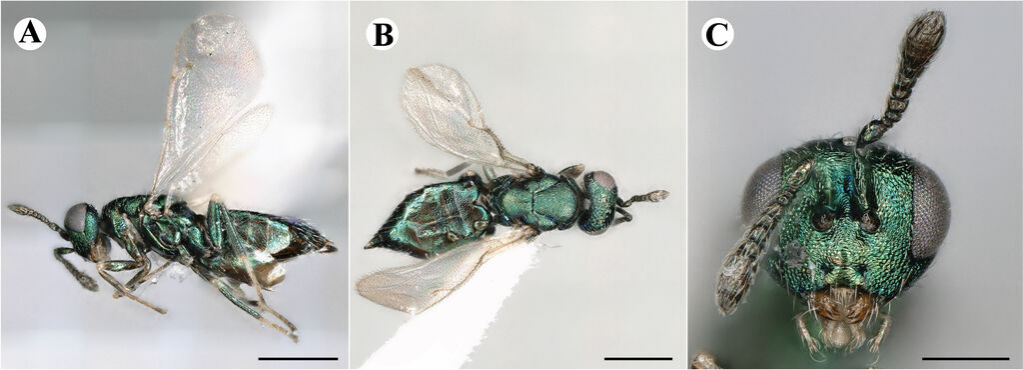
Digital microscopic images of *Systasisannulipes* (Walker, 1834) (♀, Pte_003524). **A** Habitus lateral **B** Habitus dorsal **C** Head frontal. Scale bars: **A, B** 500 µm; **C** 200 µm.

Newly-recorded species in Germany. Images: Fig. 12.

### Pireninae Haliday, 1844

#### Ecrizotes longicolis

(Walker, 1848)

7E2DC2F2-7CCF-531A-81C8-51D689459CD6

##### Materials

- **Type status:** Other material. **Occurrence:** catalogNumber: BOLD Sample ID: SMNS_38574; recordedBy: L. Krogmann, T. Kothe; sex: female; lifeStage: adult; preparations: dry mounted; associatedSequences: GenBank: OL538151; **Taxon:** scientificName: *Ecrizoteslongicornis* (Walker, 1848); **Location:** continent: Europe; country: Germany; countryCode: DE; stateProvince: Baden-Württemberg; locality: Lkr. Biberach, Federseegebiet; verbatimElevation: 579 m; decimalLatitude: 48.0640; decimalLongitude: 9.6378; **Identification:** identifiedBy: M. Haas; dateIdentified: 2016; **Event:** samplingProtocol: Sweep net; eventDate: 2/7/2013; **Record Level:** datasetID: SMNS_Hym_Pte_001511; institutionCode: SMNS

##### Ecological interactions

###### Parasite of

The host of the species is unknown, but is potentially associated with Betulaceae (*Betulla* sp.). Other members of the genus probably parasitise a host associated with grasses.

##### Distribution

Northern and eastern Europe incl. United Kingdom and Ireland; Germany: Baden-Württemberg

##### Notes

**Figure 13.**
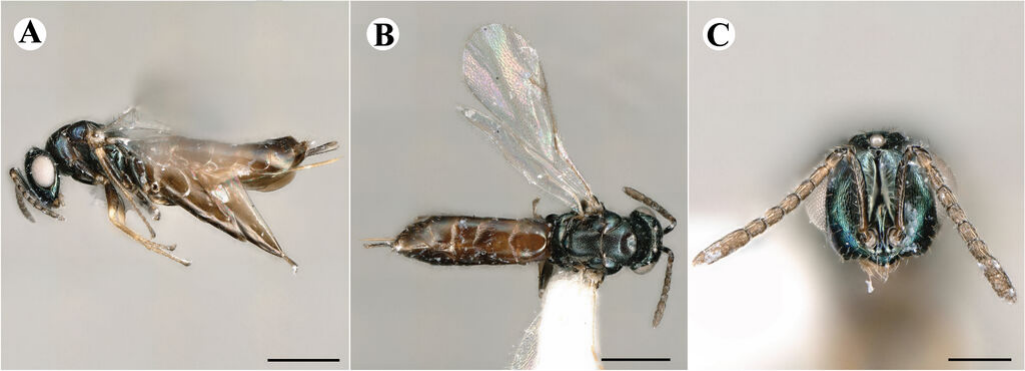
Digital microscopic images of *Ecrizoteslongicornis* (Walker, 1848) (♀, Pte_001511). **A** Habitus lateral **B** Habitus dorsal **C** Head frontal. Scale bars: **A, B** 500 µm; **C** 200 µm.

Newly-recorded genus and species in Germany. Images: Fig. 13.

#### Ecrizotes monticola

Foerster, 1861

CD96FA8F-20B2-543E-94F1-29BFDAF3C687

##### Materials

- **Type status:** Other material. **Occurrence:** catalogNumber: BOLD Sample ID: SMNS_39004; recordedBy: T. Kothe, C. König, J. Reibnitz; sex: female; lifeStage: adult; preparations: dry mounted; associatedSequences: GenBank: OL538125; **Taxon:** scientificName: *Ecrizotesmonticola* Foerster, 1861; **Location:** continent: Europe; country: Germany; countryCode: DE; stateProvince: Baden-Württemberg; locality: Lkr. Ostalbkreis, Tonnenberg; verbatimElevation: 544 m; decimalLatitude: 48.8701; decimalLongitude: 10.3166; **Identification:** identifiedBy: M. Haas; dateIdentified: 2016; **Event:** samplingProtocol: Sweep net; eventDate: 26/7/2013; **Record Level:** datasetID: SMNS_Hym_Pte_001941; institutionCode: SMNS

##### Ecological interactions

###### Parasite of

The host of the species is unknown . Other members of the genus probably parasitise a host associated with grasses and Betulaceae (*Betulla* sp.).

##### Distribution

Northern, central, eastern Europe incl. United Kingdom; Germany: Baden-Württemberg

##### Notes

**Figure 14.**
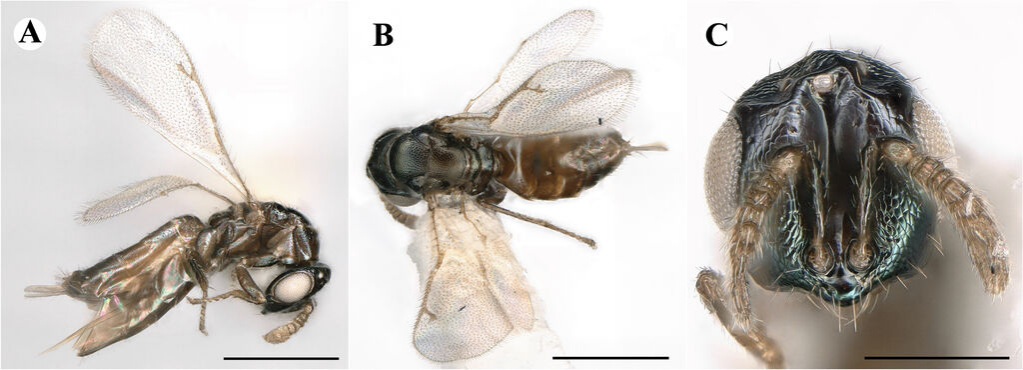
Digital microscopic images of *Ecrizotesmonticola* Foerster, 1861 (♀, Pte_001941). **A** Habitus lateral **B** Habitus dorsal **C** Head frontal. Scale bars: **A, B** 500 µm; **C** 200 µm.

Newly-recorded genus and species in Germany. Images: Fig. 14.

#### Gastrancistrus acutus

Graham, 1969

CDD85644-A099-50F0-8058-93CA810BB65E

##### Materials

- **Type status:** Other material. **Occurrence:** recordedBy: T. Kothe; sex: female; lifeStage: adult; preparations: dry mounted; associatedSequences: GenBank: OL538128; **Taxon:** scientificName: *Gastrancistrusacutus* Graham, 1969; **Location:** continent: Europe; country: Germany; countryCode: DE; stateProvince: Baden-Württemberg; locality: Lkr. Esslingen, Oberboihingen; verbatimElevation: 307 m; decimalLatitude: 48.6506; decimalLongitude: 9.3689; **Identification:** identifiedBy: M. Haas; dateIdentified: 2021; **Event:** samplingProtocol: Sweep net; eventDate: 7/6/2012; **Record Level:** language: BOLD Sample ID: SMNS_37959; datasetID: SMNS_Hym_Pte_000896; institutionCode: SMNS
- **Type status:** Other material. **Occurrence:** catalogNumber: BOLD Sample ID: SMNS_38384; recordedBy: L. Krogmann, T. Kothe, J. Reibnitz; sex: female; lifeStage: adult; preparations: dry mounted; associatedSequences: GenBank: OL538054; **Taxon:** scientificName: *Gastrancistrusacutus* Graham, 1969; **Location:** continent: Europe; country: Germany; countryCode: DE; stateProvince: Baden-Württemberg; locality: Alb-Donau-Kreis, NSG Kleines Lautertal; verbatimElevation: 627 m; decimalLatitude: 48.4538; decimalLongitude: 9.8412; **Identification:** identifiedBy: M. Haas; dateIdentified: 2021; **Event:** samplingProtocol: Sweep net; eventDate: 13/6/2013; **Record Level:** datasetID: SMNS_Hym_Pte_001321; institutionCode: SMNS

##### Ecological interactions

###### Parasite of

The host of the species is unknown, but members of the genus are reported to be largely parasitoids of Diptera (Cecidomyiidae).

##### Distribution

Europe incl. United Kingdom; Germany: Baden-Württemberg

##### Notes

**Figure 15.**
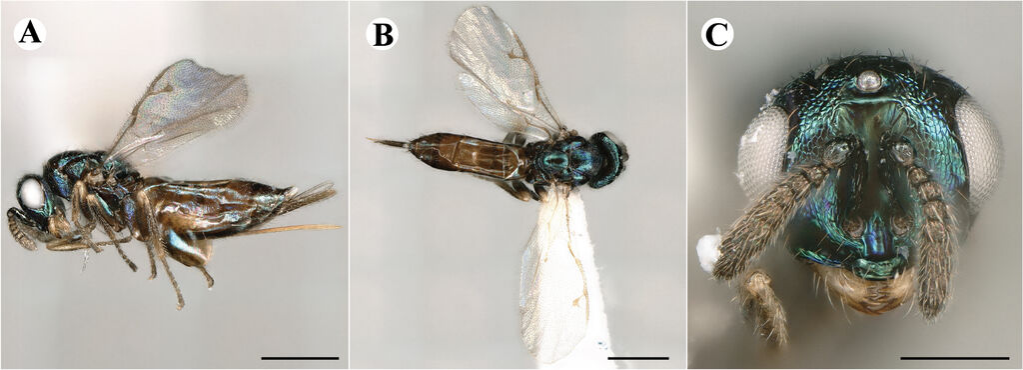
Digital microscopic images of *Gastrancistrusacutus* Graham, 1969 (♀, Pte_000896). **A** Habitus lateral **B** Habitus dorsal **C** Head frontal. Scale bars: **A, B** 500 µm; **C** 200 µm.

Newly-recorded species in Germany. Images: Fig. 15.

#### Gastrancistrus affinis

Graham, 1969

26754139-8F01-591F-A009-174A0B3E9445

##### Materials

- **Type status:** Other material. **Occurrence:** catalogNumber: BOLD Sample ID: SMNS_39975; recordedBy: I. Wendt; sex: female; lifeStage: adult; preparations: dry mounted; associatedSequences: GenBank: OL538098; **Taxon:** scientificName: *Gastrancistrusaffinis* Graham, 1969; **Location:** continent: Europe; country: Germany; countryCode: DE; stateProvince: Baden-Württemberg; locality: Lkr. Esslingen, Thomashardt, Waldrand; verbatimElevation: 434 m; decimalLatitude: 48.7514; decimalLongitude: 9.4795; **Identification:** identifiedBy: M. Haas; dateIdentified: 2019; **Event:** samplingProtocol: Malaise trap; eventDate: 26/3 - 9/4/2014; **Record Level:** datasetID: SMNS_Hym_Pte_002910; institutionCode: SMNS

##### Ecological interactions

###### Parasite of

The species was reported as a parasitoid of leaf-mining Diptera (Agromyzidae), associated with Fabaceae.

##### Distribution

Scattered northern, central and eastern Europe incl. United Kingdom; Germany: Baden-Württemberg

##### Notes

**Figure 16.**
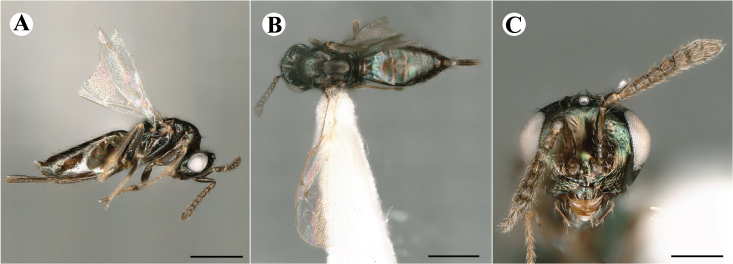
Digital microscopic images of *Gastrancistrusaffinis* Graham, 1969 (♀, Pte_002910). **A** Habitus lateral **B** Habitus dorsal **C** Head frontal. Scale bars: **A, B** 500 µm; **C** 200 µm.

Newly-recorded species in Germany. Images: Fig. 16.

#### Gastrancistrus compressus

Walker, 1834

0F1B4340-75BD-5565-8F35-99DBBFE5C7B1

##### Materials

- **Type status:** Other material. **Occurrence:** catalogNumber: BOLD Sample ID: SMNS_37951; recordedBy: T. Kothe; sex: male; lifeStage: adult; preparations: dry mounted; associatedSequences: GenBank: OL538107; **Taxon:** scientificName: *Gastrancistruscompressus* Walker, 1834; **Location:** continent: Europe; country: Germany; countryCode: DE; stateProvince: Baden-Württemberg; locality: Lkr. Esslingen, Oberboihingen; verbatimElevation: 307 m; decimalLatitude: 48.6506; decimalLongitude: 9.3689; **Identification:** identifiedBy: M. Haas; dateIdentified: 2021; **Event:** samplingProtocol: Sweep net; eventDate: 7/6/2012; **Record Level:** datasetID: SMNS_Hym_Pte_000888; institutionCode: SMNS
- **Type status:** Other material. **Occurrence:** catalogNumber: BOLD Sample ID: SMNS_38754; recordedBy: L. Krogmann, T. Kothe; sex: male; lifeStage: adult; preparations: dry mounted; associatedSequences: GenBank: OL538086; **Taxon:** scientificName: *Gastrancistruscompressus* Walker, 1834; **Location:** continent: Europe; country: Germany; countryCode: DE; stateProvince: Baden-Württemberg; locality: Lkr. Breisgau-Hochschwarzwald, NSG Badberg; verbatimElevation: 426 m; decimalLatitude: 48.0964; decimalLongitude: 7.6808; **Identification:** identifiedBy: M. Haas; dateIdentified: 2021; **Event:** samplingProtocol: Sweep net; eventDate: 18/6/2013; **Record Level:** datasetID: SMNS_Hym_Pte_001691; institutionCode: SMNS
- **Type status:** Other material. **Occurrence:** catalogNumber: BOLD Sample ID: SMNS_39280; recordedBy: L. Krogmann, T. Kothe; sex: male; lifeStage: adult; preparations: dry mounted; associatedSequences: GenBank: OL538064; **Taxon:** scientificName: *Gastrancistruscompressus* Walker, 1834; **Location:** continent: Europe; country: Germany; countryCode: DE; stateProvince: Baden-Württemberg; locality: Lkr. Main-Tauber-Kreis, Tauberbischofsheim, ex Truppenübungsplatz; verbatimElevation: 331 m; decimalLatitude: 49.6245; decimalLongitude: 9.7081; **Identification:** identifiedBy: M. Haas; dateIdentified: 2021; **Event:** samplingProtocol: Sweep net; eventDate: 12/6/2013; **Record Level:** datasetID: SMNS_Hym_Pte_002217; institutionCode: SMNS
- **Type status:** Other material. **Occurrence:** recordedBy: L. Krogmann, T. Kothe; sex: female; lifeStage: adult; preparations: dry mounted; **Taxon:** scientificName: *Gastrancistruscompressus* Walker, 1834; **Location:** continent: Europe; country: Germany; countryCode: DE; stateProvince: Baden-Württemberg; locality: Lkr. Breisgau-Hochschwarzwald, NSG Badberg; verbatimElevation: 426 m; decimalLatitude: 48.0964; decimalLongitude: 7.6808; **Identification:** identifiedBy: L. Krogmann; dateIdentified: 2015; **Event:** samplingProtocol: Sweep net; eventDate: 17/6 - 18/6/2013; **Record Level:** datasetID: SMNS_Hym_Pte_003750; institutionCode: SMNS
- **Type status:** Other material. **Occurrence:** recordedBy: L. Krogmann, T. Kothe; sex: female; lifeStage: adult; preparations: dry mounted; **Taxon:** scientificName: *Gastrancistruscompressus* Walker, 1834; **Location:** continent: Europe; country: Germany; countryCode: DE; stateProvince: Baden-Württemberg; locality: Lkr. Breisgau-Hochschwarzwald, NSG Badberg; verbatimElevation: 426 m; decimalLatitude: 48.0964; decimalLongitude: 7.6808; **Identification:** identifiedBy: L. Krogmann; dateIdentified: 2015; **Event:** samplingProtocol: Sweep net; eventDate: 17/6 - 18/6/2013; **Record Level:** datasetID: SMNS_Hym_Pte_003751; institutionCode: SMNS
- **Type status:** Other material. **Occurrence:** catalogNumber: BOLD Sample ID: SMNS_49989; recordedBy: L. Krogmann, T. Kothe; sex: female; lifeStage: adult; preparations: dry mounted; associatedSequences: GenBank: OL538093; **Taxon:** scientificName: *Gastrancistruscompressus* Walker, 1834; **Location:** continent: Europe; country: Germany; countryCode: DE; stateProvince: Baden-Württemberg; locality: Lkr. Breisgau-Hochschwarzwald, NSG Badberg; verbatimElevation: 426 m; decimalLatitude: 48.0964; decimalLongitude: 7.6808; **Identification:** identifiedBy: L. Krogmann; dateIdentified: 2015; **Event:** samplingProtocol: Sweep net; eventDate: 18/6/2013; **Record Level:** datasetID: SMNS_Hym_Pte_006440; institutionCode: SMNS

##### Ecological interactions

###### Parasite of

The species was reported as a parasitoid of gall-inducing Diptera (Cecidomyidae).

##### Distribution

Scattered northern, central and eastern Europe incl. United Kingdom; Germany: Baden-Württemberg

##### Notes

**Figure 17.**
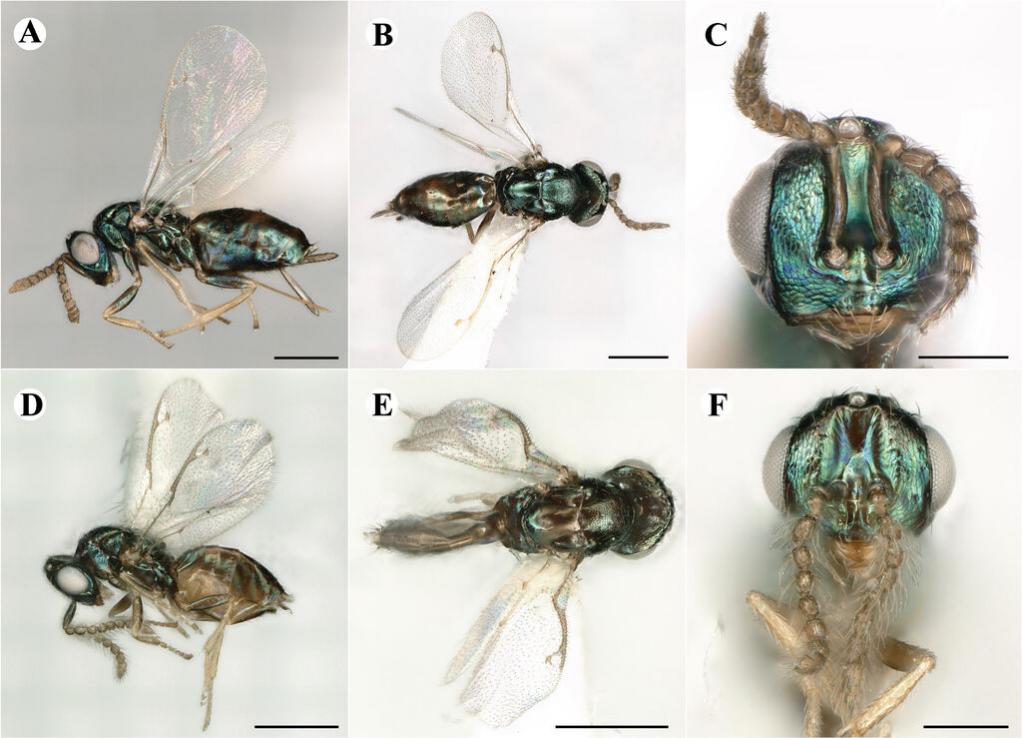
Digital microscopic images of *Gastrancistruscompressus* Walker, 1834 (♀ Pte_006440: **A - C**; ♂, Pte_000888: **D - F**). **A, D** Habitus lateral **B, E** Habitus dorsal **C, F** Head frontal. Scale bars: **A, B, D, E** 500 µm; **C, F** 200 µm.

Newly-recorded species in Germany. Images: Fig. 17.

#### Gastrancistrus fumipennis

Walker, 1834

2637A76C-11A8-5E22-92E4-6EDD6AE02179

##### Materials

- **Type status:** Other material. **Occurrence:** catalogNumber: BOLD Sample ID: SMNS_39174; recordedBy: H.-J. Flügel; sex: female; lifeStage: adult; preparations: dry mounted; associatedSequences: GenBank: OL538071; **Taxon:** scientificName: *Gastrancistrusfumipennis* Walker, 1834; **Location:** continent: Europe; country: Germany; countryCode: DE; stateProvince: Hessen; locality: Lkr. Schwalm-Eder-Kreis, Neumorschen, Halberg, 5267g; verbatimElevation: 196 m; decimalLatitude: 51.0628; decimalLongitude: 9.6022; **Identification:** identifiedBy: M. Haas; dateIdentified: 2015; **Event:** samplingProtocol: Malaise trap; eventDate: 1/7/2013; **Record Level:** datasetID: SMNS_Hym_Pte_002111; institutionCode: SMNS

##### Ecological interactions

###### Parasite of

The host of the species is unknown, but members of the genus are reported to be largely parasitoids of Diptera (Cecidomyidae).

##### Distribution

United Kingdom; Germany: Hessen

##### Notes

**Figure 18.**
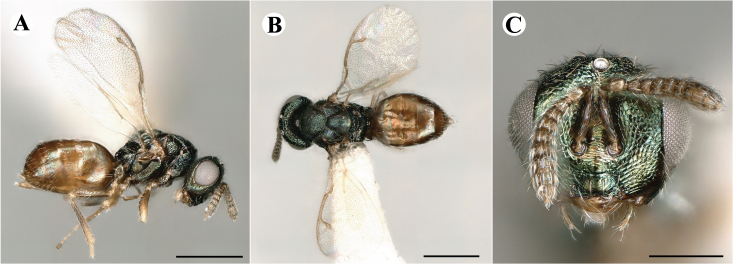
Digital microscopic images of *Gastrancistrusfumipennis* Walker, 1834 (♀, Pte_002111). **A** Habitus lateral **B** Habitus dorsal **C** Head frontal. Scale bars: **A, B** 500 µm; **C** 200 µm.

Newly-recorded species in Germany. Images: Fig. 18.

#### Macroglenes eximius

(Haliday, 1833)

D0998A32-A616-5534-A44E-8E11DA79E6D6

##### Materials

- **Type status:** Other material. **Occurrence:** catalogNumber: BOLD Sample ID: SMNS_38801; recordedBy: L. Krogmann, T. Kothe; sex: male; lifeStage: adult; preparations: dry mounted; associatedSequences: GenBank: OL538080; **Taxon:** scientificName: *Macrogleneseximius* (Haliday, 1833); **Location:** continent: Europe; country: Germany; countryCode: DE; stateProvince: Sachsen; locality: Lkr. Bautzen, Knappenrode; verbatimElevation: 125 m; decimalLatitude: 51.4008; decimalLongitude: 14.3279; **Identification:** identifiedBy: M. Haas; dateIdentified: 2019; **Event:** samplingProtocol: Sweep net; eventDate: 1/8/2013; **Record Level:** datasetID: SMNS_Hym_Pte_001738; institutionCode: SMNS

##### Ecological interactions

###### Parasite of

The species was reported as a parasitoid of gall-inducing Diptera (Cecidomyiidae: *Contarinianasturtii* (Kieffer, 1888), associated with Asteraceae (*Artemisiaherba-alba* Asso), contrary to other *Macroglenes* spp. being associated with grasses.

##### Distribution

Europe incl. United Kingdom and Ireland; Germany: Sachsen

##### Notes

**Figure 19.**
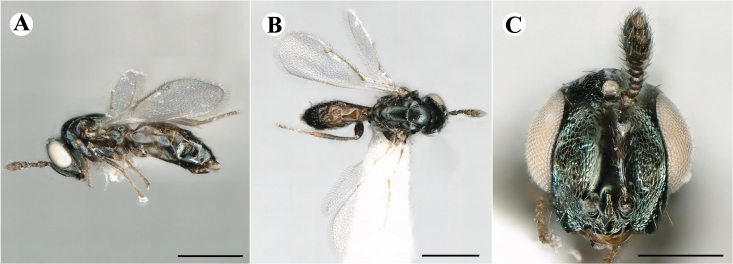
Digital microscopic images of *Macrogleneseximius* (Haliday, 1833) (♂, Pte_001738). **A** Habitus lateral **B** Habitus dorsal **C** Head frontal. Scale bars: **A, B** 500 µm; **C** 200 µm.

Newly-recorded species in Germany. Images: Fig. 19.

#### Macroglenes paludum

(Graham, 1969)

B36DEC8E-C6B8-54E9-8F0A-23B4F919B134

##### Materials

- **Type status:** Other material. **Occurrence:** catalogNumber: BOLD Sample ID: SMNS_39794; recordedBy: L. Krogmann, T. Kothe; sex: male; lifeStage: adult; preparations: dry mounted; associatedSequences: GenBank: OL538121; **Taxon:** scientificName: *Macroglenespaludum* (Graham, 1969); **Location:** continent: Europe; country: Germany; countryCode: DE; stateProvince: Baden-Württemberg; locality: Lkr. Biberach, Steinhauser Ried, 1); verbatimElevation: 584 m; decimalLatitude: 48.0418; decimalLongitude: 9.6461; **Identification:** identifiedBy: M. Haas; dateIdentified: 2015; **Event:** samplingProtocol: Sweep net; eventDate: 2/7/2013; **Record Level:** datasetID: SMNS_Hym_Pte_002729; institutionCode: SMNS
- **Type status:** Other material. **Occurrence:** catalogNumber: BOLD Sample ID: SMNS_284917; recordedBy: S. Bigalk; sex: male; lifeStage: adult; preparations: dry mounted; associatedSequences: GenBank: OL538136; **Taxon:** scientificName: *Macroglenespaludum* (Graham, 1969); **Location:** continent: Europe; country: Germany; countryCode: DE; stateProvince: Niedersachsen; locality: Land Hadeln, Otterndorf; verbatimElevation: -2 m; decimalLatitude: 53.8231; decimalLongitude: 8.8695; **Identification:** identifiedBy: M. Haas; dateIdentified: 2021; **Event:** samplingProtocol: Sweep net; eventDate: 6/6/2016; **Record Level:** datasetID: SMNS_Hym_Pte_010223; institutionCode: SMNS
- **Type status:** Other material. **Occurrence:** catalogNumber: BOLD Sample ID: SMNS_284919; recordedBy: S. Bigalk; sex: male; lifeStage: adult; preparations: dry mounted; associatedSequences: GenBank: OL538091; **Taxon:** scientificName: *Macroglenespaludum* (Graham, 1969); **Location:** continent: Europe; country: Germany; countryCode: DE; stateProvince: Niedersachsen; locality: Land Hadeln, Otterndorf; verbatimElevation: -2 m; decimalLatitude: 53.8231; decimalLongitude: 8.8695; **Identification:** identifiedBy: M. Haas; dateIdentified: 2021; **Event:** samplingProtocol: Sweep net; eventDate: 6/6/2016; **Record Level:** datasetID: SMNS_Hym_Pte_010225; institutionCode: SMNS

##### Ecological interactions

###### Parasite of

The species was reported as a parasitoid of gall-inducing Diptera (Cecidomyiidae: *Mayetiolaphalaris* Barnes, 1927), associated with Poaceae (*Phalarisarundinaceae* L.).

##### Distribution

Europe incl. United Kingdom and Ireland; Germany: Baden-Württemberg, Niedersachsen

##### Notes

**Figure 20.**
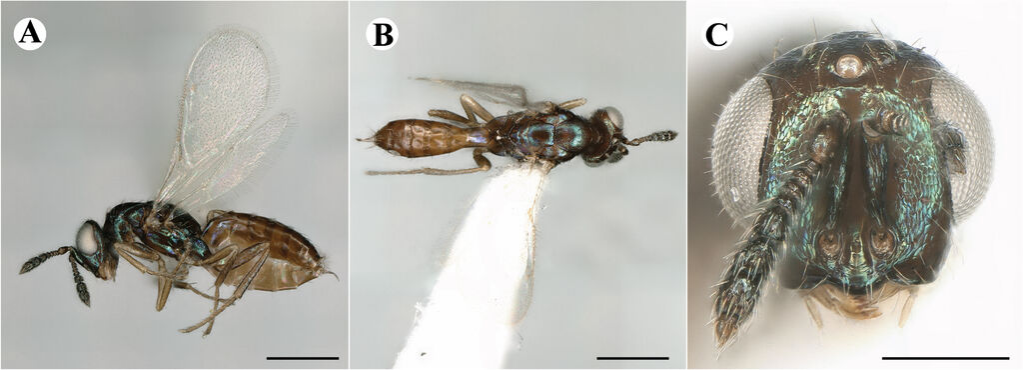
Digital microscopic images of *Macroglenespaludum* (Graham, 1969) (♂, Pte_010233). **A** Habitus lateral **B** Habitus dorsal **C** Head frontal. Scale bars: **A, B** 500 µm; **C** 200 µm.

Newly-recorded species in Germany. Images: Fig. 20.

#### Micradelus acutus

Graham, 1969

8CB04C9D-59C4-5E30-BCDE-B80B5901D820

##### Materials

- **Type status:** Other material. **Occurrence:** catalogNumber: BOLD Sample ID: SMNS_38412; recordedBy: D. Doczkal; sex: female; lifeStage: adult; preparations: dry mounted; associatedSequences: GenBank: OL538097; **Taxon:** scientificName: *Micradelusacutus* Graham, 1969; **Location:** continent: Europe; country: Germany; countryCode: DE; stateProvince: Bayern; locality: Obergrashof 3, ss.ogh3.01; **Identification:** identifiedBy: M. Haas; dateIdentified: 2015; **Event:** samplingProtocol: Malaise trap; eventDate: 21/4/2011; **Record Level:** datasetID: SMNS_Hym_Pte_001349; institutionCode: SMNS

##### Ecological interactions

###### Parasite of

The host of the species is unknown, but members of the genus are reported to be hyperparasitoids of Pteromalidae (Asaphinae: *Asaphesvulgaris* Walker, 1834).

##### Distribution

Europe incl. United Kingdom; Germany: Bayern

##### Notes

**Figure 21.**
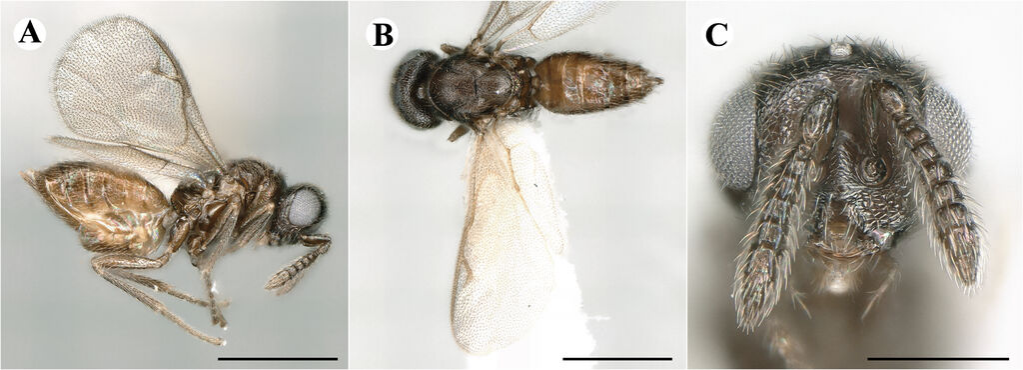
Digital microscopic images of *Micradelusacutus* Graham, 1969 (♀, Pte_001349). **A** Habitus lateral **B** Habitus dorsal **C** Head frontal. Scale bars: **A, B** 500 µm; **C** 200 µm.

Newly-recorded genus and species in Germany. Images: Fig. 21.

### Pteromalinae Dalman, 1820

#### Acrocormus semifasciatus

Thomson, 1878

E02A1CE6-ADBA-59D1-8711-AC544C421B9B

##### Materials

- **Type status:** Other material. **Occurrence:** catalogNumber: BOLD Sample ID: SMNS_37694; recordedBy: L. Krogmann, T. Kothe; sex: female; lifeStage: adult; preparations: dry mounted; associatedSequences: GenBank: OL538120; **Taxon:** scientificName: *Acrocormussemifasciatus* Thomson, 1878; **Location:** continent: Europe; country: Germany; countryCode: DE; stateProvince: Baden-Württemberg; locality: Strohgäu, Markgröningen, EVS-Grundstück im Leudelsbachtal; verbatimElevation: 270 m; decimalLatitude: 48.9147; decimalLongitude: 9.0864; **Identification:** identifiedBy: L. Krogmann; **Event:** samplingProtocol: Malaise trap; eventDate: 19/7 - 1/8/2011; **Record Level:** datasetID: SMNS_Hym_Pte_000631; institutionCode: SMNS

##### Ecological interactions

###### Parasite of

The species was reported as a parasitoid of wood-boring Coleoptera (Curculionidae and Scolytidae) in twigs of Fagaceae, Oleaceae and Ulmaceae.

##### Distribution

Central Asia to western Europe incl. United Kingdom; Germany: Baden-Württemberg

##### Notes

**Figure 22.**
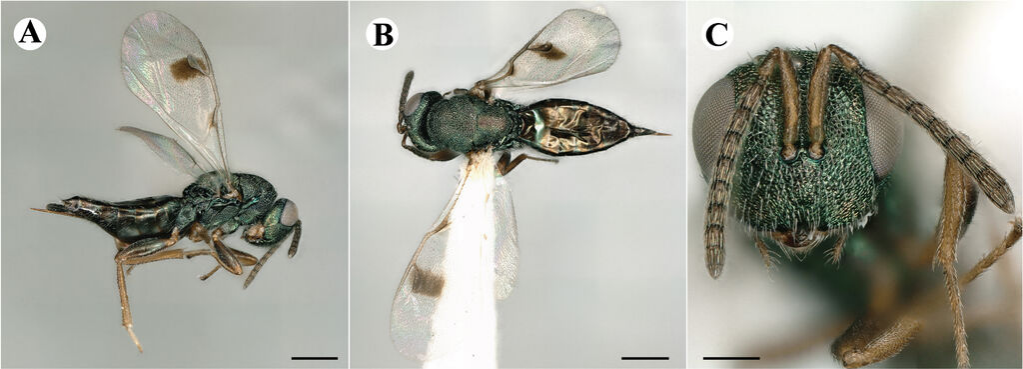
Digital microscopic images of *Acrocormussemifasciatus* Thomson, 1878 (♀, Pte_000631). **A** Habitus lateral **B** Habitus dorsal **C** Head frontal. Scale bars: **A, B** 500 µm; **C** 200 µm.

Newly-recorded genus and species in Germany. Images: Fig. 22.

#### Apelioma pteromalinum

(Thomson, 1878)

5DC1791B-C303-5203-8C22-98FA7BF4990E

##### Materials

- **Type status:** Other material. **Occurrence:** catalogNumber: BOLD Sample ID: SMNS_37573; recordedBy: H.-J. Flügel; sex: female; lifeStage: adult; preparations: dry mounted; associatedSequences: GenBank: OL538094; **Taxon:** scientificName: *Apeliomapteromalinum* (Thomson, 1878); **Location:** continent: Europe; country: Germany; countryCode: DE; stateProvince: Hessen; locality: Lkr. Schwalm-Eder-Kreis, Neumorschen, Halberg, 5202g; verbatimElevation: 196 m; decimalLatitude: 51.0628; decimalLongitude: 9.6022; **Identification:** identifiedBy: H. Baur; dateIdentified: 2014; **Event:** eventDate: 16/10/2012; **Record Level:** datasetID: SMNS_Hym_Pte_000510; institutionCode: SMNS
- **Type status:** Other material. **Occurrence:** catalogNumber: BOLD Sample ID: SMNS_39910; recordedBy: T. Kothe, G. Schweizer; sex: male; lifeStage: adult; preparations: dry mounted; associatedSequences: GenBank: OL538141; **Taxon:** scientificName: *Apeliomapteromalinum* (Thomson, 1878); **Location:** continent: Europe; country: Germany; countryCode: DE; stateProvince: Baden-Württemberg; locality: Lkr. Esslingen, Oberboihingen; verbatimElevation: 307 m; decimalLatitude: 48.6506; decimalLongitude: 9.3689; **Identification:** identifiedBy: M. Haas; dateIdentified: 2021; **Event:** samplingProtocol: Malaise trap; eventDate: 4/4 - 4/5/2014; **Record Level:** datasetID: SMNS_Hym_Pte_002845; institutionCode: SMNS
- **Type status:** Other material. **Occurrence:** catalogNumber: BOLD Sample ID: SMNS_39918; recordedBy: T. Kothe, G. Schweizer; sex: female; lifeStage: adult; preparations: dry mounted; associatedSequences: GenBank: OL538076; **Taxon:** scientificName: *Apeliomapteromalinum* (Thomson, 1878); **Location:** continent: Europe; country: Germany; countryCode: DE; stateProvince: Baden-Württemberg; locality: Lkr. Esslingen, Oberboihingen; verbatimElevation: 307 m; decimalLatitude: 48.6506; decimalLongitude: 9.3689; **Identification:** identifiedBy: M. Haas; dateIdentified: 2021; **Event:** samplingProtocol: Malaise trap; eventDate: 4/4 - 4/5/2014; **Record Level:** datasetID: SMNS_Hym_Pte_002853; institutionCode: SMNS
- **Type status:** Other material. **Occurrence:** catalogNumber: BOLD Sample ID: SMNS_40869; recordedBy: J. Aronov; sex: female; lifeStage: adult; preparations: dry mounted; associatedSequences: GenBank: OL538074; **Taxon:** scientificName: *Apeliomapteromalinum* (Thomson, 1878); **Location:** continent: Europe; country: Germany; countryCode: DE; stateProvince: Baden-Württemberg; locality: Stuttgart, Wilhelma, Hutewald, alt; verbatimElevation: 258 m; decimalLatitude: 48.8066; decimalLongitude: 9.1995; **Identification:** identifiedBy: M. Haas; dateIdentified: 2021; **Event:** samplingProtocol: Malaise trap; eventDate: 16/6 - 1/7/2014; **Record Level:** datasetID: SMNS_Hym_Pte_003804; institutionCode: SMNS

##### Ecological interactions

###### Parasite of

The species was reported as a parasitoid of wood-boring Coleoptera (Buprestidae: *Melanophilacyanea* (Fabricius, 1775)) in dying twigs.

##### Distribution

Eastern to central Europe incl. United Kingdom; Germany: Baden-Württemberg, Hessen

##### Notes

**Figure 23.**
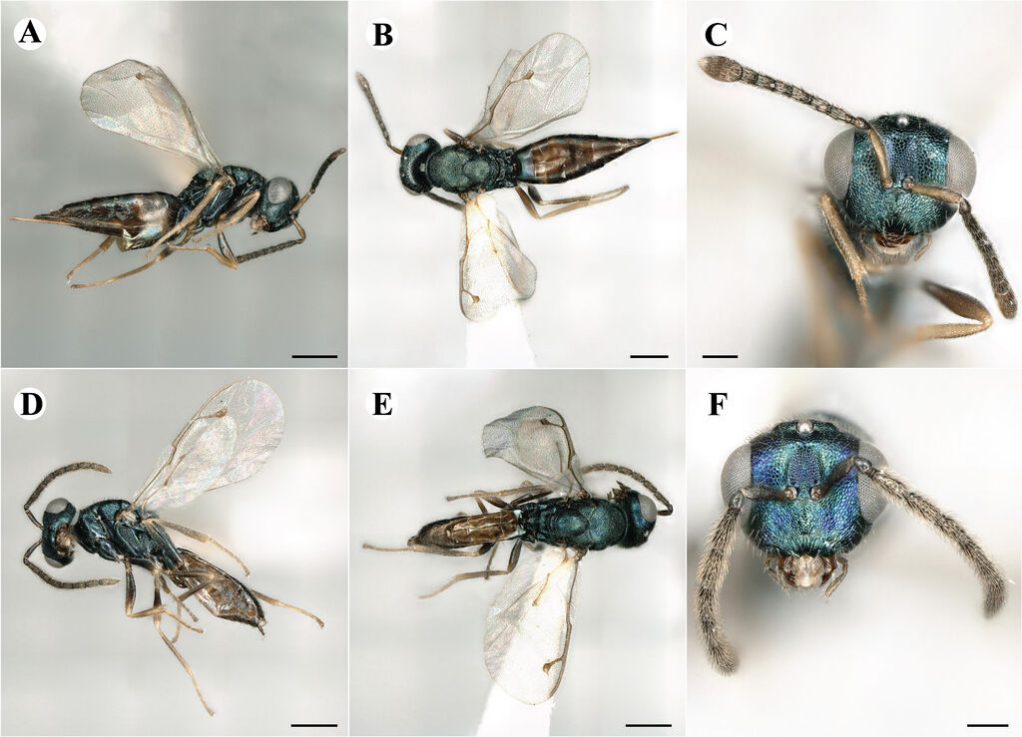
Digital microscopic images of *Apeliomapteromalinum* (Thomson, 1878) (♀, Pte_002853: **A - C**; ♂, Pte_002845: **D - F**). **A, D** Habitus lateral **B, E** Habitus dorsal **C, F** Head frontal. Scale bars: **A, B, D, E** 500 µm; **C, F** 200 µm.

Newly-recorded genus and species in Germany. The barcodes cluster in two subclusters, but no morphological distinction is possible. *Apeliomapteromalinum* (Thomson, 1878) might be cryptic. Images: Fig. 23.

#### Arthrolytus slovacus

Graham, 1969

ED492F78-BF10-58BA-A15E-5A900FBB202B

##### Materials

- **Type status:** Other material. **Occurrence:** catalogNumber: BOLD Sample ID: SMNS_37276; recordedBy: L. Krogmann, T. Kothe; sex: female; lifeStage: adult; preparations: dry mounted; associatedSequences: GenBank: OL538078; **Taxon:** scientificName: *Arthrolytusslovacus* Graham, 1969; **Location:** continent: Europe; country: Germany; countryCode: DE; stateProvince: Baden-Württemberg; locality: Lkr. Tübingen, Wurmlingen; verbatimElevation: 330 m; decimalLatitude: 48.5033; decimalLongitude: 8.9732; **Identification:** identifiedBy: H. Baur; dateIdentified: 2015; **Event:** samplingProtocol: Sweep net; eventDate: 5/9/2012; **Record Level:** datasetID: SMNS_Hym_Pte_000213; institutionCode: SMNS
- **Type status:** Other material. **Occurrence:** catalogNumber: BOLD Sample ID: SMNS_37278; recordedBy: L. Krogmann, T. Kothe; sex: male; lifeStage: adult; preparations: dry mounted; associatedSequences: GenBank: OL538110; **Taxon:** scientificName: *Arthrolytusslovacus* Graham, 1969; **Location:** continent: Europe; country: Germany; countryCode: DE; stateProvince: Baden-Württemberg; locality: Lkr. Tübingen, Wurmlingen; verbatimElevation: 330 m; decimalLatitude: 48.5033; decimalLongitude: 8.9732; **Identification:** identifiedBy: H. Baur; dateIdentified: 2015; **Event:** samplingProtocol: Sweep net; eventDate: 5/9/2012; **Record Level:** datasetID: SMNS_Hym_Pte_000215; institutionCode: SMNS

##### Ecological interactions

###### Parasite of

The host of the species is unknown, but members of the subgenus Arthrolytus are reported to be parasitoids of Cecidomyiidae, associated with Gramineae and members of the subgenus Anarthrolytus as being parasitoids of Hymenoptera (Cynipidae) or Coleoptera (Curculionidae), associated with Fagaceae (*Quercus* sp.).

##### Distribution

Eastern to northern Europe incl. The Netherlands, Germany: Baden-Württemberg

##### Notes

**Figure 24.**
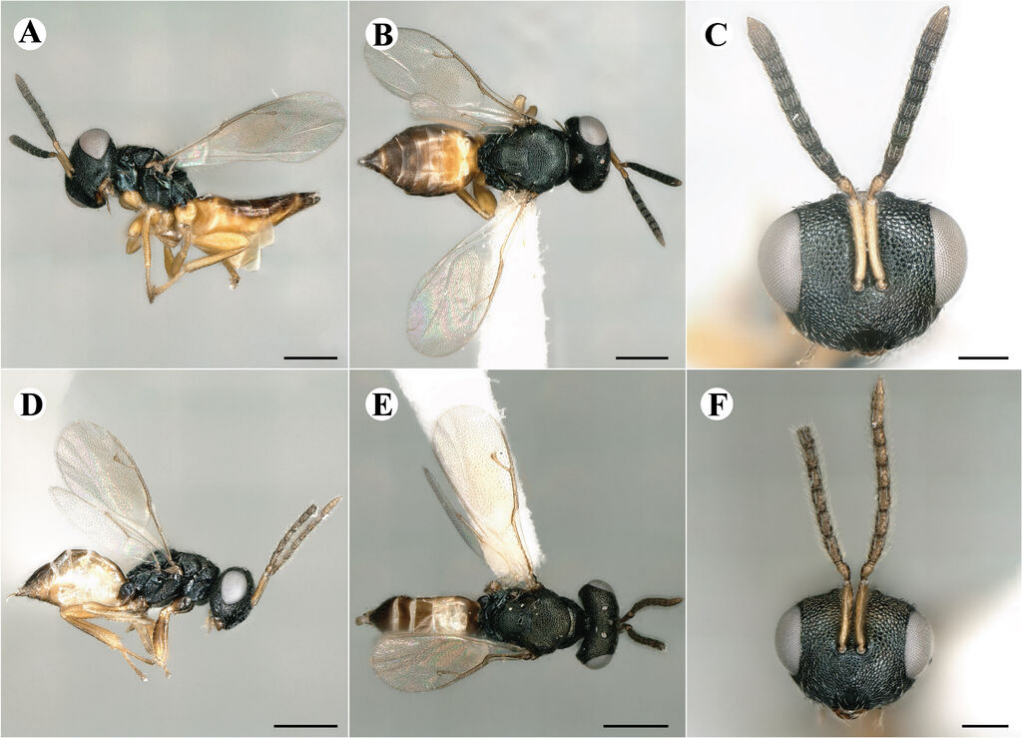
Digital microscopic images of *Arthrolytusslovacus* Graham, 1969 (♀, Pte_000213: **A - C**; ♂, Pte_000215: **D - F**). **A, D** Habitus lateral **B, E** Habitus dorsal **C, F** Head frontal. Scale bars: **A, B, D, E** 500 µm; **C, F** 200 µm.

Newly-recorded species in Germany. Images: Fig. 24.

#### Atrichomalus trianellatus

Graham, 1956

3CED814E-2CAB-57D7-AA4C-60C9E067CE6D

##### Materials

- **Type status:** Other material. **Occurrence:** catalogNumber: BOLD Sample ID: SMNS_38112; recordedBy: L. Krogmann; sex: female; lifeStage: adult; preparations: dry mounted; associatedSequences: GenBank: OL538102; **Taxon:** scientificName: *Atrichomalustrianellatus* Graham, 1956; **Location:** continent: Europe; country: Germany; countryCode: DE; stateProvince: Baden-Württemberg; locality: Stuttgart, Universität Hohenheim; verbatimElevation: 350 m; decimalLatitude: 48.7072; decimalLongitude: 9.2147; **Identification:** identifiedBy: H. Baur; dateIdentified: 2014; **Event:** samplingProtocol: Sweep net, Malaise trap, yellow pan trap; eventDate: 15/6/2013; **Record Level:** datasetID: SMNS_Hym_Pte_001049; institutionCode: SMNS

##### Ecological interactions

###### Parasite of

The host of the species is unknown.

##### Distribution

North, central and eastern Europe incl. United Kingdom; Germany: Baden-Württemberg

##### Notes

**Figure 25.**
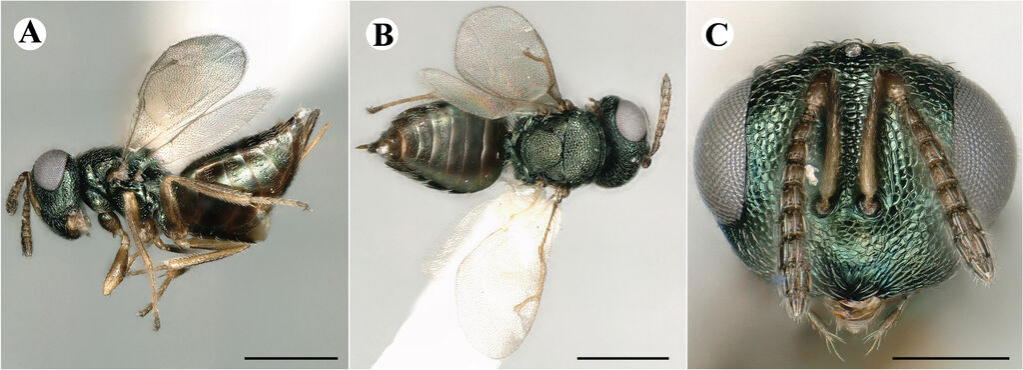
Digital microscopic images of *Atrichomalustrianellatus* Graham, 1956 (♀, Pte_001049). **A** Habitus lateral **B** Habitus dorsal **C** Head frontal. Scale bars: **A, B** 500 µm; **C** 200 µm.

Newly-recorded genus and species in Germany. Images: Fig. 25.

#### Coelopisthia pachycera

Masi, 1924

159A2AC0-861B-59F5-82FC-565789727567

##### Materials

- **Type status:** Other material. **Occurrence:** recordedBy: L. Krogmann, T. Kothe; sex: female; lifeStage: adult; preparations: dry mounted; **Taxon:** scientificName: *Coelopisthiapachycera* Masi, 1924; **Location:** continent: Europe; country: Germany; countryCode: DE; stateProvince: Baden-Württemberg; locality: Strohgäu, Markgröningen, EVS-Grundstück im Leudelsbachtal; verbatimElevation: 270 m; decimalLatitude: 48.9147; decimalLongitude: 9.0864; **Identification:** identifiedBy: H. Baur; dateIdentified: 2014; **Event:** samplingProtocol: Malaise trap; eventDate: 3/7 - 19/7/2011; **Record Level:** datasetID: SMNS_Hym_Pte_000136; institutionCode: SMNS

##### Ecological interactions

###### Parasite of

The species was reported as a parasitoid of Lepidoptera (Nymphalidae: *Maniolajurtina* (Linnaeus, 1758)) associated with Cyperaceae (*Carex* sp.), Resedaceae (*Resedalutea* L.), Saliaceae (*Salix* sp.).

##### Distribution

Europe and western Asia; Germany: Baden-Württemberg

##### Notes

**Figure 26.**
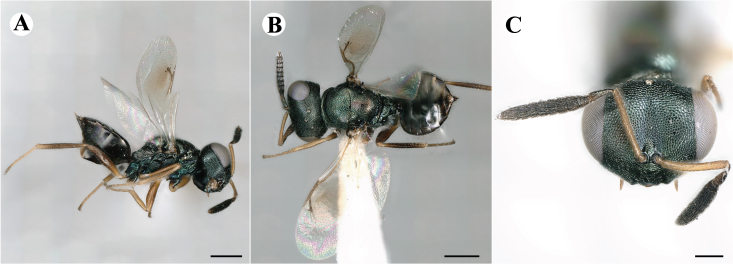
Digital microscopic images of *Coelopisthiapachycera* Masi, 1924 (♀, Pte_000136). **A** Habitus lateral **B** Habitus dorsal **C** Head frontal. Scale bars: **A, B** 500 µm; **C** 200 µm.

Newly-recorded species in Germany. No Barcode available. Images: Fig. 26.

#### Cryptoprymna paludicola

Askew, 1991

C926D916-2C11-5644-9F89-707ED3A87EC2

##### Materials

- **Type status:** Other material. **Occurrence:** catalogNumber: BOLD Sample ID: SMNS_39620; recordedBy: L. Krogmann, T. Kothe; sex: male; lifeStage: adult; preparations: dry mounted; associatedSequences: GenBank: OL538067; **Taxon:** scientificName: *Cryptoprymnapaludicola* Askew, 1991; **Location:** continent: Europe; country: Germany; countryCode: DE; stateProvince: Baden-Württemberg; locality: Lkr. Rems-Murr-Kreis, Wiesentäler bei der Menzlesmühle; verbatimElevation: 468 m; decimalLatitude: 48.9247; decimalLongitude: 9.6869; **Identification:** identifiedBy: M. Haas; dateIdentified: 2015; **Event:** samplingProtocol: Sweep net; eventDate: 10/7/2013; **Record Level:** datasetID: SMNS_Hym_Pte_002555; institutionCode: SMNS
- **Type status:** Other material. **Occurrence:** catalogNumber: BOLD Sample ID: SMNS_46615; recordedBy: F. Koch; sex: female; lifeStage: adult; preparations: dry mounted; associatedSequences: GenBank: OL538142; **Taxon:** scientificName: *Cryptoprymnapaludicola* Askew, 1991; **Location:** continent: Europe; country: Germany; countryCode: DE; stateProvince: Mecklenburg-Vorpommern; locality: Insel Rügen, Kniepow; verbatimElevation: 50 m; decimalLatitude: 54.3500; decimalLongitude: 13.3500; **Identification:** identifiedBy: M. Haas; dateIdentified: 2019; **Event:** samplingProtocol: Malaise trap; eventDate: 27/7 - 2/8/2014; **Record Level:** datasetID: SMNS_Hym_Pte_005385; institutionCode: SMNS

##### Ecological interactions

###### Parasite of

The host of the species is unknown, but members of the genus are reported to be parasitoids of Diptera (Agromyzidae) and Hemiptera (Aphididae), associated with Fabaceae.

##### Distribution

United Kingdom; Germany: Baden-Württemberg, Mecklenburg-Vorpommern

##### Notes

**Figure 27.**
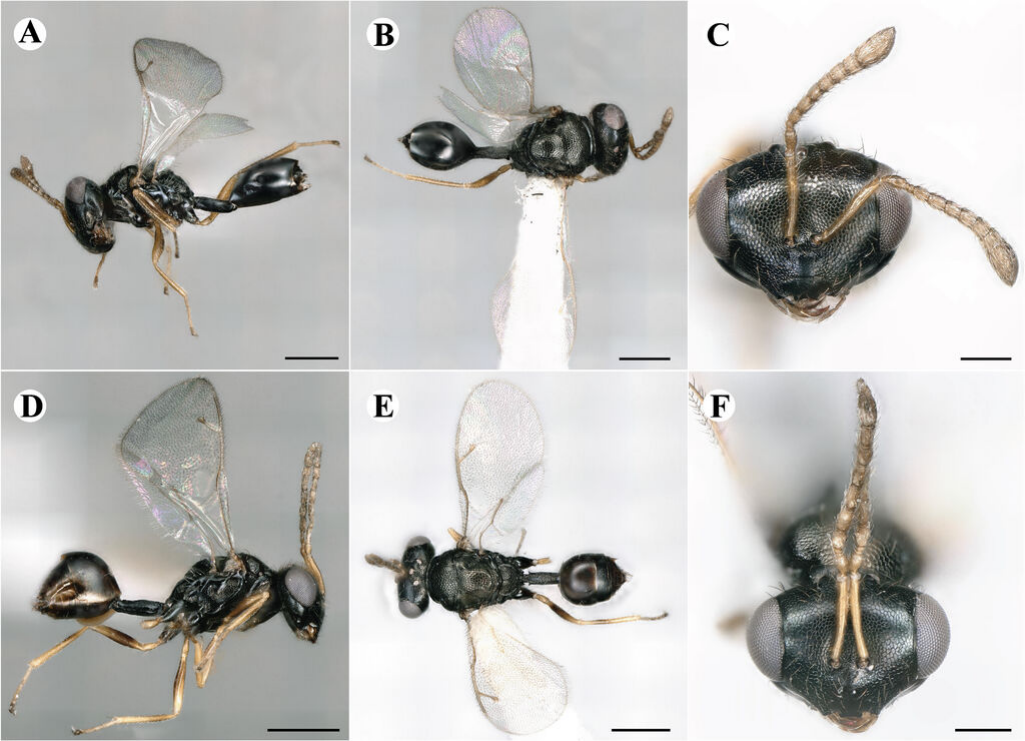
Digital microscopic images of *Cryptoprymnapaludicola* Askew, 1991 (♀, Pte_005385: **A - C**; ♂, Pte_002555: **D - F**). **A, D** Habitus lateral **B, E** Habitus dorsal **C, F** Head frontal. Scale bars: **A, B, D, E** 500 µm; **C, F** 200 µm.

Newly-recorded species in Germany. Images: Fig. 27.

#### Cyclogastrella clypealis

Bouček, 1965

0847143E-8C63-5CFE-A65A-8528A1A718DC

##### Materials

- **Type status:** Other material. **Occurrence:** catalogNumber: BOLD Sample ID: SMNS_38518; recordedBy: M. Thiv; sex: female; lifeStage: adult; preparations: dry mounted; associatedSequences: GenBank: OL538143; **Taxon:** scientificName: *Cyclogastrellaclypealis* Bouček, 1965; **Location:** continent: Europe; country: Germany; countryCode: DE; stateProvince: Baden-Württemberg; locality: Stuttgart, SMNS Zimmer Mike Thiv; **Identification:** identifiedBy: S. Bigalk; dateIdentified: 2016; **Event:** samplingProtocol: Manual collection; eventDate: 30/9/2013; **Record Level:** datasetID: SMNS_Hym_Pte_001455; institutionCode: SMNS
- **Type status:** Other material. **Occurrence:** catalogNumber: BOLD Sample ID: SMNS_39363; recordedBy: T. Kothe, C. König, J. Reibnitz; sex: female; lifeStage: adult; preparations: dry mounted; associatedSequences: GenBank: OL538144; **Taxon:** scientificName: *Cyclogastrellaclypealis* Bouček, 1965; **Location:** continent: Europe; country: Germany; countryCode: DE; stateProvince: Baden-Württemberg; locality: Lkr. Ostalbkreis, Goldberg; verbatimElevation: 480 m; decimalLatitude: 48.8608; decimalLongitude: 10.4232; **Identification:** identifiedBy: S. Bigalk; dateIdentified: 2016; **Event:** samplingProtocol: Sweep net; eventDate: 26/7/2013; **Record Level:** datasetID: SMNS_Hym_Pte_002300; institutionCode: SMNS
- **Type status:** Other material. **Occurrence:** catalogNumber: BOLD Sample ID: SMNS_39425; recordedBy: T. Kothe, C. König, J. Reibnitz; sex: female; lifeStage: adult; preparations: dry mounted; associatedSequences: GenBank: OL538106; **Taxon:** scientificName: *Cyclogastrellaclypealis* Bouček, 1965; **Location:** continent: Europe; country: Germany; countryCode: DE; stateProvince: Baden-Württemberg; locality: Lkr. Main-Tauber-Kreis, Truppenübungsplatz Tauberbischoffsheim; verbatimElevation: 339 m; decimalLatitude: 49.6246; decimalLongitude: 9.7079; **Identification:** identifiedBy: S. Bigalk; dateIdentified: 2016; **Event:** samplingProtocol: Sweep net; eventDate: 18/7/2013; **Record Level:** datasetID: SMNS_Hym_Pte_002362; institutionCode: SMNS
- **Type status:** Other material. **Occurrence:** catalogNumber: BOLD Sample ID: SMNS_39556; recordedBy: L. Krogmann; sex: male; lifeStage: adult; preparations: dry mounted; associatedSequences: GenBank: OL538060; **Taxon:** scientificName: *Cyclogastrellaclypealis* Bouček, 1965; **Location:** continent: Europe; country: Germany; countryCode: DE; stateProvince: Niedersachsen; locality: Lkr. Lüchow-Dannenberg, Nemitzer Heide; verbatimElevation: 24 m; decimalLatitude: 53.0007; decimalLongitude: 11.3462; **Identification:** identifiedBy: S. Bigalk; dateIdentified: 2016; **Event:** samplingProtocol: Sweep net; eventDate: 9/8/2013; **Record Level:** datasetID: SMNS_Hym_Pte_002491; institutionCode: SMNS
- **Type status:** Other material. **Occurrence:** catalogNumber: BOLD Sample ID: SMNS_39959; recordedBy: A. Nasahl; sex: female; lifeStage: adult; preparations: dry mounted; associatedSequences: GenBank: OL538100; **Taxon:** scientificName: *Cyclogastrellaclypealis* Bouček, 1965; **Location:** continent: Europe; country: Germany; countryCode: DE; stateProvince: Baden-Württemberg; locality: Stuttgart, Wilhelma, Hutewald, alt; verbatimElevation: 258 m; decimalLatitude: 48.8066; decimalLongitude: 9.1995; **Identification:** identifiedBy: S. Bigalk; dateIdentified: 2016; **Event:** samplingProtocol: Malaise trap; eventDate: 4/4 - 25/4/2014; **Record Level:** datasetID: SMNS_Hym_Pte_002894; institutionCode: SMNS
- **Type status:** Other material. **Occurrence:** catalogNumber: BOLD Sample ID: SMNS_40938; recordedBy: T. Kothe, M. Engelhardt, C. König; sex: female; lifeStage: adult; preparations: dry mounted; associatedSequences: GenBank: OL538069; **Taxon:** scientificName: *Cyclogastrellaclypealis* Bouček, 1965; **Location:** continent: Europe; country: Germany; countryCode: DE; stateProvince: Baden-Württemberg; locality: Tübingen, Steinenberg, Flurstücknummer 2970, MF 5; verbatimElevation: 483 m; decimalLatitude: 48.5355; decimalLongitude: 9.0332; **Identification:** identifiedBy: M. Haas; dateIdentified: 2021; **Event:** samplingProtocol: Malaise trap; eventDate: 14/8 - 29/8/2014; **Record Level:** datasetID: SMNS_Hym_Pte_003873; institutionCode: SMNS
- **Type status:** Other material. **Occurrence:** catalogNumber: BOLD Sample ID: SMNS_41027; recordedBy: T. Kothe, M. Engelhardt, C. König; sex: male; lifeStage: adult; preparations: dry mounted; associatedSequences: GenBank: OL538096; **Taxon:** scientificName: *Cyclogastrellaclypealis* Bouček, 1965; **Location:** continent: Europe; country: Germany; countryCode: DE; stateProvince: Baden-Württemberg; locality: Tübingen, Hirschau, Wiesenweingärten, Flurstücknummer 3923, MF 9; verbatimElevation: 382 m; decimalLatitude: 48.5043; decimalLongitude: 8.9956; **Identification:** identifiedBy: M. Haas; dateIdentified: 2021; **Event:** samplingProtocol: Malaise trap; eventDate: 31/7 - 12/9/2014; **Record Level:** datasetID: SMNS_Hym_Pte_003962; institutionCode: SMNS
- **Type status:** Other material. **Occurrence:** catalogNumber: BOLD Sample ID: SMNS_41043; recordedBy: T. Kothe, M. Engelhardt, C. König; sex: male; lifeStage: adult; preparations: dry mounted; associatedSequences: GenBank: OL538108; **Taxon:** scientificName: *Cyclogastrellaclypealis* Bouček, 1965; **Location:** continent: Europe; country: Germany; countryCode: DE; stateProvince: Baden-Württemberg; locality: Tübingen, Hirschau, Wiesenweingärten, Flurstücknummer 3923, MF 9; verbatimElevation: 382 m; decimalLatitude: 48.5043; decimalLongitude: 8.9956; **Identification:** identifiedBy: M. Haas; dateIdentified: 2021; **Event:** samplingProtocol: Malaise trap; eventDate: 31/7 - 12/9/2014; **Record Level:** datasetID: SMNS_Hym_Pte_003978; institutionCode: SMNS
- **Type status:** Other material. **Occurrence:** catalogNumber: BOLD Sample ID: SMNS_45995; recordedBy: T. Kothe, M. Engelhardt, C. König; sex: female; lifeStage: adult; preparations: dry mounted; associatedSequences: GenBank: OL538061; **Taxon:** scientificName: *Cyclogastrellaclypealis* Bouček, 1965; **Location:** continent: Europe; country: Germany; countryCode: DE; stateProvince: Baden-Württemberg; locality: Tübingen, Steinenberg, (Flurstücknummer 2910 Z. T., MF 3; verbatimElevation: 492 m; decimalLatitude: 48.5313; decimalLongitude: 9.0300; **Identification:** identifiedBy: M. Haas; dateIdentified: 2021; **Event:** samplingProtocol: Malaise trap; eventDate: 14/8 - 29/8/2014; **Record Level:** datasetID: SMNS_Hym_Pte_005282; institutionCode: SMNS
- **Type status:** Other material. **Occurrence:** catalogNumber: BOLD Sample ID: SMNS_49890; recordedBy: T. Kothe, M. Engelhardt, C. König; sex: female; lifeStage: adult; preparations: dry mounted; associatedSequences: GenBank: OL538072; **Taxon:** scientificName: *Cyclogastrellaclypealis* Bouček, 1965; **Location:** continent: Europe; country: Germany; countryCode: DE; stateProvince: Baden-Württemberg; locality: Lkr. Tübingen, Wurmlingen, Gengental, Flurstücknummer 3104, MF 7; verbatimElevation: 377 m; decimalLatitude: 48.5132; decimalLongitude: 8.9918; **Identification:** identifiedBy: M. Haas; dateIdentified: 2021; **Event:** samplingProtocol: Malaise trap; eventDate: 14/8 - 29/8/2014; **Record Level:** datasetID: SMNS_Hym_Pte_006341; institutionCode: SMNS
- **Type status:** Other material. **Occurrence:** catalogNumber: BOLD Sample ID: SMNS_49897; recordedBy: T. Kothe, M. Engelhardt, C. König; sex: female; lifeStage: adult; preparations: dry mounted; associatedSequences: GenBank: OL538066; **Taxon:** scientificName: *Cyclogastrellaclypealis* Bouček, 1965; **Location:** continent: Europe; country: Germany; countryCode: DE; stateProvince: Baden-Württemberg; locality: Lkr. Tübingen, Wurmlingen, Gengental, Flurstücknummer 3104, MF 7; verbatimElevation: 377 m; decimalLatitude: 48.5132; decimalLongitude: 8.9918; **Identification:** identifiedBy: M. Haas; dateIdentified: 2021; **Event:** samplingProtocol: Malaise trap; eventDate: 14/8 - 29/8/2014; **Record Level:** datasetID: SMNS_Hym_Pte_006348; institutionCode: SMNS
- **Type status:** Other material. **Occurrence:** catalogNumber: BOLD Sample ID: SMNS_49899; recordedBy: T. Kothe, M. Engelhardt, C. König; sex: female; lifeStage: adult; preparations: dry mounted; associatedSequences: GenBank: OL538115; **Taxon:** scientificName: *Cyclogastrellaclypealis* Bouček, 1965; **Location:** continent: Europe; country: Germany; countryCode: DE; stateProvince: Baden-Württemberg; locality: Lkr. Tübingen, Wurmlingen, Gengental, Flurstücknummer 3104, MF 7; verbatimElevation: 377 m; decimalLatitude: 48.5132; decimalLongitude: 8.9918; **Identification:** identifiedBy: M. Haas; dateIdentified: 2021; **Event:** samplingProtocol: Malaise trap; eventDate: 14/8 - 29/8/2014; **Record Level:** datasetID: SMNS_Hym_Pte_006350; institutionCode: SMNS
- **Type status:** Other material. **Occurrence:** catalogNumber: BOLD Sample ID: SMNS_49900; recordedBy: T. Kothe, M. Engelhardt, C. König; sex: female; lifeStage: adult; preparations: dry mounted; associatedSequences: GenBank: OL538083; **Taxon:** scientificName: *Cyclogastrellaclypealis* Bouček, 1965; **Location:** continent: Europe; country: Germany; countryCode: DE; stateProvince: Baden-Württemberg; locality: Lkr. Tübingen, Wurmlingen, Gengental, Flurstücknummer 3104, MF 7; verbatimElevation: 377 m; decimalLatitude: 48.5132; decimalLongitude: 8.9918; **Identification:** identifiedBy: M. Haas; dateIdentified: 2021; **Event:** samplingProtocol: Malaise trap; eventDate: 14/8 - 29/8/2014; **Record Level:** datasetID: SMNS_Hym_Pte_006351; institutionCode: SMNS
- **Type status:** Other material. **Occurrence:** catalogNumber: BOLD Sample ID: SMNS_50035; recordedBy: J. Aronov; sex: female; lifeStage: adult; preparations: dry mounted; associatedSequences: GenBank: OL538119; **Taxon:** scientificName: *Cyclogastrellaclypealis* Bouček, 1965; **Location:** continent: Europe; country: Germany; countryCode: DE; stateProvince: Baden-Württemberg; locality: Stuttgart, Wilhelma, Futtergarten; verbatimElevation: 238 m; decimalLatitude: 48.8040; decimalLongitude: 9.2052; **Identification:** identifiedBy: M. Haas; dateIdentified: 2021; **Event:** samplingProtocol: Malaise trap; eventDate: 1/7 - 31/7/2014; **Record Level:** datasetID: SMNS_Hym_Pte_006486; institutionCode: SMNS
- **Type status:** Other material. **Occurrence:** recordedBy: L. Krogmann, T. Krogmann; sex: female; lifeStage: adult; preparations: dry mounted; **Taxon:** scientificName: *Cyclogastrellaclypealis* Bouček, 1965; **Location:** continent: Europe; country: Germany; countryCode: DE; stateProvince: Baden-Württemberg; locality: Nordschwarzwald, Hundsbach, Kapellenstraße 9A; verbatimElevation: 688 m; decimalLatitude: 48.6170; decimalLongitude: 8.2756; **Identification:** identifiedBy: L. Krogmann; dateIdentified: 2018; **Event:** samplingProtocol: Manual collecting; eventDate: 28/10 - 3/11/2018; **Record Level:** datasetID: SMNS_Hym_Pte_008102; institutionCode: SMNS
- **Type status:** Other material. **Occurrence:** recordedBy: I. Wendt; sex: female; lifeStage: adult; preparations: dry mounted; **Taxon:** scientificName: *Cyclogastrellaclypealis* Bouček, 1965; **Location:** continent: Europe; country: Germany; countryCode: DE; stateProvince: Baden-Württemberg; locality: Lkr. ES, Esslingen; verbatimElevation: 335 m; decimalLatitude: 48.7473; decimalLongitude: 9.3326; **Identification:** identifiedBy: M. Haas; dateIdentified: 2021; **Event:** samplingProtocol: Totfund; eventDate: 9/5/2021; **Record Level:** datasetID: SMNS_Hym_Pte_011036; institutionCode: SMNS

##### Ecological interactions

###### Parasite of

The host of the species is unknown, but members of the genus are reported to be parasitoids of Lepidoptera pupae (Totricidae), associated with Taxodiaceae.

##### Distribution

Whole of Europe and northern Africa; Germany: Baden-Württemberg, Niedersachsen

##### Notes

**Figure 28.**
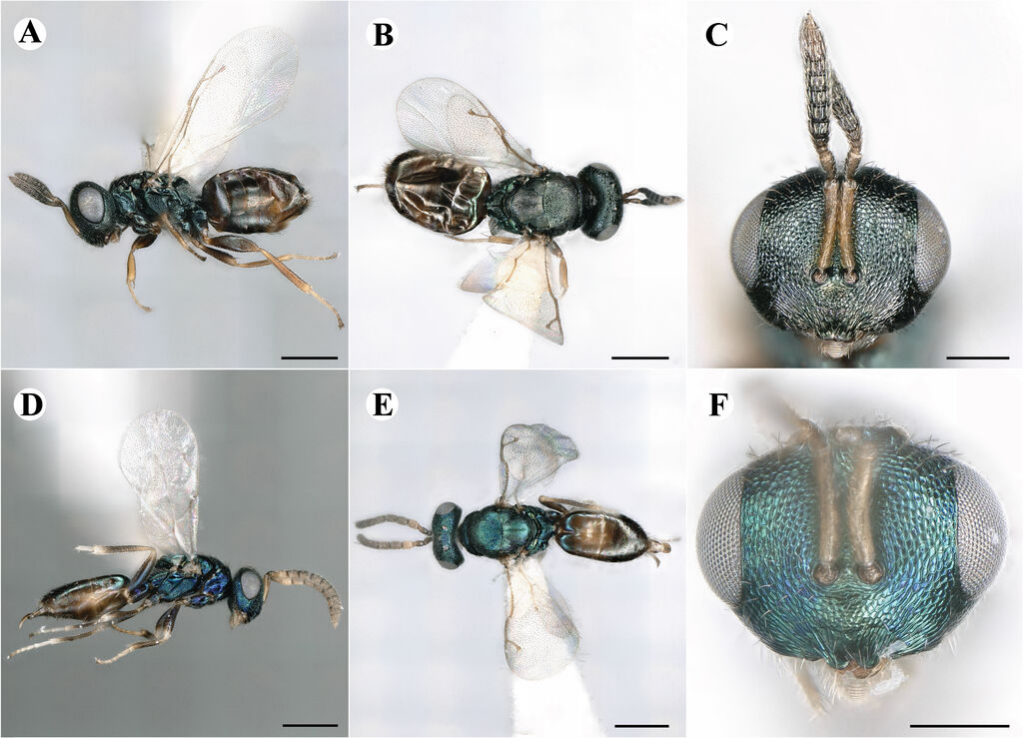
Digital microscopic images of *Cyclogastrellaclypealis* Bouček, 1965 (♀, Pte_002894: **A - C**; ♂, Pte_003978: **D - F**). **A, D** Habitus lateral **B, E** Habitus dorsal **C, F** Head frontal. Scale bars: **A, B, D, E** 500 µm; **C, F** 200 µm.

Newly-recorded species in Germany. Images: Fig. 28

#### Dibrachys hians

Bouček, 1965

49A9F90C-8BF6-5A5B-8DDD-E42034D0985E

##### Materials

- **Type status:** Other material. **Occurrence:** catalogNumber: BOLD Sample ID: SMNS_47347; recordedBy: T. Kothe, M. Engelhardt, C. König; sex: female; lifeStage: adult; preparations: dry mounted; associatedSequences: GenBank: OL538154; **Taxon:** scientificName: *Dibrachyshians* Bouček, 1965; **Location:** continent: Europe; country: Germany; countryCode: DE; stateProvince: Baden-Württemberg; locality: Lkr. Tübingen, Wurmlingen, Gengental, Flurstücknummer 3104, MF 7; verbatimElevation: 377 m; decimalLatitude: 48.5132; decimalLongitude: 8.9918; **Identification:** identifiedBy: M. Haas; dateIdentified: 2019; **Event:** samplingProtocol: Malaise trap; eventDate: 13/5 - 23/5/2014; **Record Level:** datasetID: SMNS_Hym_Pte_005520; institutionCode: SMNS

##### Ecological interactions

###### Parasite of

The host of the species is unknown, but members of the genus are reported to be parasitoids of Lepidoptera pupae (Pyralidae), associated with Taxodiaceae. Members of the genus tend to be hyperparasitoids.

##### Distribution

Whole of Europe; Germany: Baden-Württemberg

##### Notes

**Figure 29.**
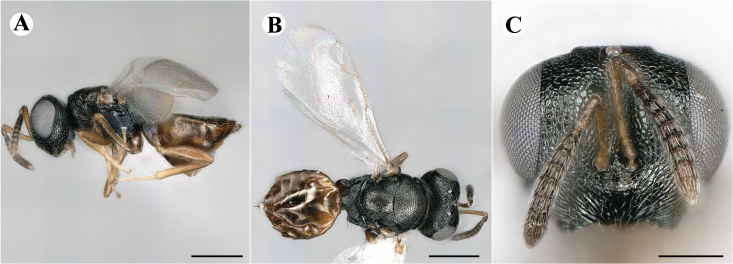
Digital microscopic images of *Dibrachyshians* Bouček, 1965 (♀, Pte_005520). **A** Habitus lateral **B** Habitus dorsal **C** Head frontal. Scale bars: **A, B** 500 µm; **C** 200 µm.

Newly-recorded species in Germany. Images: Fig. 29.

#### Dinotoides tenebricus

(Walker, 1834)

C05DFE4D-C22A-5AAC-A40E-E7678811106A

##### Materials

- **Type status:** Other material. **Occurrence:** catalogNumber: BOLD Sample ID: SMNS_37575; recordedBy: H.-J. Flügel; sex: female; lifeStage: adult; preparations: dry mounted; associatedSequences: GenBank: OL538057; **Taxon:** scientificName: *Dinotoidestenebricus* (Walker, 1834); **Location:** continent: Europe; country: Germany; countryCode: DE; stateProvince: Hessen; locality: Lkr. Schwalm-Eder-Kreis, Neumorschen, Halberg, 5202g; verbatimElevation: 196 m; decimalLatitude: 51.0628; decimalLongitude: 9.6022; **Identification:** identifiedBy: H. Baur; dateIdentified: 2014; **Event:** eventDate: 16/10/2012; **Record Level:** datasetID: SMNS_Hym_Pte_000512; institutionCode: SMNS
- **Type status:** Other material. **Occurrence:** recordedBy: H.-J. Flügel; sex: female; lifeStage: adult; preparations: dry mounted; **Taxon:** scientificName: *Dinotoidestenebricus* (Walker, 1834); **Location:** continent: Europe; country: Germany; countryCode: DE; stateProvince: Hessen; locality: Lkr. Schwalm-Eder-Kreis, Neumorschen, Halberg, 5202g; verbatimElevation: 196 m; decimalLatitude: 51.0628; decimalLongitude: 9.6022; **Identification:** identifiedBy: H. Baur; dateIdentified: 2014; **Event:** eventDate: 16/10/2012; **Record Level:** datasetID: SMNS_Hym_Pte_000532; institutionCode: SMNS
- **Type status:** Other material. **Occurrence:** catalogNumber: BOLD Sample ID: SMNS_39862; recordedBy: B. Rulik; sex: female; lifeStage: adult; preparations: dry mounted; associatedSequences: GenBank: OL538088; **Taxon:** scientificName: *Dinotoidestenebricus* (Walker, 1834); **Location:** continent: Europe; country: Germany; countryCode: DE; stateProvince: Rheinland-Pfalz; locality: Kreis Mayen-Koblenz, Winningen, Moseltal, Schieferhang, MF3, ZFMK-MAL-0001213; verbatimElevation: 230 m; decimalLatitude: 50.3224; decimalLongitude: 7.4939; **Identification:** identifiedBy: M. Haas; dateIdentified: 2021; **Event:** samplingProtocol: Malaise trap; eventDate: 2/5 - 16/5/2013; **Record Level:** datasetID: SMNS_Hym_Pte_002797; institutionCode: SMNS
- **Type status:** Other material. **Occurrence:** catalogNumber: BOLD Sample ID: SMNS_39864; recordedBy: B. Rulik; sex: female; lifeStage: adult; preparations: dry mounted; associatedSequences: GenBank: OL538126; **Taxon:** scientificName: *Dinotoidestenebricus* (Walker, 1834); **Location:** continent: Europe; country: Germany; countryCode: DE; stateProvince: Rheinland-Pfalz; locality: Kreis Mayen-Koblenz, Winningen, Moseltal, Schieferhang, MF3, ZFMK-MAL-0001197; verbatimElevation: 230 m; decimalLatitude: 50.3224; decimalLongitude: 7.4939; **Identification:** identifiedBy: M. Haas; dateIdentified: 2021; **Event:** samplingProtocol: Malaise trap; eventDate: 16/5 - 29/5/2013; **Record Level:** datasetID: SMNS_Hym_Pte_002799; institutionCode: SMNS
- **Type status:** Other material. **Occurrence:** catalogNumber: BOLD Sample ID: SMNS_39919; recordedBy: T. Kothe, G. Schweizer; sex: female; lifeStage: adult; preparations: dry mounted; associatedSequences: GenBank: OL538063; **Taxon:** scientificName: *Dinotoidestenebricus* (Walker, 1834); **Location:** continent: Europe; country: Germany; countryCode: DE; stateProvince: Baden-Württemberg; locality: Lkr. Esslingen, Oberboihingen; verbatimElevation: 307 m; decimalLatitude: 48.6506; decimalLongitude: 9.3689; **Identification:** identifiedBy: M. Haas; dateIdentified: 2021; **Event:** samplingProtocol: Malaise trap; eventDate: 4/4 - 4/5/2014; **Record Level:** datasetID: SMNS_Hym_Pte_002854; institutionCode: SMNS
- **Type status:** Other material. **Occurrence:** catalogNumber: BOLD Sample ID: SMNS_39932; recordedBy: T. Kothe, G. Schweizer; sex: female; lifeStage: adult; preparations: dry mounted; associatedSequences: GenBank: OL538059; **Taxon:** scientificName: *Dinotoidestenebricus* (Walker, 1834); **Location:** continent: Europe; country: Germany; countryCode: DE; stateProvince: Baden-Württemberg; locality: Lkr. Esslingen, Oberboihingen; verbatimElevation: 307 m; decimalLatitude: 48.6506; decimalLongitude: 9.3689; **Identification:** identifiedBy: M. Haas; dateIdentified: 2021; **Event:** samplingProtocol: Malaise trap; eventDate: 4/4 - 4/5/2014; **Record Level:** datasetID: SMNS_Hym_Pte_002867; institutionCode: SMNS
- **Type status:** Other material. **Occurrence:** catalogNumber: BOLD Sample ID: SMNS_39976; recordedBy: I. Wendt; sex: male; lifeStage: adult; preparations: dry mounted; associatedSequences: GenBank: OL538145; **Taxon:** scientificName: *Dinotoidestenebricus* (Walker, 1834); **Location:** continent: Europe; country: Germany; countryCode: DE; stateProvince: Baden-Württemberg; locality: Lkr. Esslingen, Thomashardt, Waldrand; verbatimElevation: 434 m; decimalLatitude: 48.7514; decimalLongitude: 9.4795; **Identification:** identifiedBy: M. Haas; dateIdentified: 2021; **Event:** samplingProtocol: Malaise trap; eventDate: 26/3 - 9/4/2014; **Record Level:** datasetID: SMNS_Hym_Pte_002911; institutionCode: SMNS
- **Type status:** Other material. **Occurrence:** catalogNumber: BOLD Sample ID: SMNS_39978; recordedBy: I. Wendt; sex: female; lifeStage: adult; preparations: dry mounted; associatedSequences: GenBank: OL538146; **Taxon:** scientificName: *Dinotoidestenebricus* (Walker, 1834); **Location:** continent: Europe; country: Germany; countryCode: DE; stateProvince: Baden-Württemberg; locality: Lkr. Esslingen, Thomashardt, Waldrand; verbatimElevation: 434 m; decimalLatitude: 48.7514; decimalLongitude: 9.4795; **Identification:** identifiedBy: M. Haas; dateIdentified: 2021; **Event:** samplingProtocol: Malaise trap; eventDate: 26/3 - 9/4/2014; **Record Level:** datasetID: SMNS_Hym_Pte_002913; institutionCode: SMNS
- **Type status:** Other material. **Occurrence:** catalogNumber: BOLD Sample ID: SMNS_40403; recordedBy: B. Rulik; sex: female; lifeStage: adult; preparations: dry mounted; associatedSequences: GenBank: OL538132; **Taxon:** scientificName: *Dinotoidestenebricus* (Walker, 1834); **Location:** continent: Europe; country: Germany; countryCode: DE; stateProvince: Rheinland-Pfalz; locality: Kreis Ahrweiler, Bausenberg, Niederzissen, Trockenrasen, MF5, ZFMK-MAL-0000215; verbatimElevation: 321 m; decimalLatitude: 50.4684; decimalLongitude: 7.2220; **Identification:** identifiedBy: M. Haas; dateIdentified: 2021; **Event:** samplingProtocol: Malaise trap; eventDate: 4/10 - 18/10/2012; **Record Level:** datasetID: SMNS_Hym_Pte_003338; institutionCode: SMNS
- **Type status:** Other material. **Occurrence:** catalogNumber: BOLD Sample ID: SMNS_41173; recordedBy: F. Koch; sex: male; lifeStage: adult; preparations: dry mounted; associatedSequences: GenBank: OL538147; **Taxon:** scientificName: *Dinotoidestenebricus* (Walker, 1834); **Location:** continent: Europe; country: Germany; countryCode: DE; stateProvince: Mecklenburg-Vorpommern; locality: Insel Rügen, Kniepow; verbatimElevation: 50 m; decimalLatitude: 54.3500; decimalLongitude: 13.3500; **Identification:** identifiedBy: M. Haas; dateIdentified: 2021; **Event:** samplingProtocol: Malaise trap; eventDate: 4/5 - 10/5/2014; **Record Level:** datasetID: SMNS_Hym_Pte_004108; institutionCode: SMNS

##### Ecological interactions

###### Parasite of

The species was reported as a parasitoid of wood-boring Coleoptera (Cerambycidae, Curculionidae and Scolytidae) in Fagaceae and Rosaceae.

##### Distribution

Northern, central, eastern and southern Europe incl. United Kingdom; Germany: Baden-Württemberg, Hessen, Mecklenburg-Vorpommern, Rheinland-Pfalz

##### Notes

**Figure 30.**
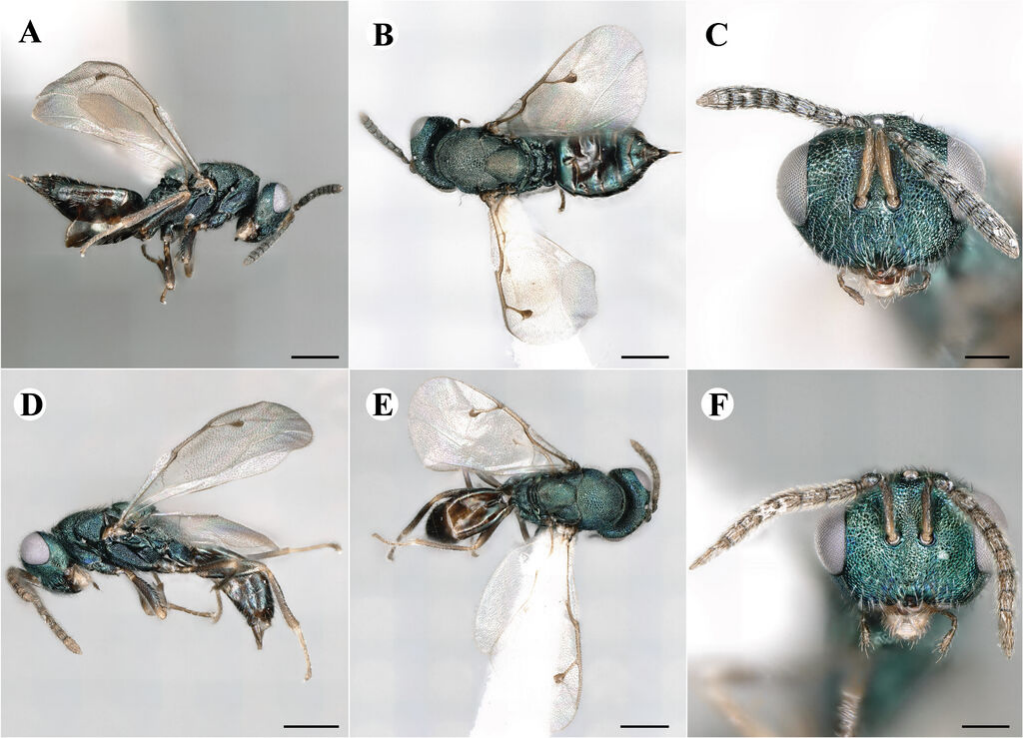
Digital microscopic images of *Dinotoidestenebricus* (Walker, 1834) (♀, Pte_002913: **A - C**; ♂, Pte_004108: **D - F**). **A, D** Habitus lateral **B, E** Habitus dorsal **C, F** Head frontal. Scale bars: **A, B, D, E** 500 µm; **C, F** 200 µm.

Newly-recorded genus and species in Germany. Images: Fig. 30

#### Erythromalus rufiventris

(Walker, 1835)

2CCA5A7D-0B5F-53AA-8548-36401261D991

##### Materials

- **Type status:** Other material. **Occurrence:** recordedBy: H.-J. Flügel; sex: female; lifeStage: adult; preparations: dry mounted; **Taxon:** scientificName: *Erythromalusrufiventris* (Walker, 1835); **Location:** continent: Europe; country: Germany; countryCode: DE; stateProvince: Hessen; locality: Lkr. Schwalm-Eder-Kreis, Neumorschen, Halberg, 5202g; verbatimElevation: 196 m; decimalLatitude: 51.0628; decimalLongitude: 9.6022; **Identification:** identifiedBy: H. Baur; dateIdentified: 2014; **Event:** eventDate: 16/10/2012; **Record Level:** datasetID: SMNS_Hym_Pte_000515; institutionCode: SMNS
- **Type status:** Other material. **Occurrence:** catalogNumber: BOLD Sample ID: SMNS_40078; recordedBy: B. Rulik; sex: female; lifeStage: adult; preparations: dry mounted; associatedSequences: GenBank: OL538109; **Taxon:** scientificName: *Erythromalusrufiventris* (Walker, 1835); **Location:** continent: Europe; country: Germany; countryCode: DE; stateProvince: Rheinland-Pfalz; locality: Kreis Ahrweiler, Niederzissen Bausenberg, Trockenrasen, MF5, ZFMK-MAL-0000719; verbatimElevation: 321 m; decimalLatitude: 50.4684; decimalLongitude: 7.2220; **Identification:** identifiedBy: H. Baur; dateIdentified: 2016; **Event:** samplingProtocol: Malaise trap; eventDate: 23/8 - 6/9/2012; **Record Level:** datasetID: SMNS_Hym_Pte_003013; institutionCode: SMNS
- **Type status:** Other material. **Occurrence:** catalogNumber: BOLD Sample ID: SMNS_47341; recordedBy: T. Kothe, M. Engelhardt, C. König; sex: male; lifeStage: adult; preparations: dry mounted; associatedSequences: GenBank: OL538111; **Taxon:** scientificName: *Erythromalusrufiventris* (Walker, 1835); **Location:** continent: Europe; country: Germany; countryCode: DE; stateProvince: Baden-Württemberg; locality: Lkr. Tübingen, Wurmlingen, Gengental, Flurstücknummer 3104, MF 7; verbatimElevation: 377 m; decimalLatitude: 48.5132; decimalLongitude: 8.9918; **Identification:** identifiedBy: M. Haas; dateIdentified: 2021; **Event:** samplingProtocol: Malaise trap; eventDate: 13/5 - 23/5/2014; **Record Level:** datasetID: SMNS_Hym_Pte_005514; institutionCode: SMNS

##### Ecological interactions

###### Parasite of

The host of the species is unknown, but members of the genus are reported to be associated with grasses in woods.

##### Distribution

Northern and eastern Europe incl. United Kingdom, northern Asia; Germany: Baden-Württemberg, Hessen, Rheinland-Pfalz

##### Notes

**Figure 31.**
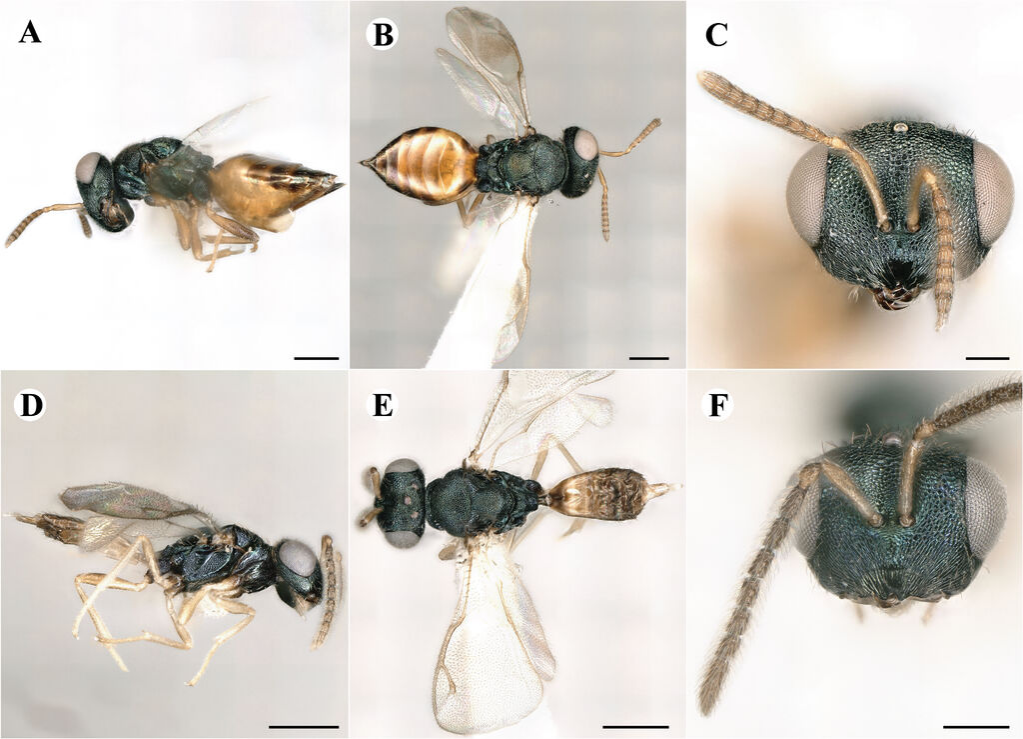
Digital microscopic images of *Erythromalusrufiventris* (Walker, 1835) (♀, Pte_000515: **A - C**; ♂, Pte 005514: **D - F**). **A, D** Habitus lateral **B, E** Habitus dorsal **C, F** Head frontal. Scale bars: **A, B, D, E** 500 µm; **C, F** 200 µm.

Newly-recorded genus and species in Germany. Images: Fig. 31.

#### Gbelcia crassiceps

Bouček, 1961

5C87F580-1A38-5484-901A-A2CEF9EED610

##### Materials

- **Type status:** Other material. **Occurrence:** catalogNumber: BOLD Sample ID: SMNS_38899; recordedBy: L. Krogmann, T. Kothe; sex: female; lifeStage: adult; preparations: dry mounted; associatedSequences: GenBank: OL538103; **Taxon:** scientificName: *Gbelciacrassiceps* Bouček, 1961; **Location:** continent: Europe; country: Germany; countryCode: DE; stateProvince: Sachsen; locality: Lkr. Bautzen, Guttau, 5); verbatimElevation: 143 m; decimalLatitude: 51.2556; decimalLongitude: 14.5844; **Identification:** identifiedBy: M. Haas; dateIdentified: 2021; **Event:** samplingProtocol: Sweep net; eventDate: 31/7/2013; **Record Level:** datasetID: SMNS_Hym_Pte_001836; institutionCode: SMNS

##### Ecological interactions

###### Parasite of

The host of the species is unknown, but it is probably associated with Poaceae (*Phragmites* sp.).

##### Distribution

Northern and eastern Europe incl. United Kingdom, central Asia; Germany: Sachsen

##### Notes

**Figure 32.**
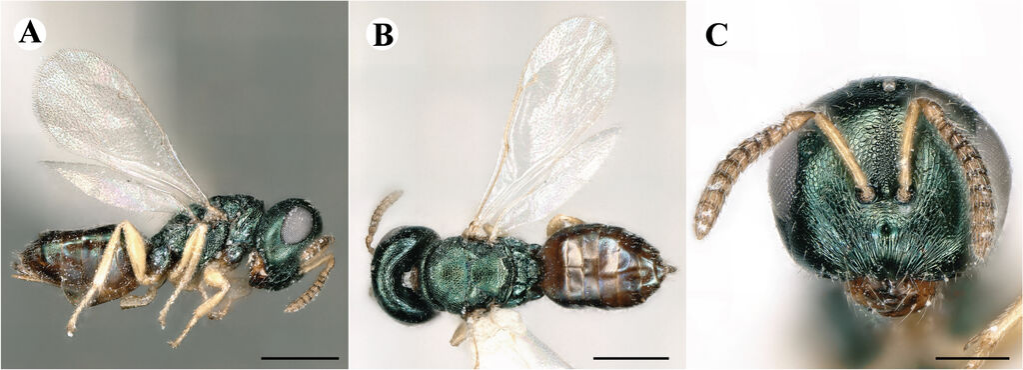
Digital microscopic images of *Gbelciacrassiceps* Bouček, 1961 (♀, Pte_001836). **A** Habitus lateral **B** Habitus dorsal **C** Head frontal. Scale bars: **A, B** 500 µm; **C** 200 µm.

Newly-recorded genus and species in Germany. Images: Fig. 32.

#### Heteroprymna longicornis

(Walker, 1835)

5B7BE8BF-CFF6-5BBF-812E-6318F50CD745

##### Materials

- **Type status:** Other material. **Occurrence:** catalogNumber: BOLD Sample ID: SMNS_37800; recordedBy: L. Krogmann, T. Kothe; sex: female; lifeStage: adult; preparations: dry mounted; associatedSequences: GenBank: OL538156; **Taxon:** scientificName: *Heteroprymnalongicornis* (Walker, 1835); **Location:** continent: Europe; country: Germany; countryCode: DE; stateProvince: Baden-Württemberg; locality: Strohgäu, Markgröningen, EVS-Grundstück im Leudelsbachtal; verbatimElevation: 270 m; decimalLatitude: 48.9147; decimalLongitude: 9.0864; **Identification:** identifiedBy: H. Baur; dateIdentified: 2014; **Event:** samplingProtocol: Malaise trap; eventDate: 1/8 - 15/8/2011; **Record Level:** datasetID: SMNS_Hym_Pte_000737; institutionCode: SMNS
- **Type status:** Other material. **Occurrence:** catalogNumber: BOLD Sample ID: SMNS_38246; recordedBy: D. Doczkal; sex: female; lifeStage: adult; preparations: dry mounted; associatedSequences: GenBank: OL538157; **Taxon:** scientificName: *Heteroprymnalongicornis* (Walker, 1835); **Location:** continent: Europe; country: Germany; countryCode: DE; stateProvince: Bayern; locality: Fussbergmoos 1, Fbm.1-10,; **Identification:** identifiedBy: H. Baur; dateIdentified: 2014; **Event:** samplingProtocol: Malaise trap; eventDate: 12/9/2011; **Record Level:** datasetID: SMNS_Hym_Pte_001183; institutionCode: SMNS

##### Ecological interactions

###### Parasite of

The host of the species is unknown, but it is probably associated with Malvaceae (*Tilia* sp.).

##### Distribution

Northern and eastern Europe incl. United Kingdom; Germany: Baden-Württemberg, Bayern

##### Notes

**Figure 33.**
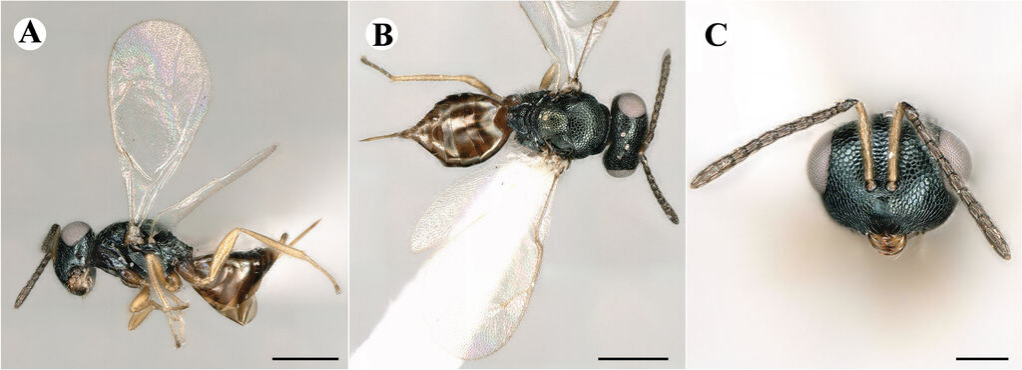
Digital microscopic images of *Heteroprymnalongicornis* (Walker, 1835) (♀, Pte_001183). **A** Habitus lateral **B** Habitus dorsal **C** Head frontal. Scale bars: **A, B** 500 µm; **C** 200 µm.

Newly-recorded genus and species in Germany. Images: Fig. 33

#### Kaleva corynocera

Graham, 1957

B32E73A9-F025-57D7-9EE8-804A4286DAD2

##### Materials

- **Type status:** Other material. **Occurrence:** catalogNumber: BOLD Sample ID: SMNSP027-19; recordedBy: L. Krogmann, T. Kothe; sex: female; lifeStage: adult; preparations: dry mounted; associatedSequences: GenBank: OL538138; **Taxon:** scientificName: *Kalevacorynocera* Graham, 1957; **Location:** continent: Europe; country: Germany; countryCode: DE; stateProvince: Baden-Württemberg; locality: Strohgäu, Markgröningen, EVS-Grundstück im Leudelsbachtal; verbatimElevation: 270 m; decimalLatitude: 48.9147; decimalLongitude: 9.0864; **Identification:** identifiedBy: H. Baur; dateIdentified: 2014; **Event:** samplingProtocol: Malaise trap; eventDate: 5/6 - 19/6/2011; **Record Level:** datasetID: SMNS_Hym_Pte_000149; institutionCode: SMNS
- **Type status:** Other material. **Occurrence:** catalogNumber: BOLD Sample ID: SMNS_37896; recordedBy: T. Kothe, G. Schweizer; sex: female; lifeStage: adult; preparations: dry mounted; associatedSequences: GenBank: OL538124; **Taxon:** scientificName: *Kalevacorynocera* Graham, 1957; **Location:** continent: Europe; country: Germany; countryCode: DE; stateProvince: Baden-Württemberg; locality: Lkr. Esslingen, Oberboihingen; verbatimElevation: 307 m; decimalLatitude: 48.6506; decimalLongitude: 9.3689; **Identification:** identifiedBy: H. Baur; dateIdentified: 2014; **Event:** samplingProtocol: Malaise trap; eventDate: 10/6 - 23/6/2012; **Record Level:** datasetID: SMNS_Hym_Pte_000833; institutionCode: SMNS
- **Type status:** Other material. **Occurrence:** catalogNumber: BOLD Sample ID: SMNS_37915; recordedBy: T. Kothe, G. Schweizer; sex: female; lifeStage: adult; preparations: dry mounted; associatedSequences: GenBank: OL538079; **Taxon:** scientificName: *Kalevacorynocera* Graham, 1957; **Location:** continent: Europe; country: Germany; countryCode: DE; stateProvince: Baden-Württemberg; locality: Lkr. Esslingen, Oberboihingen; verbatimElevation: 307 m; decimalLatitude: 48.6506; decimalLongitude: 9.3689; **Identification:** identifiedBy: L. Krogmann; **Event:** samplingProtocol: Malaise trap; eventDate: 10/6 - 23/6/2012; **Record Level:** datasetID: SMNS_Hym_Pte_000852; institutionCode: SMNS
- **Type status:** Other material. **Occurrence:** catalogNumber: BOLD Sample ID: SMNS_38626; recordedBy: T. Kothe, G. Schweizer; sex: male; lifeStage: adult; preparations: dry mounted; associatedSequences: GenBank: OL538058; **Taxon:** scientificName: *Kalevacorynocera* Graham, 1957; **Location:** continent: Europe; country: Germany; countryCode: DE; stateProvince: Baden-Württemberg; locality: Lkr. Esslingen, Oberboihingen; verbatimElevation: 307 m; decimalLatitude: 48.6506; decimalLongitude: 9.3689; **Identification:** identifiedBy: S. Bigalk; dateIdentified: 2016; **Event:** samplingProtocol: Malaise trap; eventDate: 16/7 - 12/8/2012; **Record Level:** datasetID: SMNS_Hym_Pte_001563; institutionCode: SMNS
- **Type status:** Other material. **Occurrence:** catalogNumber: BOLD Sample ID: SMNS_49887; recordedBy: T. Kothe, M. Engelhardt, C. König; sex: female; lifeStage: adult; preparations: dry mounted; associatedSequences: GenBank: OL538085; **Taxon:** scientificName: *Kalevacorynocera* Graham, 1957; **Location:** continent: Europe; country: Germany; countryCode: DE; stateProvince: Baden-Württemberg; locality: Lkr. Tübingen, Wurmlingen, Gengental, Flurstücknummer 3104, MF 7; verbatimElevation: 377 m; decimalLatitude: 48.5132; decimalLongitude: 8.9918; **Identification:** identifiedBy: M. Haas; dateIdentified: 2021; **Event:** samplingProtocol: Malaise trap; eventDate: 14/8 - 29/8/2014; **Record Level:** datasetID: SMNS_Hym_Pte_006338; institutionCode: SMNS

##### Ecological interactions

###### Parasite of

The species was reported as a parasitoid of Hymenoptera (Sphecidae: *Spilomena* sp.) in dead wood of Fagaceae (*Quercusrobur* L.).

##### Distribution

Europe and eastern Asia, incl. United Kingdom; Germany: Baden-Württemberg

##### Notes

**Figure 34.**
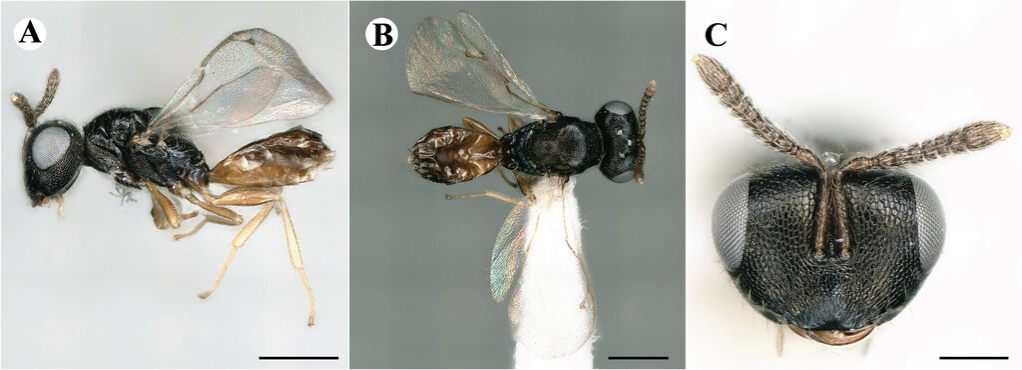
Digital microscopic images of *Kalevacorynocera* Graham, 1957 (♀, Pte_000852). **A** Habitus lateral **B** Habitus dorsal **C** Head frontal. Scale bars: **A, B** 500 µm; **C** 200 µm.

Newly-recorded genus and species in Germany. The barcodes cluster in subclusters, but no morphological distinction is possible. *Kalevacorynocera* Graham, 1957 might be cryptic. Images: Fig. 34.

#### Platygerrhus unicolor

Graham, 1969

D4D86D1B-6394-5FF6-9DCE-AA6FB7518252

##### Materials

- **Type status:** Other material. **Occurrence:** catalogNumber: BOLD Sample ID: SMNS_40115; recordedBy: B. Rulik; sex: female; lifeStage: adult; preparations: dry mounted; associatedSequences: GenBank: OL538104; **Taxon:** scientificName: *Platygerrhusunicolor* Graham, 1969; **Location:** continent: Europe; country: Germany; countryCode: DE; stateProvince: Rheinland-Pfalz; locality: Kreis Ahrweiler, Bausenberg, Eichen-Buchen-Mischwald, MF6, Niederzissen, ZFMK-MAL-0000091; verbatimElevation: 343 m; decimalLatitude: 50.4708; decimalLongitude: 7.2234; **Identification:** identifiedBy: H. Baur; dateIdentified: 2021; **Event:** samplingProtocol: Malaise trap; eventDate: 23/8 - 6/9/2012; **Record Level:** datasetID: SMNS_Hym_Pte_003050; institutionCode: SMNS
- **Type status:** Other material. **Occurrence:** catalogNumber: BOLD Sample ID: SMNS_50114; recordedBy: L. Krogmann, J. Holstein, T. Kothe; sex: female; lifeStage: adult; preparations: dry mounted; associatedSequences: GenBank: OL538127; **Taxon:** scientificName: *Platygerrhusunicolor* Graham, 1969; **Location:** continent: Europe; country: Germany; countryCode: DE; stateProvince: Baden-Württemberg; locality: Lkr. Rems-Murr-Kreis, Aspach bei Backnang; verbatimElevation: 368.5 m; decimalLatitude: 48.9990; decimalLongitude: 9.4197; **Identification:** identifiedBy: H. Baur; dateIdentified: 2021; **Event:** samplingProtocol: Malaise trap; eventDate: 17/6 - 15/7/2014; **Record Level:** datasetID: SMNS_Hym_Pte_006565; institutionCode: SMNS

##### Ecological interactions

###### Parasite of

The host of the species is unknown, but is associated with Betulaceae (*Alnusglutinosa* L.). Other members of the genus are reported to be parasitoids of Coleoptera (Anobiidae, Cucujidae and Scolytidae) in dead wood.

##### Distribution

Northern and eastern Europe incl. United Kingdom; Germany: Baden-Württemberg, Rheinland-Pfalz

##### Notes

**Figure 35.**
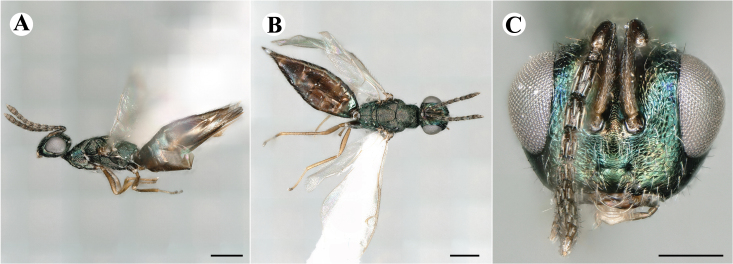
Digital microscopic images of *Platygerrhusunicolor* Graham, 1969 (♀, Pte_006565). **A** Habitus lateral **B** Habitus dorsal **C** Head frontal. Scale bars: **A, B** 500 µm; **C** 200 µm.

Newly-recorded species in Germany. Images: Fig. 35.

#### Psilocera confusa

Graham, 1992

468E6B5C-5EF8-5A1B-BC2F-6B0EB0FBA5FE

##### Materials

- **Type status:** Other material. **Occurrence:** catalogNumber: BOLD Sample ID: SMNS_39830; recordedBy: L. Krogmann; sex: female; lifeStage: adult; preparations: dry mounted; associatedSequences: GenBank: OL538117; **Taxon:** scientificName: *Psiloceraconfusa* Graham, 1992; **Location:** continent: Europe; country: Germany; countryCode: DE; stateProvince: Niedersachsen; locality: Lkr. Lüchow-Dannenberg, Höhbeck, Trockenrasen, 15); verbatimElevation: 64 m; decimalLatitude: 53.0694; decimalLongitude: 11.4350; **Identification:** identifiedBy: M. Haas; dateIdentified: 2021; **Event:** samplingProtocol: Yellow pan trap; eventDate: 10/8/2013; **Record Level:** datasetID: SMNS_Hym_Pte_002765; institutionCode: SMNS

##### Ecological interactions

###### Parasite of

The host of the species is unknown, but is associated with Ericaceae (*Erica scoparia* L.). Other members of the genus are reported from various hosts in Coleoptera and Lepidoptera.

##### Distribution

Western, central and eastern Europe incl. United Kingdom and western Asia; Germany: Hessen, Niedersachsen, Rheinland-Pfalz

##### Notes

**Figure 36.**
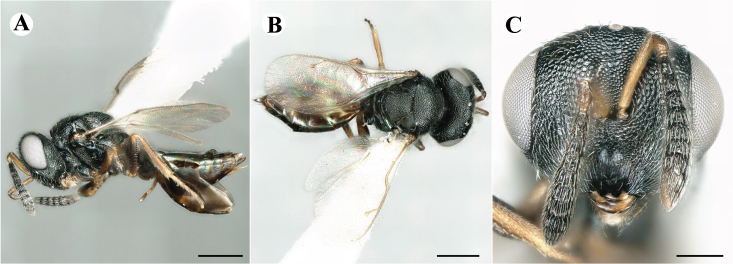
Digital microscopic images of *Psiloceraconfusa* Graham, 1992 (♀, Pte_002765). **A** Habitus lateral **B** Habitus dorsal **C** Head frontal. Scale bars: **A, B** 500 µm; **C** 200 µm.

Newly-recorded species in Germany. Images: Fig. 36.

#### Psilocera crassispina

(Thomson, 1878)

282D30D4-0E92-5F0B-9B13-479A46CE3E1F

##### Materials

- **Type status:** Other material. **Occurrence:** catalogNumber: BOLD Sample ID: SMNS_38015; recordedBy: T. Kothe, I. Wendt; sex: male; lifeStage: adult; preparations: dry mounted; associatedSequences: GenBank: OL538155; **Taxon:** scientificName: *Psiloceracrassispina* (Thomson, 1878); **Location:** continent: Europe; country: Germany; countryCode: DE; stateProvince: Baden-Württemberg; locality: Lkr. Ostalbkreis, Weiler i. d. B., Hornberg, Wacholderheide; verbatimElevation: 647 m; decimalLatitude: 48.7478; decimalLongitude: 9.8683; **Identification:** identifiedBy: M. Haas; dateIdentified: 2020; **Event:** samplingProtocol: Sweep net; eventDate: 15/5/2013; **Record Level:** datasetID: SMNS_Hym_Pte_000952; institutionCode: SMNS
- **Type status:** Other material. **Occurrence:** catalogNumber: BOLD Sample ID: SMNS_38415; recordedBy: H.-J. Flügel; sex: female; lifeStage: adult; preparations: dry mounted; associatedSequences: GenBank: OL538158; **Taxon:** scientificName: *Psiloceracrassispina* (Thomson, 1878); **Location:** continent: Europe; country: Germany; countryCode: DE; stateProvince: Hessen; locality: Lkr. Schwalm-Eder-Kreis, Neumorschen, Halberg, 5149a; verbatimElevation: 196 m; decimalLatitude: 51.0628; decimalLongitude: 9.6022; **Identification:** identifiedBy: M. Haas; dateIdentified: 2020; **Event:** samplingProtocol: Malaise trap; eventDate: 10/7 - 22/7/2012; **Record Level:** datasetID: SMNS_Hym_Pte_001352; institutionCode: SMNS
- **Type status:** Other material. **Occurrence:** catalogNumber: BOLD Sample ID: SMNS_38430; recordedBy: H.-J. Flügel; sex: male; lifeStage: adult; preparations: dry mounted; associatedSequences: GenBank: OL538070; **Taxon:** scientificName: *Psiloceracrassispina* (Thomson, 1878); **Location:** continent: Europe; country: Germany; countryCode: DE; stateProvince: Hessen; locality: Lkr. Schwalm-Eder-Kreis, Neumorschen, Halberg, 5149a; verbatimElevation: 196 m; decimalLatitude: 51.0628; decimalLongitude: 9.6022; **Identification:** identifiedBy: M. Haas; dateIdentified: 2020; **Event:** samplingProtocol: Malaise trap; eventDate: 10/7 - 22/7/2012; **Record Level:** datasetID: SMNS_Hym_Pte_001367; institutionCode: SMNS
- **Type status:** Other material. **Occurrence:** catalogNumber: BOLD Sample ID: SMNS_38775; recordedBy: L. Krogmann, T. Kothe; sex: female; lifeStage: adult; preparations: dry mounted; associatedSequences: GenBank: OL538114; **Taxon:** scientificName: *Psiloceracrassispina* (Thomson, 1878); **Location:** continent: Europe; country: Germany; countryCode: DE; stateProvince: Baden-Württemberg; locality: Lkr. Breisgau-Hochschwarzwald, NSG Badberg; verbatimElevation: 426 m; decimalLatitude: 48.0964; decimalLongitude: 7.6808; **Identification:** identifiedBy: H. Baur; dateIdentified: 2016; **Event:** samplingProtocol: Sweep net; eventDate: 18/6/2013; **Record Level:** datasetID: SMNS_Hym_Pte_001712; institutionCode: SMNS
- **Type status:** Other material. **Occurrence:** catalogNumber: BOLD Sample ID: SMNS_39201; recordedBy: H.-J. Flügel; sex: male; lifeStage: adult; preparations: dry mounted; associatedSequences: GenBank: OL538089; **Taxon:** scientificName: *Psiloceracrassispina* (Thomson, 1878); **Location:** continent: Europe; country: Germany; countryCode: DE; stateProvince: Hessen; locality: Lkr. Schwalm-Eder-Kreis, Neumorschen, Halberg, 5267g; verbatimElevation: 196 m; decimalLatitude: 51.0628; decimalLongitude: 9.6022; **Identification:** identifiedBy: M. Haas; dateIdentified: 2020; **Event:** samplingProtocol: Malaise trap; eventDate: 10/6 - 1/7/2013; **Record Level:** datasetID: SMNS_Hym_Pte_002138; institutionCode: SMNS
- **Type status:** Other material. **Occurrence:** catalogNumber: BOLD Sample ID: SMNS_39294; recordedBy: L. Krogmann, T. Kothe; sex: male; lifeStage: adult; preparations: dry mounted; associatedSequences: GenBank: OL538137; **Taxon:** scientificName: *Psiloceracrassispina* (Thomson, 1878); **Location:** continent: Europe; country: Germany; countryCode: DE; stateProvince: Baden-Württemberg; locality: Lkr. Main-Tauber-Kreis, Tauberbischofsheim, ex Truppenübungsplatz; verbatimElevation: 331 m; decimalLatitude: 49.6245; decimalLongitude: 9.7081; **Identification:** identifiedBy: H. Baur; dateIdentified: 2016; **Event:** samplingProtocol: Sweep net; eventDate: 12/6/2013; **Record Level:** datasetID: SMNS_Hym_Pte_002231; institutionCode: SMNS
- **Type status:** Other material. **Occurrence:** catalogNumber: BOLD Sample ID: SMNS_39401; recordedBy: T. Kothe, C. König, J. Reibnitz; sex: male; lifeStage: adult; preparations: dry mounted; associatedSequences: GenBank: OL538113; **Taxon:** scientificName: *Psiloceracrassispina* (Thomson, 1878); **Location:** continent: Europe; country: Germany; countryCode: DE; stateProvince: Baden-Württemberg; locality: Lkr. Ostalbkreis, Goldberg; verbatimElevation: 480 m; decimalLatitude: 48.8608; decimalLongitude: 10.4232; **Identification:** identifiedBy: M. Haas; dateIdentified: 2020; **Event:** samplingProtocol: Sweep net; eventDate: 26/7/2013; **Record Level:** datasetID: SMNS_Hym_Pte_002338; institutionCode: SMNS
- **Type status:** Other material. **Occurrence:** catalogNumber: BOLD Sample ID: SMNS_39552; recordedBy: L. Krogmann, T. Kothe, J. Reibnitz; sex: male; lifeStage: adult; preparations: dry mounted; associatedSequences: GenBank: OL538139; **Taxon:** scientificName: *Psiloceracrassispina* (Thomson, 1878); **Location:** continent: Europe; country: Germany; countryCode: DE; stateProvince: Baden-Württemberg; locality: Alb-Donau-Kreis, NSG Kleines Lautertal; verbatimElevation: 551 m; decimalLatitude: 48.4440; decimalLongitude: 9.8692; **Identification:** identifiedBy: M. Haas; dateIdentified: 2020; **Event:** samplingProtocol: Sweep net; eventDate: 13/6/2013; **Record Level:** datasetID: SMNS_Hym_Pte_002487; institutionCode: SMNS
- **Type status:** Other material. **Occurrence:** catalogNumber: BOLD Sample ID: SMNS_40120; recordedBy: H.-J. Flügel; sex: male; lifeStage: adult; preparations: dry mounted; associatedSequences: GenBank: OL538081; **Taxon:** scientificName: *Psiloceracrassispina* (Thomson, 1878); **Location:** continent: Europe; country: Germany; countryCode: DE; stateProvince: Hessen; locality: Lkr. Schwalm-Eder-Kreis, Neumorschen, Halberg, 5248c; verbatimElevation: 196 m; decimalLatitude: 51.0628; decimalLongitude: 9.6022; **Identification:** identifiedBy: M. Haas; dateIdentified: 2020; **Event:** samplingProtocol: Malaise trap; eventDate: 1/6 - 10/6/2013; **Record Level:** datasetID: SMNS_Hym_Pte_003055; institutionCode: SMNS
- **Type status:** Other material. **Occurrence:** catalogNumber: BOLD Sample ID: SMNS_40122; recordedBy: H.-J. Flügel; sex: male; lifeStage: adult; preparations: dry mounted; associatedSequences: GenBank: OL538153; **Taxon:** scientificName: *Psiloceracrassispina* (Thomson, 1878); **Location:** continent: Europe; country: Germany; countryCode: DE; stateProvince: Hessen; locality: Lkr. Schwalm-Eder-Kreis, Neumorschen, Halberg, 5248c; verbatimElevation: 196 m; decimalLatitude: 51.0628; decimalLongitude: 9.6022; **Identification:** identifiedBy: M. Haas; dateIdentified: 2020; **Event:** samplingProtocol: Malaise trap; eventDate: 1/6 - 10/6/2013; **Record Level:** datasetID: SMNS_Hym_Pte_003057; institutionCode: SMNS
- **Type status:** Other material. **Occurrence:** catalogNumber: BOLD Sample ID: SMNS_40126; recordedBy: H.-J. Flügel; sex: male; lifeStage: adult; preparations: dry mounted; associatedSequences: GenBank: OL538135; **Taxon:** scientificName: *Psiloceracrassispina* (Thomson, 1878); **Location:** continent: Europe; country: Germany; countryCode: DE; stateProvince: Hessen; locality: Lkr. Schwalm-Eder-Kreis, Neumorschen, Halberg, 5248c; verbatimElevation: 196 m; decimalLatitude: 51.0628; decimalLongitude: 9.6022; **Identification:** identifiedBy: M. Haas; dateIdentified: 2020; **Event:** samplingProtocol: Malaise trap; eventDate: 1/6 - 10/6/2013; **Record Level:** datasetID: SMNS_Hym_Pte_003061; institutionCode: SMNS
- **Type status:** Other material. **Occurrence:** catalogNumber: BOLD Sample ID: SMNS_40305; recordedBy: H.-J. Flügel; sex: male; lifeStage: adult; preparations: dry mounted; associatedSequences: GenBank: OL538122; **Taxon:** scientificName: *Psiloceracrassispina* (Thomson, 1878); **Location:** continent: Europe; country: Germany; countryCode: DE; stateProvince: Hessen; locality: Lkr. Schwalm-Eder-Kreis, Neumorschen, Halberg, 5233a; verbatimElevation: 196 m; decimalLatitude: 51.0628; decimalLongitude: 9.6022; **Identification:** identifiedBy: M. Haas; dateIdentified: 2020; **Event:** samplingProtocol: Malaise trap; eventDate: 3/5 - 1/6/2013; **Record Level:** datasetID: SMNS_Hym_Pte_003240; institutionCode: SMNS
- **Type status:** Other material. **Occurrence:** catalogNumber: BOLD Sample ID: SMNS_40311; recordedBy: H.-J. Flügel; sex: male; lifeStage: adult; preparations: dry mounted; associatedSequences: GenBank: OL538118; **Taxon:** scientificName: *Psiloceracrassispina* (Thomson, 1878); **Location:** continent: Europe; country: Germany; countryCode: DE; stateProvince: Hessen; locality: Lkr. Schwalm-Eder-Kreis, Neumorschen, Halberg, 5233a; verbatimElevation: 196 m; decimalLatitude: 51.0628; decimalLongitude: 9.6022; **Identification:** identifiedBy: M. Haas; dateIdentified: 2020; **Event:** samplingProtocol: Malaise trap; eventDate: 3/5 - 1/6/2013; **Record Level:** datasetID: SMNS_Hym_Pte_003246; institutionCode: SMNS
- **Type status:** Other material. **Occurrence:** catalogNumber: BOLD Sample ID: SMNS_41085; recordedBy: T. Kothe, M. Engelhardt, C. König; sex: female; lifeStage: adult; preparations: dry mounted; associatedSequences: GenBank: OL538056; **Taxon:** scientificName: *Psiloceracrassispina* (Thomson, 1878); **Location:** continent: Europe; country: Germany; countryCode: DE; stateProvince: Baden-Württemberg; locality: Tübingen, Hirschau, Wiesenweingärten, Flurstücknummer 3923, MF 9; verbatimElevation: 382 m; decimalLatitude: 48.5043; decimalLongitude: 8.9956; **Identification:** identifiedBy: M. Haas; dateIdentified: 2020; **Event:** samplingProtocol: Malaise trap; eventDate: 31/7 - 12/9/2014; **Record Level:** datasetID: SMNS_Hym_Pte_004020; institutionCode: SMNS

##### Ecological interactions

###### Parasite of

The host of the species is unknown, but is associated with Asteraceae (*Carduuspycnocephalus* L.) and Poaceae (*Hordeumleporinum* L.). Other members of the genus are reported from various hosts in Coleoptera and Lepidoptera.

##### Distribution

Widespread in Europe incl. United Kingdom and western Asia; Germany: Baden-Württemberg, Hessen

##### Notes

**Figure 37.**
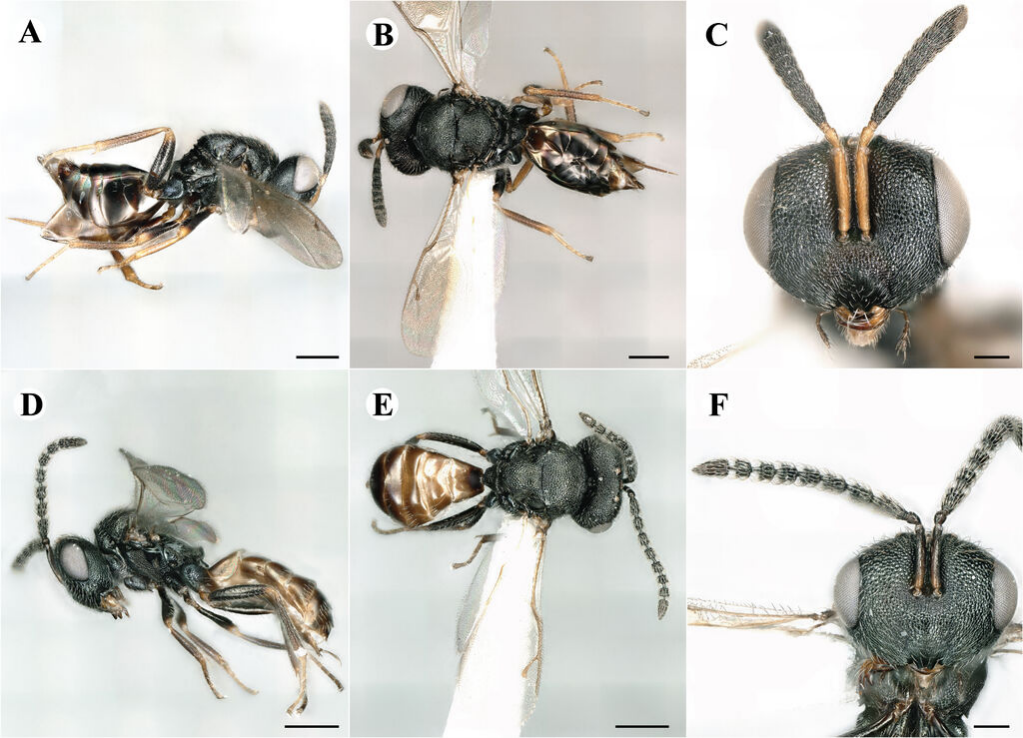
Digital microscopic images of *Psiloceracrassispina* (Thomson, 1878) (♀, Pte_001352: **A - C**; ♂, Pte_003246: **D - F**). **A, D** Habitus lateral **B, E** Habitus dorsal **C, F** Head frontal. Scale bars: **A, B, D, E** 500 µm; **C, F** 200 µm.

Newly-recorded species in Germany. Specimens are morphologically identifiable as *Psiloceracrassispina*, but barcode clustering is not consistent. *Psiloceragrahami* Ozdikmen, 2011 from Estland clusters within *P.crassispina* and males that are identifiable to *P.crassispina* cluster outside the main cluster of *P.crassispina*. The genus needs revision. Images: Fig. 37.

#### Psychophagoides crassicornis

Graham, 1969

6E6783E0-3E81-5F1D-8C68-41831AE464A6

##### Materials

- **Type status:** Other material. **Occurrence:** recordedBy: L. Krogmann, T. Kothe; sex: female; lifeStage: adult; preparations: dry mounted; **Taxon:** scientificName: *Psychophagoidescrassicornis* Graham, 1969; **Location:** continent: Europe; country: Germany; countryCode: DE; stateProvince: Baden-Württemberg; locality: Strohgäu, Markgröningen, EVS-Grundstück im Leudelsbachtal; verbatimElevation: 270 m; decimalLatitude: 48.9147; decimalLongitude: 9.0864; **Identification:** identifiedBy: H. Baur; dateIdentified: 2014; **Event:** samplingProtocol: Malaise trap; eventDate: 19/7 - 1/8/2011; **Record Level:** datasetID: SMNS_Hym_Pte_000685; institutionCode: SMNS
- **Type status:** Other material. **Occurrence:** catalogNumber: BOLD Sample ID: SMNS_40904; recordedBy: J. Aronov; sex: female; lifeStage: adult; preparations: dry mounted; associatedSequences: GenBank: OL538134; **Taxon:** scientificName: *Psychophagoidescrassicornis* Graham, 1969; **Location:** continent: Europe; country: Germany; countryCode: DE; stateProvince: Baden-Württemberg; locality: Stuttgart, Wilhelma, Futtergarten; verbatimElevation: 238 m; decimalLatitude: 48.8040; decimalLongitude: 9.2052; **Identification:** identifiedBy: M. Haas; dateIdentified: 2019; **Event:** samplingProtocol: Malaise trap; eventDate: 16/6 - 1/7/2014; **Record Level:** datasetID: SMNS_Hym_Pte_003839; institutionCode: SMNS
- **Type status:** Other material. **Occurrence:** catalogNumber: BOLD Sample ID: SMNS_40909; recordedBy: J. Aronov; sex: female; lifeStage: adult; preparations: dry mounted; associatedSequences: GenBank: OL538123; **Taxon:** scientificName: *Psychophagoidescrassicornis* Graham, 1969; **Location:** continent: Europe; country: Germany; countryCode: DE; stateProvince: Baden-Württemberg; locality: Stuttgart, Wilhelma, Futtergarten; verbatimElevation: 238 m; decimalLatitude: 48.8040; decimalLongitude: 9.2052; **Identification:** identifiedBy: M. Haas; dateIdentified: 2019; **Event:** samplingProtocol: Malaise trap; eventDate: 16/6 - 1/7/2014; **Record Level:** datasetID: SMNS_Hym_Pte_003844; institutionCode: SMNS

##### Ecological interactions

###### Parasite of

The host of the species is unknown.

##### Distribution

Northern Europe incl. United Kingdom; Germany: Baden-Württemberg

##### Notes

**Figure 38.**
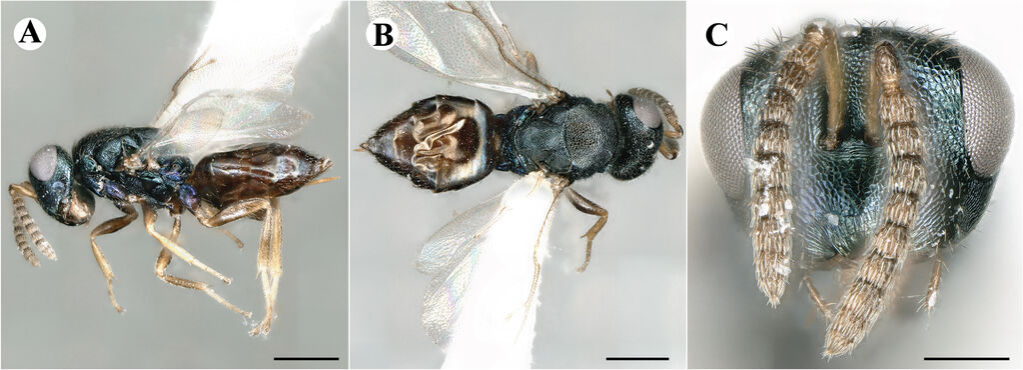
Digital microscopic images of *Psychophagoidescrassicornis* Graham, 1969 (♀, Pte_000658). **A** Habitus lateral **B** Habitus dorsal **C** Head frontal. Scale bars: **A, B** 500 µm; **C** 200 µm.

Newly-recorded genus and species in Germany. Images: Fig. 38.

#### Pteromalus altus

(Walker, 1834)

67B89164-5F08-5031-829B-0B5167CD7D1C

##### Materials

- **Type status:** Other material. **Occurrence:** catalogNumber: BOLD Sample ID: SMNS_39299; recordedBy: L. Krogmann, T. Kothe; sex: male; lifeStage: adult; preparations: dry mounted; associatedSequences: GenBank: OL538065; **Taxon:** scientificName: *Pteromalusaltus* (Walker, 1834); **Location:** continent: Europe; country: Germany; countryCode: DE; stateProvince: Baden-Württemberg; locality: Lkr. Main-Tauber-Kreis, Tauberbischofsheim, ex Truppenübungsplatz; verbatimElevation: 331 m; decimalLatitude: 49.6245; decimalLongitude: 9.7081; **Identification:** identifiedBy: H. Baur; dateIdentified: 2016; **Event:** samplingProtocol: Sweep net; eventDate: 12/6/2013; **Record Level:** datasetID: SMNS_Hym_Pte_002236; institutionCode: SMNS

##### Ecological interactions

###### Parasite of

The host of the species is unknown, but is associated with Euphorbiaceae (*Euphorbiaamygdaloides* L.). Other members of the genus are reported to be parasitoids of a wide variety of hosts in many insect orders.

##### Distribution

Europe incl. United Kingdom; Germany: Baden-Württemberg

##### Notes

**Figure 39.**
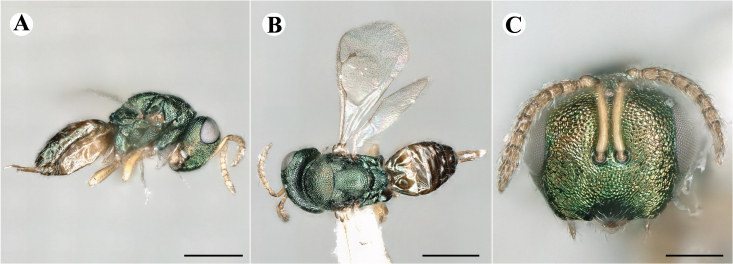
Digital microscopic images of *Pteromalusaltus* (Walker, 1834) (♂, Pte_002236). **A** Habitus lateral **B** Habitus dorsal **C** Head frontal. Scale bars: **A, B** 500 µm; **C** 200 µm.

Newly-recorded species in Germany. Images: Fig. 39.

#### Rohatina inermis

Bouček, 1954

B7A5298A-6FAE-50C1-9B3E-2D7188CF4D52

##### Materials

- **Type status:** Other material. **Occurrence:** catalogNumber: BOLD Sample ID: SMNS_37296; recordedBy: L. Krogmann, T. Kothe; sex: female; lifeStage: adult; preparations: dry mounted; associatedSequences: GenBank: OL538148; **Taxon:** scientificName: *Rohatinainermis* Bouček, 1954; **Location:** continent: Europe; country: Germany; countryCode: DE; stateProvince: Baden-Württemberg; locality: Strohgäu, Markgröningen, EVS-Grundstück im Leudelsbachtal; verbatimElevation: 270 m; decimalLatitude: 48.9147; decimalLongitude: 9.0864; **Identification:** identifiedBy: L. Krogmann; dateIdentified: 2014; **Event:** samplingProtocol: Malaise trap; eventDate: 5/6 - 19/6/2011; **Record Level:** datasetID: SMNS_Hym_Pte_000233; institutionCode: SMNS
- **Type status:** Other material. **Occurrence:** catalogNumber: BOLD Sample ID: SMNS_37345; recordedBy: L. Krogmann, T. Kothe; sex: male; lifeStage: adult; preparations: dry mounted; associatedSequences: GenBank: OL538068; **Taxon:** scientificName: *Rohatinainermis* Bouček, 1954; **Location:** continent: Europe; country: Germany; countryCode: DE; stateProvince: Baden-Württemberg; locality: Strohgäu, Markgröningen, EVS-Grundstück im Leudelsbachtal; verbatimElevation: 270 m; decimalLatitude: 48.9147; decimalLongitude: 9.0864; **Identification:** identifiedBy: L. Krogmann; dateIdentified: 2014; **Event:** samplingProtocol: Malaise trap; eventDate: 5/6 - 19/6/2011; **Record Level:** datasetID: SMNS_Hym_Pte_000282; institutionCode: SMNS
- **Type status:** Other material. **Occurrence:** catalogNumber: BOLD Sample ID: SMNS_37734; recordedBy: L. Krogmann, T. Kothe; sex: female; lifeStage: adult; preparations: dry mounted; associatedSequences: GenBank: OL538095; **Taxon:** scientificName: *Rohatinainermis* Bouček, 1954; **Location:** continent: Europe; country: Germany; countryCode: DE; stateProvince: Baden-Württemberg; locality: Strohgäu, Markgröningen, EVS-Grundstück im Leudelsbachtal; verbatimElevation: 270 m; decimalLatitude: 48.9147; decimalLongitude: 9.0864; **Identification:** identifiedBy: L. Krogmann; dateIdentified: 2014; **Event:** samplingProtocol: Malaise trap; eventDate: 19/7 - 1/8/2011; **Record Level:** datasetID: SMNS_Hym_Pte_000671; institutionCode: SMNS
- **Type status:** Other material. **Occurrence:** catalogNumber: BOLD Sample ID: SMNS_37853; recordedBy: L. Krogmann, T. Kothe; sex: male; lifeStage: adult; preparations: dry mounted; associatedSequences: GenBank: OL538090; **Taxon:** scientificName: *Rohatinainermis* Bouček, 1954; **Location:** continent: Europe; country: Germany; countryCode: DE; stateProvince: Baden-Württemberg; locality: Strohgäu, Markgröningen, EVS-Grundstück im Leudelsbachtal; verbatimElevation: 270 m; decimalLatitude: 48.9147; decimalLongitude: 9.0864; **Identification:** identifiedBy: H. Baur; dateIdentified: 2014; **Event:** samplingProtocol: Malaise trap; eventDate: 1/8 - 15/8/2011; **Record Level:** datasetID: SMNS_Hym_Pte_000790; institutionCode: SMNS

##### Ecological interactions

###### Parasite of

The host of the species is unknown.

##### Distribution

Northern and eastern Europe incl. United Kingdom; Germany: Baden-Württemberg

##### Notes

**Figure 40.**
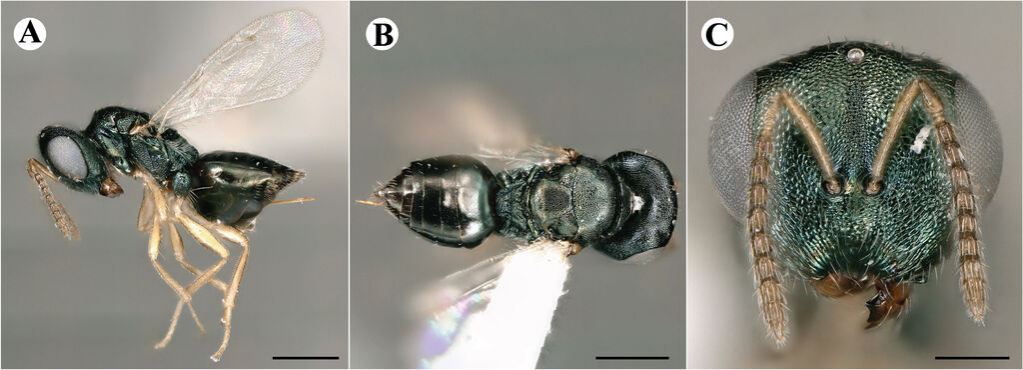
Digital microscopic images of *Rohatinainermis* Bouček, 1954 (♀, Pte_000233). **A** Habitus lateral **B** Habitus dorsal **C** Head frontal. Scale bars: **A, B** 500 µm; **C** 200 µm.

Newly-recorded genus and species in Germany. The males of *R.inermis* are not known so far. Within the genus, the only males connected to their females is *Rohatinamonstrosa* Boucek 1954. Via barcodes, the males and females of *R.inermis* could be matched. Images: Fig. 40.

#### Rohatina monstrosa

Bouček, 1954

60D78C18-5B90-5CBD-A1E9-2C03DE013E7B

##### Materials

- **Type status:** Other material. **Occurrence:** recordedBy: T. Kothe, M. Engelhardt, C. König; sex: female; lifeStage: adult; preparations: dry mounted; **Taxon:** scientificName: *Rohatinamonstrosa* Bouček, 1954; **Location:** continent: Europe; country: Germany; countryCode: DE; stateProvince: Baden-Württemberg; locality: Lkr. Tübingen, Steinenberg, Malaisefalle 2, Flurstücknummer 2906; verbatimElevation: 471 m; **Identification:** identifiedBy: M. Haas; dateIdentified: 2021; **Event:** samplingProtocol: Malaise trap; eventDate: 31/7 - 14/8/2014; **Record Level:** datasetID: SMNS_Hym_Pte_012431; institutionCode: SMNS

##### Ecological interactions

###### Parasite of

The host of the species is unknown.

##### Distribution

Northern, eastern and western Europe; Germany: Baden-Württemberg

##### Notes

**Figure 41.**
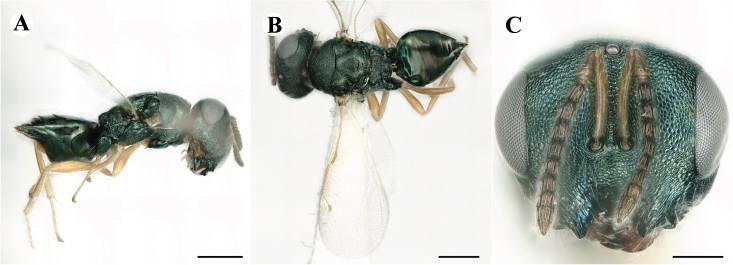
Digital microscopic images of *Rohatinamonstrosa* Bouček, 1954 (♀, Pte_012431). **A** Habitus lateral **B** Habitus dorsal **C** Head frontal. Scale bars: **A, B** 500 µm; **C** 200 µm. **A - B).**

Newly-recorded genus and species in Germany. No Barcode available. Images: Fig. 41.

#### Stichocrepis armata

Förster, 1860

CAD0975D-276B-57D7-9FF1-EECE68DE9A39

##### Materials

- **Type status:** Other material. **Occurrence:** catalogNumber: BOLD Sample ID: SMNS_41033; recordedBy: T. Kothe, M. Engelhardt, C. König; sex: male; lifeStage: adult; preparations: dry mounted; associatedSequences: GenBank: OL538062; **Taxon:** scientificName: *Stichocrepisarmata* Förster, 1860; **Location:** continent: Europe; country: Germany; countryCode: DE; stateProvince: Baden-Württemberg; locality: Tübingen, Hirschau, Wiesenweingärten, Flurstücknummer 3923, MF 9; verbatimElevation: 382 m; decimalLatitude: 48.5043; decimalLongitude: 8.9956; **Identification:** identifiedBy: M. Haas; dateIdentified: 2019; **Event:** samplingProtocol: Malaise trap; eventDate: 31/7 - 12/9/2014; **Record Level:** datasetID: SMNS_Hym_Pte_003968; institutionCode: SMNS

##### Ecological interactions

###### Parasite of

The species was reported as a parasitoid of Lepidoptera pupae (Geometridae: *Semiothisaliturata* (Clerck, 1759) and Sesiidae: *Synanthedonscoliaeformis* (Borkhausen, 1789)).

##### Distribution

Central and eastern Europe, northern and eastern Asia; Germany: Baden-Württemberg

##### Notes

**Figure 42.**
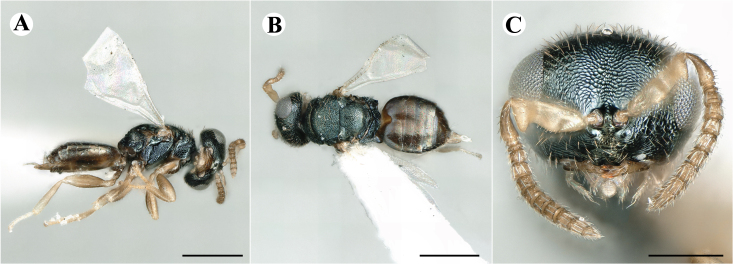
Digital microscopic images of *Stichocrepisarmata* Foerster, 1860 (♂, Pte_003968). **A** Habitus lateral **B** Habitus dorsal **C** Head frontal. Scale bars: **A, B** 500 µm; **C** 200 µm.

Newly-recorded genus and species in Germany. Images: Fig. 42.

#### Toxeuma discretum

Graham, 1984

E16D03F8-7B13-5D66-B0C2-D5801BF40350

##### Materials

- **Type status:** Other material. **Occurrence:** catalogNumber: BOLD Sample ID: SMNS_38364; recordedBy: L. Krogmann, T. Kothe, J. Reibnitz; sex: male; lifeStage: adult; preparations: dry mounted; associatedSequences: GenBank: OL538116; **Taxon:** scientificName: *Toxeumadiscretum* Graham, 1984; **Location:** continent: Europe; country: Germany; countryCode: DE; stateProvince: Baden-Württemberg; locality: Alb-Donau-Kreis, NSG Kleines Lautertal; verbatimElevation: 627 m; decimalLatitude: 48.453817; decimalLongitude: 9.84115; **Identification:** identifiedBy: M. Haas; dateIdentified: 2021; **Event:** samplingProtocol: Sweep net; eventDate: 13/6/2013; **Record Level:** datasetID: SMNS_Hym_Pte_001301; institutionCode: SMNS
- **Type status:** Other material. **Occurrence:** catalogNumber: BOLD Sample ID: SMNS_38372; recordedBy: L. Krogmann, T. Kothe, J. Reibnitz; sex: male; lifeStage: adult; preparations: dry mounted; associatedSequences: GenBank: OL538101; **Taxon:** scientificName: *Toxeumadiscretum* Graham, 1984; **Location:** continent: Europe; country: Germany; countryCode: DE; stateProvince: Baden-Württemberg; locality: Alb-Donau-Kreis, NSG Kleines Lautertal; verbatimElevation: 627 m; decimalLatitude: 48.453817; decimalLongitude: 9.84115; **Identification:** identifiedBy: M. Haas; dateIdentified: 2021; **Event:** samplingProtocol: Sweep net; eventDate: 13/6/2013; **Record Level:** datasetID: SMNS_Hym_Pte_001309; institutionCode: SMNS
- **Type status:** Other material. **Occurrence:** catalogNumber: BOLD Sample ID: SMNS_38379; recordedBy: L. Krogmann, T. Kothe, J. Reibnitz; sex: female; lifeStage: adult; preparations: dry mounted; associatedSequences: GenBank: OL538140; **Taxon:** scientificName: *Toxeumadiscretum* Graham, 1984; **Location:** continent: Europe; country: Germany; countryCode: DE; stateProvince: Baden-Württemberg; locality: Alb-Donau-Kreis, NSG Kleines Lautertal; verbatimElevation: 627 m; decimalLatitude: 48.453817; decimalLongitude: 9.84115; **Identification:** identifiedBy: M. Moser; dateIdentified: 2017; **Event:** samplingProtocol: Sweep net; eventDate: 13/6/2013; **Record Level:** datasetID: SMNS_Hym_Pte_001316; institutionCode: SMNS
- **Type status:** Other material. **Occurrence:** catalogNumber: BOLD Sample ID: SMNS_38763; recordedBy: L. Krogmann, T. Kothe; sex: female; lifeStage: adult; preparations: dry mounted; associatedSequences: GenBank: OL538112; **Taxon:** scientificName: *Toxeumadiscretum* Graham, 1984; **Location:** continent: Europe; country: Germany; countryCode: DE; stateProvince: Baden-Württemberg; locality: Lkr. Breisgau-Hochschwarzwald, NSG Badberg; verbatimElevation: 426 m; decimalLatitude: 48.096389; decimalLongitude: 7.680833; **Identification:** identifiedBy: M. Haas; dateIdentified: 2021; **Event:** samplingProtocol: Sweep net; eventDate: 18/6/2013; **Record Level:** datasetID: SMNS_Hym_Pte_001700; institutionCode: SMNS
- **Type status:** Other material. **Occurrence:** catalogNumber: BOLD Sample ID: SMNS_39285; recordedBy: L. Krogmann, T. Kothe; sex: female; lifeStage: adult; preparations: dry mounted; associatedSequences: GenBank: OL538129; **Taxon:** scientificName: *Toxeumadiscretum* Graham, 1984; **Location:** continent: Europe; country: Germany; countryCode: DE; stateProvince: Baden-Württemberg; locality: Lkr. Main-Tauber-Kreis, Tauberbischofsheim, ex Truppenübungsplatz; verbatimElevation: 331 m; decimalLatitude: 49.624467; decimalLongitude: 9.708083; **Identification:** identifiedBy: M. Haas; dateIdentified: 2021; **Event:** samplingProtocol: Sweep net; eventDate: 12/6/2013; **Record Level:** datasetID: SMNS_Hym_Pte_002222; institutionCode: SMNS
- **Type status:** Other material. **Occurrence:** catalogNumber: BOLD Sample ID: SMNS_39292; recordedBy: L. Krogmann, T. Kothe; sex: male; lifeStage: adult; preparations: dry mounted; associatedSequences: GenBank: OL538150; **Taxon:** scientificName: *Toxeumadiscretum* Graham, 1984; **Location:** continent: Europe; country: Germany; countryCode: DE; stateProvince: Baden-Württemberg; locality: Lkr. Main-Tauber-Kreis, Tauberbischofsheim, ex Truppenübungsplatz; verbatimElevation: 331 m; decimalLatitude: 49.624467; decimalLongitude: 9.708083; **Identification:** identifiedBy: M. Haas; dateIdentified: 2021; **Event:** samplingProtocol: Sweep net; eventDate: 12/6/2013; **Record Level:** datasetID: SMNS_Hym_Pte_002229; institutionCode: SMNS

##### Ecological interactions

###### Parasite of

The host of the species is unknown, but is associated with Poaceae (*Helictotrichon* sp.). Other members of the genus are reported to be parasitoids of Diptera (Agromyzidae).

##### Distribution

Scattered central and northern Europe incl. United Kingdom; Germany: Baden-Württemberg

##### Notes

**Figure 43.**
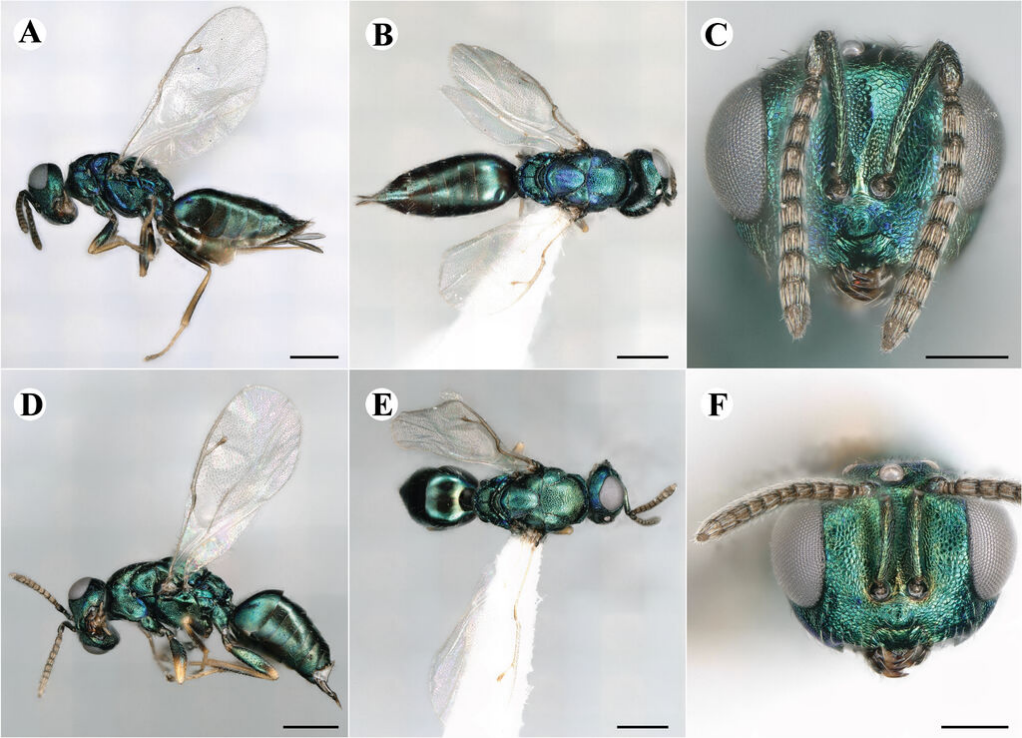
Digital microscopic images of *Toxeumadiscretum* Graham, 1984 (♀, Pte_001316: **A - C**, ♂, Pte_001309: **D - F**). **A, D** Habitus lateral **B, E** Habitus dorsal **C, F** Head frontal. Scale bars: **A, B, D, E** 500 µm; **C, F** 200 µm.

Newlyrecorded species in Germany. The males are morphologically close to *T.fuscicorne* (Graham, 1984). The colour matches the description of *T.discretum*. Images: Fig. 43.

#### Trigonoderus nobilitatus

Graham, 1993

753FBB2A-5FBD-5227-9332-AEA994EFDFB5

##### Materials

- **Type status:** Other material. **Occurrence:** catalogNumber: BOLD Sample ID: SMNS_39901; recordedBy: B. Rulik; sex: female; lifeStage: adult; preparations: dry mounted; associatedSequences: GenBank: OL538105; **Taxon:** scientificName: *Trigonoderusnobilitatus* Graham, 1993; **Location:** continent: Europe; country: Germany; countryCode: DE; stateProvince: Rheinland-Pfalz; locality: Kreis Ahrweiler, Niederzissen, Bausenberg, Trockenrasen, MF5, ZFMK-MAL-0001952; verbatimElevation: 321 m; decimalLatitude: 50.4684; decimalLongitude: 7.2220; **Identification:** identifiedBy: M. Haas; dateIdentified: 2015; **Event:** samplingProtocol: Malaise trap; eventDate: 25/7 - 8/8/2013; **Record Level:** datasetID: SMNS_Hym_Pte_002836; institutionCode: SMNS

##### Ecological interactions

###### Parasite of

The host of the species is unknown, but members of the genus are reported to be parasitoids of xylophagous Coleoptera (Cerambycidae and Scolytidae).

##### Distribution

Scattered in central and eastern Europe; Germany: Rheinland-Pfalz

##### Notes

**Figure 44.**
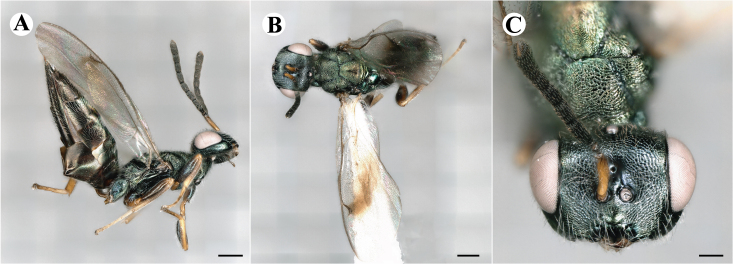
Digital microscopic images of *Trigonoderusnobilitatus* Graham, 1993 (♀, Pte_002836). **A** Habitus lateral **B** Habitus dorsal **C** Head frontal. Scale bars: **A, B** 500 µm; **C** 200 µm.

Newly-recorded species in Germany. Images: Fig. 44.

#### Trychnosoma punctipleura

(Thomson, 1878)

AB7F397B-44BB-55EF-A09D-B63495CA3C39

##### Materials

- **Type status:** Other material. **Occurrence:** catalogNumber: BOLD Sample ID: SMNS_39043; recordedBy: U. Hornig; sex: female; lifeStage: adult; preparations: dry mounted; associatedSequences: GenBank: OL538099; **Taxon:** scientificName: *Trychnosomapunctipleura* (Thomson, 1878); **Location:** continent: Europe; country: Germany; countryCode: DE; stateProvince: Sachsen; locality: Lkr. Görlitz, Oppach; verbatimElevation: 326 m; decimalLatitude: 53.0614; decimalLongitude: 11.4725; **Identification:** identifiedBy: M. Haas; dateIdentified: 2021; **Event:** samplingProtocol: Malaise trap; eventDate: 1/9 - 7/9/2013; **Record Level:** datasetID: SMNS_Hym_Pte_001980; institutionCode: SMNS

##### Ecological interactions

###### Parasite of

The species was reported as a parasitoid of Coleoptera (Curculionidae), associated with Betoideae (*Betavulgaris* L.) and various Pinaceae.

##### Distribution

Central, northern and eastern Europe incl. United Kingdom, western Asia; Germany: Sachsen

##### Notes

**Figure 45.**
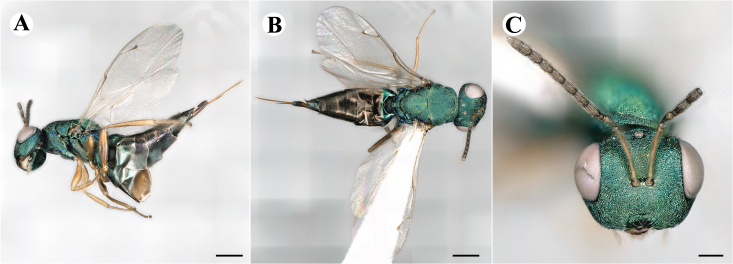
Digital microscopic images of *Trychnosomapunctipleura* (Thomson, 1878) (♀, Pte_001980). **A** Habitus lateral **B** Habitus dorsal **C** Head frontal. Scale bars: **A, B** 500 µm; **C** 200 µm.

Newly-recorded genus and species in Germany. Images: Fig. 45.

## Analysis

### Taxonomic results

In the taxon treatment section, we describe two previously unknown males of newly-recorded species in Germany. The males could be matched to their respective described female counterparts with barcoding data.

### Faunistic results

In the checklists section, we report the occurrence of 41 previously unrecorded species in Germany, belonging to six subfamilies of Pteromalidae (Table 3). This results in a total of 776 recorded species and 152 genera of Pteromalidae occurring in Germany. For all except four newly-recorded species, barcodes could be generated.

## Discussion

**Biology of Pteromalidae**

In the following, a short synopsis of the biology of each subfamily of Pteromalidae in which new species are reported will be given. Unless stated otherwise, mentions of host organisms and other general biological data is sourced from Graham (1969), Bouček and Rasplus (1991), Grissell and Schauff (1997), Noyes (2021).

**Ceinae Bouček, 1961**

Ceinae seem to be associated with leaf litter and humus-rich forest habitats, based on collection data and putative functional adaptations in morphology (Darling 1991). Specimens of this small subfamily are seldomly collected and, therefore, tend to be under-represented in collections. In Germany, only two species have been reported so far, both of which have emerged from mines of Dipteran hosts: *Ceapulicaris* Walker, 1837, which was observed emerging from a leaf mine of *Phytomyzapauliloewii* Hendel, 1920 (Diptera: Agromyzidae) on *Peucedanumoreoselinum* (L.) Moench (Apiaceae) and *Spalangiopeltaalata* Bouček, 1953, which was reared from a leaf mine of *Scaptomyzaflaveola* Meigen, 1830, now synonymised under *Scaptomyzaflava* (Fallen, 1823) (Diptera: Drosophilidae), on *Cakilemaritima* Scopoli (Brassicaceae) (Bouček 1961). Host records for all other species within this subfamily, including the third German species recorded herein, have yet to be observed to conclusively assess the true host range of Ceinae.

**Cleonyminae Walker, 1837**

This subfamily is represented with only few species in Germany and includes, with our new records, eleven species in nine genera. Cleonyminae are rarely encountered when sampling with standard techniques like sweep netting or malaise trapping. This can largely be attributed to their biology as primary parasitoids of wood-boring insects, requiring targeted manual sampling or rearing. Species from this subfamily are mainly reported to be associated with xylophagous beetles like Anobiidae, Buprestidae, Cerambycidae, Curculionidae, Scolytidae and others (Gibson 2003). This holds also true for the species recorded from Germany for which host associations have been reported, except for *Notanisussexramosus* (Erdös, 1946). *Notanisussexramosus* reportedly develops as a primary parasitoid on species of the chalcidoid family Eurytomidae developing in grass (Poales: Poaceae). Although collected fairly seldom with often only vague to no host associations known, Graham (1969) gives an interesting insight into the behaviour of a member of the Cleonyminae subfamily. He describes watching female *Cleonymuslaticornis* Walker, 1837 searching for a host on old trees, specifically *Salix* (Malphigiales: Salicaceae) and *Corylus* (Fagales: Coryloideae). Females occur especially on sunny days from May to June, patrolling along the surface in straight lines, turning abruptly “like a sentry on duty” (Graham 1969), probably sensing for vibrations caused by their hosts moving and feeding within the wood. Cleonyminae are, in general, rather slender in habitus, which might be an adaptation to facilitate emerging out of the wooden tunnels created by their hosts. Some genera, like *Heydenia* and *Oodera*, possess strongly enlarged front femora which are equipped with pegs and strong bristles. Werner and Peters (2018) hypothesise that these leg modifications ease burrowing through the tunnels, in conjunction with a high movability due to the slender body and elongated pronotum.

**Miscogastrinae Walker, 1833**

After Pteromalinae, Miscogastrinae is the second largest subfamily of Pteromalidae in Germany, now including 72 species in 16 genera. Biologically, the majority of the species is associated with Diptera, especially those developing in plants like Agromyzidae, Tephritidae, Drosophilidae and Cecidomyiidae. Records of Dipteran hosts, utilised by this subfamily, also include mainly saprophagous dipteran families, like Muscidae, Anthomyidae, Lauxaniidae and Scathophagidae. Only few species seem to attack hosts from other insect orders, like the northern European *Seladermaaeneum* (Walker, 1833) attacking Lepidoptera of the family Nepticulidae or the genera *Yusufia* and the newly-recorded *Ksenoplata* attacking Coleoptera of the family Curculionidae and Bruchidae, respectively. Especially some of the dipteran hosts can cause great damage when introduced to non-native regions; therefore, some Miscogastrinae like *Cyrtogastervulgaris* Walker, 1833 and *Halticopteradaci* Silvestri, 1914 have been used for biological control (Bouček and Rasplus 1991, Mohamed et al. 2016) or are considered promising candidates, for example, as potential antagonists to keep Australian Agromyzidae in check (Ridland et al. 2020).

Contrary to many other subfamilies in Pteromalidae, Miscogastrinae include many koinobiont endoparasitoid species, especially within the tribe of Miscogastrini (Parker and Thompson 1925). They attack the larval stage of Agromyzidae by ovipositing directly into their host’s body, allowing it to develop to the pupal stage before killing it and emerging. Parker and Thompson (1925) give a detailed description of the development of a not further identified species of *Miscogaster*. To their account, those female *Miscogaster* can be observed in early spring, depositing several eggs into one *Agromyza* sp. larva feeding inside a leaf. Apparently, a first wave of adult *Miscogaster* emerge in late spring, forming the first generation which will oviposit again the same year. Part of the eggs laid in early spring will, however, develop only so far that the host can pupate, allowing the parasitoid inside to be dormant until the following year. Adults emerge by gnawing their way out of the host pupa and leaf with their sharp mandibles. Although several eggs might have been deposited by the female, Parker and Thompson (1925) report only a single adult emerging from each host.

**Ormocerinae Walker, 1833**

Ormocerinae is, with nine species and three genera, a rather small subfamily of Pteromalidae in Germany, though far more species-rich in the Southern Hemisphere, for example, Australasia (Bouček 1988). Species of Ormocerinae are mostly associated with gall-inducing hosts and have been reported to be gall-inducers themselves, making those species phytophagous (Bouček 1988, Berry and Withers 2002, Prinsloo and Neser 2007), a very uncommon biology for Pteromalidae. The exact relationship with the gall-inducing host is often difficult to ascertain, which is why it is possible that even species with a potential host association can rather be inquilines feeding on the plant tissue than on the gall-inducer itself (La Salle 2005). All species in Germany whose hosts are known, however, appear to be non-gall inducing themselves. The genus *Ormocerus* attacks Cynipidae (Hymenoptera: Cynipoidea), *Semiotellus* is associated with Cecidomyiidae (Diptera) in grasses (Poales: Poaceae) and *Systasis* utilises a wide variety of hosts including Coleoptera (Apionidae, Bruchidae, Curculionidae), Diptera (Agromyzidae, Cecidomyiidae, Tephritidae), Hymenoptera (Cynipidae) and even, although challenged by Graham (1969), Lepidoptera (Tortricidae). For the species *Systasisencyrtoides* Walker, 1834, which is also part of the German fauna, it has been reported that they are predatory as larvae, killing multiple hosts during their own development, instead of being true parasitoids utilising only one (Parnell 1963).

**Pireninae Haliday, 1844**

With 35 species and five genera, Pireninae represent the third largest subfamily in Germany. Most of those species whose hosts are known are associated with the gall-inducing Cecidomyiidae (Diptera), aside from few exceptions attacking Agromyzidae or the monotypic genus *Termolampa* utilising Tortricidae (Lepidoptera) on Pinaceae (Coniferales). Some species have been recognised as potent antagonists to agricultural pests, for example, the small egg parasitoid *Macroglenespenetrans* (Kirby, 1800), parasitising the formerly Palaearctic wheat-midge *Sitodiplosismosellana* (Géhin, 1857) which was introduced to northern America and developed as an invasive species (Doane et al. 1989, Olfert et al. 2009).

**Pteromalinae Dalman, 1820**

The subfamily of Pteromalinae is, by far, the most species-rich of Pteromalidae in Germany, now comprising 618 species. This tremendous species richness is also reflected in their varied biology. Whereas other pteromalid subfamilies are mostly united by a certain host or host biology, Pteromalinae exhibit a wide range of host organisms and modes of a parasitoid or predatory lifestyle, which might be more conserved in tribes or species groups (Bouček and Rasplus 1991). Most Pteromalinae are associated with holometabolous insect orders, being primary parasitoids, hyperparasitoids or predators, but no phytophagous species are known to date. In the following, different life history strategies will shortly be presented to give a sense of their diversity in Pteromalinae.

Ectoparasitoids

The majority of pteromalids are reported to be ectoparasitoids (Gómez and Luis 2012, Noyes 2021). This mode of parasitism is mostly linked to idiobionts which do not allow their host to develop any further than at the time of oviposition (Quicke 1997). This is reasonable, because ectoparasitoids are very exposed and vulnerable to their hosts' efforts of mechanically ridding themselves of them. This is especially true if they form larger groups of several developing larvae due to their gregarious nature. This vulnerability is, however, often mitigated just like solitary ectoparasitoids by utilising hosts and host developmental stages that allow the parasitoid to feed in a concealed environment, for example, between the pupating host and the outer puparium wall or within a plant seed. Examples of species with this biology include *Nasoniavitripennis* (Walker, 1836), *Dibrachysmiscogastri* (Bouche, 1834), *Lariophagusdistinguendus* (Foerster, 1841), *Spintherusdubius* (Nees, 1834) or *Tritneptisklugii* (Ratzeburg, 1844). From this list, *D.miscogastri* is especially noteworthy as it is an extremely polyphagous species with 35+ recorded hosts from at least five orders, being a primary parasitoid or hyperparasitoid of pupae (Peters and Baur 2011), exemplifying the immense adaptability of ectoparasitoid species.

A peculiar type of behaviour, reported from several ectoparasitoid Pteromalinae parasitising concealed hosts, is the practice of host-feeding of the female via the construction of a feeding tube to gather haemolymph directly from the encased host (e.g. Fulton 1933, King and Ratcliffe 1969, Jervis and Kidd 1986). Host feeding ensures the acquisition of nutrients for egg maturation and longevity, but is hard to achieve with concealed hosts and without the adaptation to feed on non-host organisms potentially more easily available (Quicke 1997). This tube-building behaviour is, however, not exclusive to the subfamily Pteromalinae, but can also be found amongst the chalcidoid families Aphelinidae, Encyrtidae, Eulophidae, Eupelmidae, Eurytomidae, Signiphoridae and Torymidae, as well as in the superfamily Ichneumonoidea (Jervis and Kidd 1986).

In general, ectoparasitoid larvae are morphologically rather simple, possessing a 13-segmented uniform body, with a rudimentary head capsule and stiletto-like mandibles for piercing the host’s body (Parker and Thompson 1925, Krogmann and Abraham 2003, Gómez and Luis 2012). Those larvae are sedentary feeders on their hosts, lacking any form of appendages besides some bristles, thus limiting their locomotion.

Endoparasitoids

Contrary to ectoparasitoids, endoparasitoids rather attack exposed hosts and are generally koinobionts, allowing the host to develop further after oviposition (Quicke 1997). Although they are not in danger of mechanical removal of the host, developing in its body, endoparasitoids have to overcome its host’s immune system, often leading to a higher degree of specialisation (Quicke 1997).

One of the best-known species of endoparasitoid Pteromalidae is the cosmopolitan *Pteromaluspuparum* (Linnaeus, 1758), predominantly developing gregariously in pupae of Lepidoptera of various families.

Parker and Thompson (1925) state that larvae of endoparasitoids are mostly of a caudate form, being slender and possessing an elongated tail, potentially facilitating locomotion in the haemolymph of their hosts. Their statement, however, encompasses Chalcidoidea in general and is not restricted to Pteromalidae, let alone Pteromalinae. For example, the larva of the pteromaline species *Tomicobiaseitneri* (Ruschka, 1924), a common parasitoid of the economically and ecologically relevant *Ips typographus* (Linnaeus, 1758) (Coleoptera: Curculionidae), are of the caudate form (Georgiev and Takov 2005, Wegensteiner et al. 2017). Parker and Thompson (1925), however, also mention the exception to their statement with *P.puparum*, developing from larvae closely resembling those of ectoparasitoid Pteromalinae.

Predators

A predatory biology, where several hosts/prey are killed during the development, is seldom recorded within Pteromalinae. Known examples are the larvae of *Mesopolobusaequus* (Walker, 1834) and *M.graminum* (Hardh, 1950), being predators of eggs and larvae in the stems of grass and *Pteromalusplatyphilus* Walker, 1874 being found in the egg sacs of spiders. Additionally, members of the former subfamily Panstenoninae, now part of Pteromalinae, were recorded to prey on homopteran eggs.

**Potential use of parasitoid Hymenoptera as indicators for nature conservation**

Although some species of Pteromalidae have been studied extensively, for many species, biological information is insufficiently confirmed or not existent at all, as is the case with most parasitoid groups. This fact severely hinders the utilisation of those species for nature conservation efforts. Their biology is often highly specific with a narrow host range and, therefore, their occurrence is tightly linked to their host organisms which, in turn, also demand certain biotic and abiotic conditions. Shaw and Hochberg (2001) already broadly discussed the neglect of parasitoid hymenopteran species in nature conservation efforts.

Despite a general lack of profound research in this field, Shaw (2006) argues that parasitoids are highly susceptible to a changing environment, for example, changing climatic conditions might lead to asynchronous life cycles between parasitoids and their hosts or agricultural practices, like the use of agrochemicals in conventional farming. More recent studies (Cook et al. 2016, Tappert et al. 2017) have shown that even smaller doses of neonicotinoid toxins, used for pest control, can cause severe disruptions in the mate-finding process of parasitoid wasps. Most recently, Villa-Galaviz et al. (2021) also reported higher rates of more general interactions within leaf miner-parasitoid networks in fertiliser-treated patches of land compared to non-treated patches. This is a result of host range expansion of single parasitoid species and/or a higher abundance of generalist parasitoids, leading to fewer parasitoid species interacting with higher numbers of host species. This highlights the profound changes which practices, like fertilising exert on parasitoid host communities. Community composition of parasitoids and functional traits can also differ significantly between habitats (Kendall and Ward 2016) and it has been shown that increased habitat fragmentation leads to a decrease in the number of parasitoid species and parasitisation rates (Kruess and Tscharntke 1994, Kruess 1996). Those often unknown effects could render parasitoids as valuable indicators with a potential for a predictive use in the future.

More research is needed to evaluate how parasitoids can be used as indicators, but current knowledge is already showing the promising potential of those highly-specialised species.

### Conclusion

The amount of newly-recorded species of Pteromalidae in Germany highlights the need to further advance species discovery, even in relatively well-studied areas. Preliminary results of our dataset also show that there is a strong need to taxonomically study the group and revise species and genera, based on integrating morphological, molecular and, if available, biological data. Advancing the knowledge of their biology is especially important due to their potentially high susceptibility to changes in the environment and their exceedingly high potential as indicators in nature conservation. Further expanding the barcoding databases for parasitoid groups is the foundation to allow the inclusion in molecular ecological studies, accelerating the gain of knowledge of their mostly enigmatic interactions and role in our ecosystems.

## Supplementary Material

XML Treatment for Rhicnocoelia impar

XML Treatment for Rohatina inermis

XML Treatment for Spalangiopelta dudichi

XML Treatment for Cleonymus brevis

XML Treatment for Cleonymus obscurus

XML Treatment for Halticoptera longipetiolus

XML Treatment for Ksenoplata quadrata

XML Treatment for Rhicnocoelia impar

XML Treatment for Tricyclomischus celticus

XML Treatment for Systasis annulipes

XML Treatment for Ecrizotes longicolis

XML Treatment for Ecrizotes monticola

XML Treatment for Gastrancistrus acutus

XML Treatment for Gastrancistrus affinis

XML Treatment for Gastrancistrus compressus

XML Treatment for Gastrancistrus fumipennis

XML Treatment for Macroglenes eximius

XML Treatment for Macroglenes paludum

XML Treatment for Micradelus acutus

XML Treatment for Acrocormus semifasciatus

XML Treatment for Apelioma pteromalinum

XML Treatment for Arthrolytus slovacus

XML Treatment for Atrichomalus trianellatus

XML Treatment for Coelopisthia pachycera

XML Treatment for Cryptoprymna paludicola

XML Treatment for Cyclogastrella clypealis

XML Treatment for Dibrachys hians

XML Treatment for Dinotoides tenebricus

XML Treatment for Erythromalus rufiventris

XML Treatment for Gbelcia crassiceps

XML Treatment for Heteroprymna longicornis

XML Treatment for Kaleva corynocera

XML Treatment for Platygerrhus unicolor

XML Treatment for Psilocera confusa

XML Treatment for Psilocera crassispina

XML Treatment for Psychophagoides crassicornis

XML Treatment for Pteromalus altus

XML Treatment for Rohatina inermis

XML Treatment for Rohatina monstrosa

XML Treatment for Stichocrepis armata

XML Treatment for Toxeuma discretum

XML Treatment for Trigonoderus nobilitatus

XML Treatment for Trychnosoma punctipleura

## Figures and Tables

**Figure 4.**
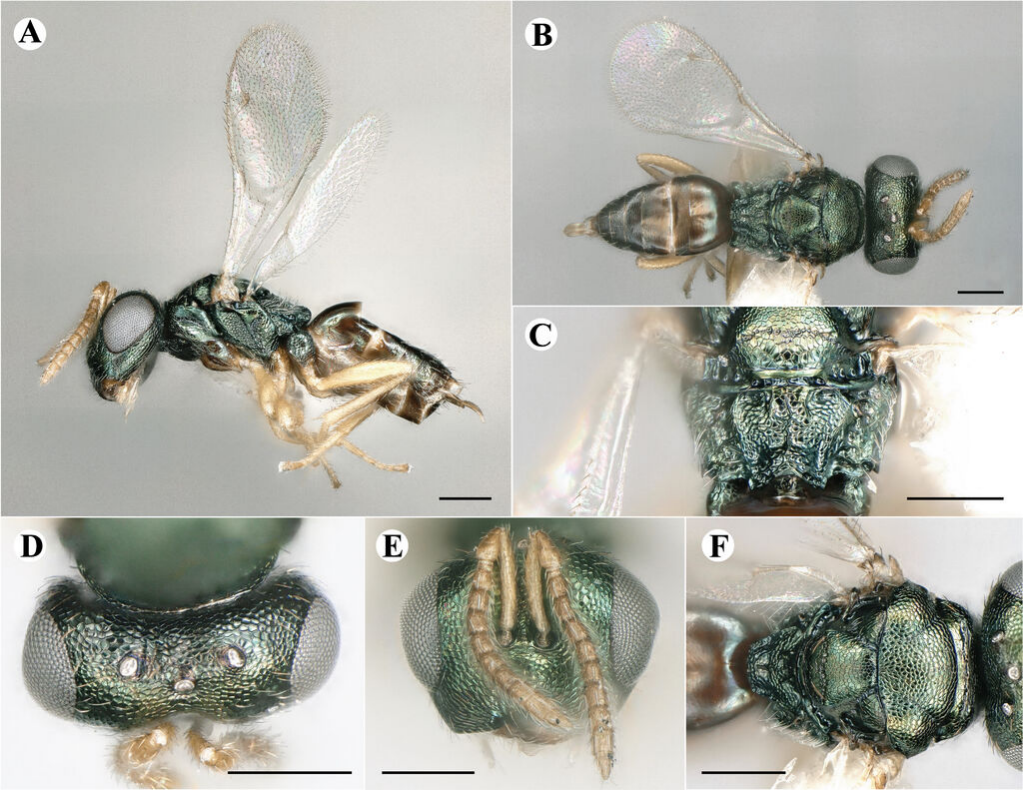
Digital microscopic image of the male *Rohatinainermis* Bouček, 1954 (Pte_000282). **A** Habitus lateral **B** Habitus dorsal **C** Metanotum and propodeum dorsal **D** Head dorsal **E** Head frontal **F** Mesosoma dorsal. Scale bars: **A - B** 200 µm.

**Table 1.** PCR cycler protocol for amplification of the COI barcoding gene of Pteromalidae.

Heat Lid	104°C/110°C		
Initial Denaturation	94°C	2‘	
Denaturation	96°C	1‘	5X
Annealing	45°C	1:30	
Elongation	72°C	1:30	
Denaturation	93°C	1‘	45X
Annealing	50°C	1:30	
Elongation	72°C	1:30	
Final Elongation	72°C	5‘	
Storage	4°C	∞	

**Table 2.** List of all newly-recorded species for Germany by subfamily.

**Subfamily**	**Species**	**New genus record**	**Associates known**	**Barcode available**	**Female**	**Male**	**Specimens total**
**Ceinae**	*Spalangiopeltadudichi* Erdös, 1955	No	No	No	1		1
**Cleonyminae**	*Cleonymusbrevis* Bouček, 1972	No	Yes	No	6		6
	*Cleonymusobscurus* Walker, 1837	No	Yes	Yes	2	2	4
**Miscogastrinae**	*Halticopteralongipetiolus* Hedqvist, 1975	No	Yes	Yes		2	2
	*Ksenoplataquadrata* Bouček, 1965	Yes	Yes	Yes	2		2
	*Rhicnocoeliaimpar* (Walker, 1836)	Yes	No	Yes	1	1	2
	*Tricyclomischuscelticus* Graham, 1956	Yes	No	Yes	3		3
**Ormocerinae**	*Systasisannulipes* (Walker, 1834)	No	Yes	Yes	1		1
**Pireninae**	*Ecrizoteslongicornis* (Walker, 1848)	Yes	No	Yes	1		1
	*Ecrizotesmonticola* Foerster, 1861	Yes	No	Yes	1		1
	*Gastrancistrusacutus* Walker, 1834	No	No	Yes	2		2
	*Gastrancistrusaffinis* Graham, 1969	No	Yes	Yes	1		1
	*Gastrancistruscompressus* Walker, 1834	No	Yes	Yes	3	3	6
	*Gastrancistrusfumipennis* Walker, 1834	No	No	Yes	1		1
	*Macrogleneseximius* (Haliday, 1833)	No	Yes	Yes		1	1
	*Macroglenespaludum* (Graham, 1969)	No	Yes	Yes		3	3
	*Micradelusacutus* Graham, 1969	Yes	No	Yes	1		1
**Pteromalinae**	*Acrocormussemifasciatus* Thomson, 1878	Yes	Yes	Yes	1		1
	*Apeliomapteromalinum* (Thomson, 1878)	Yes	Yes	Yes	3	1	4
	*Arthrolytusslovacus* Graham, 1969	No	No	Yes	1	1	2
	*Atrichomalustrianellatus* Graham, 1956	Yes	No	Yes	1		1
	*Coelopisthiapachycera* Masi, 1924	No	Yes	No	1		1
	*Cryptoprymnapaludicola* Askew, 1991	No	No	Yes	1	1	2
	*Cyclogastrellaclypealis* Bouček, 1965	No	No	Yes	13	3	16
	*Dibrachyshians* Bouček, 1965	No	Yes	Yes	1		1
	*Dinotoidestenebricus* (Walker, 1834)	Yes	Yes	Yes	8	2	10
	*Erythromalusrufiventris* (Walker, 1835)	Yes	No	Yes	2	1	3
	*Gbelciacrassiceps* Bouček, 1961	Yes	No	Yes	1		1
	*Heteroprymnalongicornis* (Walker, 1835)	Yes	No	Yes	2		2
	*Kalevacorynocera* Graham, 1957	Yes	Yes	Yes	5		5
	*Platygerrhusunicolor* Graham, 1969	No	No	Yes	2		2
	*Psiloceraconfusa* Graham, 1992	No	No	Yes	1		1
	*Psiloceracrassispina* (Thomson, 1878)	No	No	Yes	3	11	14
	*Psychophagoidescrassicornis* Graham, 1969	Yes	No	Yes	3		3
	*Pteromalusaltus* (Walker, 1834)	No	No	Yes		1	1
	*Rohatinainermis* Bouček, 1954	Yes	No	Yes	2	2	4
	*Rohatinamonstrosa* Bouček, 1954	Yes	No	No	1		1
	*Stichocrepisarmata* Förster, 1860	Yes	Yes	Yes		1	1
	*Toxeumadiscretum* Graham, 1984	No	Yes	Yes	3	3	6
	*Trigonoderusnobilitatus* Graham, 1993	No	No	Yes	1		1
	*Trychnosomapunctipleura* (Thomson, 1878)	Yes	Yes	Yes	1		1
	**Specimens total**				**83**	**39**	**122**

**Table 3.** Numbers of newly-recorded genera (New Gen.) and species (New Spp.) for Germany by subfamily, with total updated record numbers given, based on Noyes (2021) and other recent publications.

**Subfamily**	**New Gen.**	**New Spp.**	**Gen.** **GER**	**Spp. GER**
Asaphinae	-	-	2	3
Ceinae	-	1	2	3
Cerocephalinae	-	-	2	4
Cleonyminae	-	2	4	11
Colotrechninae	-	-	1	1
Diparinae	-	-	1	1
Eunotinae	-	-	3	8
Miscogastrinae	3	4	17	73
Ormocerinae	-	1	3	9
Pireninae	2	9	5	35
Pteromalinae	12	24	111	618
Spalangiinae	-	-	1	10
**Total**	**17**	**41**	**152**	**776**
